# Early Sitting in Ischemic Stroke Patients (SEVEL): A Randomized Controlled Trial

**DOI:** 10.1371/journal.pone.0149466

**Published:** 2016-03-29

**Authors:** Fanny Herisson, Sophie Godard, Christelle Volteau, Emilie Le Blanc, Benoit Guillon, Marie Gaudron, SEVEL study group

**Affiliations:** 1 Department of Neurology and Stroke Unit, Nantes University Hospital, Nantes, France; 2 Department of Neurology and Stroke Unit, Angers University Hospital, Angers, France; 3 Clinical Research Unit, Nantes University Hospital, Nantes, France; 4 Department of Neurology and Stroke Unit, Tours University Hospital, Tours France; University of Glasgow, UNITED KINGDOM

## Abstract

**Background:**

Extended immobility has been associated with medical complications during hospitalization. However no clear recommendations are available for mobilization of ischemic stroke patients.

**Objective:**

As early mobilization has been shown to be feasible and safe, we tested the hypothesis that early sitting could be beneficial to stroke patient outcome.

**Methods:**

This prospective multicenter study tested two sitting procedures at the acute phase of ischemic stroke, in a randomized controlled fashion (clinicaltrials.org registration number NCT01573299). Patients were eligible if they were above 18 years of age and showed no sign of massive infarction or any contra-indication for sitting. In the early-sitting group, patients were seated out of bed at the earliest possible time but no later than one calendar day after stroke onset, whereas the progressively-sitting group was first seated out of bed on the third calendar day after stroke onset. Primary outcome measure was the proportion of patients with a modified Rankin score [0–2] at 3 months post stroke. Secondary outcome measures were a.) prevalence of medical complications, b.) length of hospital stay, and c.) tolerance to the procedure.

**Results:**

One hundred sixty seven patients were included in the study, of which 29 were excluded after randomization. Data from 138 patients, 63 in the early-sitting group and 75 in the progressively-sitting group were analyzed. There was no difference regarding outcome of people with stroke, with a proportion of Rankin [0–2] score at 3 months of 76.2% and 77.3% of patients in the early- and progressive-sitting groups, respectively (p = 0.52). There was also no difference between groups for secondary outcome measures, and the procedure was well tolerated in both arms.

**Conclusion:**

Due to a slow enrollment, fewer patients than anticipated were available for analysis. As a result, we can only detect beneficial/detrimental effects of +/- 15% of the early sitting procedure on stroke outcome with a realized 37% power. However, enrollment was sufficient to rule out effect sizes greater than 25% with 80% power, indicating that early sitting is unlikely to have an extreme effect in either direction on stroke outcome. Additionally, we were not able to provide a blinded assessment of the primary outcome. Taking these limitations into account, our results may help guide the development of more effective acute stroke rehabilitation strategies, and the design of future acute stroke trials involving out of bed activities and other mobilization regimens.

**Trial Registration:**

ClinicalTrials.gov NCT01573299

## Introduction

With an estimated 17 million cases worldwide, of which 70% result from an ischemic injury, stroke has a deep socio-economic impact [1]. Patients’ outcomes depend on the initial severity of the cerebral infarction, comorbidities and subsequent medical complications, often due to prolonged immobility [2,3]. In the context of the acute stroke phase, starting out-of-bed mobilization can be a challenging clinical decision to make. Indeed, the inability of the cerebral circulation to adapt to hemodynamic changes, and the dysfunction of the cardiac baroreceptor sensitivity may be expected to limit the use of early upright positioning [4]. Under physiologic conditions, compensatory mechanisms (known as cerebral auto-regulation) prevent the cerebral blood flow (CBF) from varying with systemic blood pressure. During acute stroke, cerebral auto-regulation mechanisms are impaired and any fluctuation in blood pressure can affect the CBF directly [5]. When a change in the position of the body occurs, such as from lying to sitting, a potential drop in the systemic blood pressure could then theoretically translate in a decrease of the CBF. In view of a potential neurological worsening due to a change in the body position, protocols to lead the patient towards an upright position progressively may then be indicated during the acute stroke stage. Clinicians therefore have to weigh potentially beneficial out-of-bed activities in the prevention of complications, against the potential aggravation of neurological deficits, with very little guidance available [6–8].

The hypothesis that early out-of-bed mobilization (sitting or standing within 24h of stroke onset) would improve outcome of people with stroke has first been tested in pilot trials [9,10]. Combined analysis of two pilots studies, AVERT (n = 71 patients) and VERITAS (n = 32 patients), which were respectively conducted in Australia and UK, showed that early out-of-bed mobilization increased the probability for the patient to be independent (modified Rankin score 0–2) at 3 months, and decreased the risk of developing complications during hospitalization [11]. Nevertheless, the recently published international trial AVERT, which enrolled 2104 patients randomized in “usual care” and “very early mobilization” (VEM) arms, the latter with higher frequency and duration of out-of-bed activities, wasn’t able to confirm a more favorable effect of the VEM procedure [12]. Because both groups were mobilized relatively early after stroke onset (median 18.5 vs. 22.4 in VEM and control groups, respectively), the increased frequency (median 6.5 vs. 3 times per day) and duration (median 31 vs. 10 min), may actually serve as stronger discriminators between treatment arms than mobilization onset.

In this study, we explored the hypothesis that upright positioning (out of bed) within 24 hours of stroke onset would be beneficial to patient outcome at 3 months, as compared to a more progressive upright positioning protocol over 3 days, which would minimize acute cerebral hemodynamic changes. To answer this question, we designed a prospective randomized control study in which the two protocols were tested.

## Materials and Methods

### Study design and sample size calculation

The study design was a prospective multicenter, randomized control trial in parallel groups with equal randomization. Patients were enrolled and randomized after screening for inclusion/exclusion criteria and obtaining informed consent. Randomization between early and progressively sitting was performed via numbered sealed envelopes that the investigator would draw from in consecutive fashion (with blocks of 4 in 1:1 ratio, stratified by center) each time a patient was enrolled in the study. The random sequence was generated by our statistician (C.V.) using the SAS software. Data was reported online using a server dedicated to the study.

Sample size was estimated from a previous study in which data from 2 individual trials testing early mobilization within 36h of stroke onset were grouped [11]. Calculation was performed based on a type I error risk of 5% and a power of 80%, in a two-sided approach and with a Fisher exact test. A total of 183 patients per group was calculated as necessary to show a difference of 15% in the prevalence of patients showing a Rankin score [0–2] at three month after stroke onset: 35% in the progressive-sitting group versus 50% in the early-sitting group. Additional risk of low tolerance for early sitting was estimated at 9% (from our own observations) so the sample size has been adjusted to a total of 200 patients per group.

### Protocol approval, registration and patients consent

The SEVEL (Stroke and Early VErticaL positioning) study was approved by the Ethics Committee at the Nantes University Hospital in France (approved September 06^th^ 2011). The authors confirm that all on going and related trials for this intervention are registered. This study was registered at clinicaltrials.org (registration number NCT01573299), with a delay. Indeed it has been registered as a “usual care” study at the level of the Institutional research board, and a miscommunication between our team and the clinical research department caused the delay, which was not sufficiently problematic to force a study restart. Informed and written consent was obtained from all patients.

### Patients, inclusion and exclusion criteria

Patients were recruited from 11 centers located in the North West region of France. Ischemic stroke was diagnosed by a neurologist and defined by the sudden onset of a persistent neurological deficit without sign of bleeding on the CT scan, or MRI. Patients were eligible to participate in the study if they were above 18 year old, exhibited neurological deficits at the inclusion time, were kept in bed (30° maximum) until inclusion time, and if they were enrolled in a healthcare plan (French social security). Patients had to be included at the earliest possible time, and no later than one calendar day after stroke onset. Exclusion criteria were based on 1. stroke severity (malignant infarction, NIHSS > 22, alteration of consciousness with a Glasgow Coma Score < 13), 2. fluctuation of the neurological signs before admission (history of worsening linked to an upright positioning), 3. known intra-cranial stenosis > 50%, symptomatic of the current episode, 4. minor neurological deficit (isolated facial palsy, isolated hemianopia, isolated sensory impairment), 5. iterative vomiting or difficulty in breathing, 6. contra-indication for sitting, e.g. deep vein thrombosis (diagnosed or suspicion) or lower limb fracture, 7. Pre-admission Rankin score [3–6] 8. anticipated difficult follow up (e.g. not speaking French, living in another region), 9. pregnant women, and 10. enrollment in another trial or refusal to participate.

### Intervention

In this study we aimed to test two different protocols for sitting in acute ischemic stroke patients: early and progressive. In the early sitting arm, patients would be seated out of bed at the earliest time possible, but no later that the calendar day after stroke onset. The progressive sitting arm started the day of stroke onset (day 0) when the patient would be positioned in bed at 30°, 45° the day after (day 1) and 60° at day 2 and sitting out of bed at day 3 (which corresponds to the first sitting in this group). Those angles reflect the position of the upper body relative to the bed (and floor). For both protocols, minimal duration of the first sitting was 15 minutes. The procedure could be continued depending on patient fatigue and tolerance (60 minutes maximum). The physiotherapist or the nurses were in charge of collecting the data (blood pressure, tolerance…) related to it. Sitting posture (legs dangling or feet positioned on a foot rest), was done as usual in keeping with each unit’s protocol. The use of a lifter, when necessary, was allowed. Close monitoring of the blood pressure and heart rate was performed: before the sitting procedure, immediately after and 5 minutes after. While sitting, patients showing any sign of low tolerance, defined by neurological worsening (of current or new neurological deficits), vagal reaction (bradycardia or nausea), a greater than 40 mmHg increase of blood pressure topping 180/100mmHg, or a symptomatic decrease in blood pressure, would be put back in bed.

Sitting was repeated on a daily basis according to initial tolerance of the procedure, as approved by the physician in charge. Physiotherapy and deep vein thrombosis prevention by low molecular weight heparin were performed as usual in each unit.

### Outcome measures

Evaluations were made during the intermediate time point at 7 days (or the day of discharge, if before 7 days) and at 3-month after stroke, by a neurologist from the same stroke unit, aware of the study and unblinded to the patient group assignment. The primary outcome measure was the proportion of modified Rankin score [0–2] at three months visit after stroke onset. Patients with major deviation to the protocol or serious adverse event that were enrolled but couldn’t continue the study were assigned a Rankin score in the category [3–6].

Secondary outcomes were assessed during the hospital stay at an intermediate time point at 7 days (or the day of discharge, if before 7 days), and also during the 3-month follow-up. At the intermediate evaluation time point, NIHSS and Rankin scores were evaluated. The Rankin, NIHSS and Barthel scores were provided to the study staff from the NINDS or Internet stroke center websites. Data about the tolerance of the sitting positioning (including prevalence of side effects that forced termination of the procedure) was also collected. A final review of the complications that occurred during hospital stay was also performed at 3 months using a multiple-choice list, and based on both patient interview and medical records. The presence of fatigue (question about the presence or not of an abnormal sensation of being tired, which would impact patient’s activity) was assessed at 3 months only. The duration of sitting out of bed was calculated from the recorded time at which the patient was positioned seated out of bed to the time at which the patient would be put back in bed. The observer would directly write on the case report both first sitting time and sitting duration through specific sections. Length of hospitalization was also recorded for each patient.

### Analyses

Analyses were performed on all data available from patients whose primary outcome was assessed. Continuous variables were presented with mean and standard deviation. Categorical data were expressed as number and percentages (calculated on the number of available data from each group). Primary and secondary outcomes were compared between groups with Chi square test, Student test, Wilcoxon test or generalized linear mixed models (taking into account randomization stratified by center and baseline NIHSS measure for continuous variable). Linear mixed models included baseline NIHSS as fixed effect and center as random effect. Statistical tests were two-sided, and significance has been set at 0.05. Analyses were conducted using the SAS ® 9.3 software.

## Results

**Fig 1:**
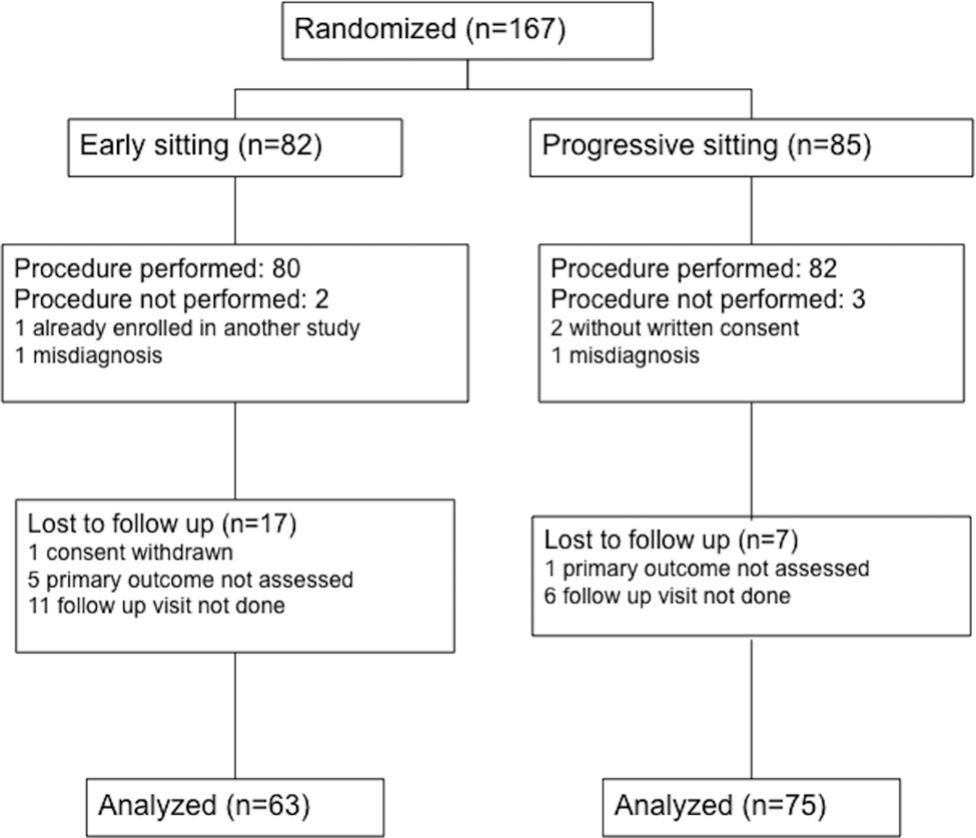
Flow chart of the study.

Enrollment period was conducted between November 2011 and April 2014. The study ended prematurely as it became unviable due to degradation of recruitment rate. One hundred sixty seven patients were enrolled, of whom 29 were excluded (19 in the early sitting group, 10 in the progressive sitting group): for 17 patients the 3 month visit was not performed (6 patients not were not scheduled, 6 patients failed to attend the appointment and for 5 patients no reason was provided), 6 patients lacked evaluation of the Rankin score at 3 months, one patient withdrew his consent and 5 patients subsequently matched exclusion criteria (1 was enrolled in another study, two patients without written consent, two patients misdiagnosed for stroke), Fig 1. One hundred and thirty eight patients were therefore available for analysis. Sixty-three patients were analyzed in the early sitting procedure and 75 in the progressive sitting procedure.

A description of the sample is given in Table 1. The two groups were similar regarding age, gender, stroke etiology and severity. Stroke to the first sitting time was 1.1 ±0.2 days in the early sitting group versus 3 ±0.2 days in the progressive group, reflecting good adherence to protocol for both groups.

**Table 1:** Description of the population.

	Early sitting	Progressive sitting
Analyzed patients	n = 63	n = 75
Age (mean ±SD)	68.1 ±13.7	71.2 ±13.3
Age Median (Q1-Q3)	70.8 [60.9–78.5]	73.6 [62.1–81]
Male (n,%)	48 (76.2)	41 (54.7)
Pre admission Rankin score 0 (n, %)	58 (93.6)	65 (89)
At home before hospitalization (n,%)	62 (98.4)	73 (97.3)
*Cardiovascular risk factors*	56 (88.9)	64 (85.3)
High blood pressure (n,%)	39 (61.9)	48 (64)
Diabetes (n,%)	5 (7.9)	15 (20)
Dyslipidemia (n,%)	31 (49.2)	34 (45.3)
Current or past smoking (n,%)	20 (31.8)	6 (8)
BMI>30 (n,%)	11 (18.3)	12 (17.7)
*Cardiovascular comorbidity*		
Arteritis (n,%)	1 (1.6)	4 (5.3)
Coronaropathy (n,%)	9 (14.3)	10 (13.3)
*Qualifying event*		
Admission NIHSS (mean ±SD)	7.2±3.9	7.8±5.6
Median (Q1-Q3)	7 [4–9]	6 [3–10]
Hemiplegia (n,%)	15 (23.8)	13 (17.3)
Aphasia (n,%)	18 (28.6)	25 (33.3)
Admission Rankin score (n,%)		
Available data	61	73
0	0 (0)	3 (4.11)
1	8 (13.11)	9 (12.33)
2	11 (18.03)	11 (15.07)
3	13 (21.31)	21 (28.77)
4	23 (37.7)	22 (30.14)
5	6 (9.84)	7 (9.59)
Rankin score [0–2] (n,%)	19 (31.1)	23 (31.5)
*Stroke etiology*		
Available data	62	73
Athero-thrombotic (n,%)	16 (25.8)	25 (34.2)
Carotid symptomatic stenosis >50%	3 (4.8)	8 (11)
Cardio embolic (n,%)	22 (35.5)	24 (32.9)
Atrial fibrillation	17 (27.4)	18 (24.7)
Dissection (n,%)	1 (1.16)	1 (1.33)
Lacunar (n,%)	10 (16.1)	5 (6.8)
Cryptogenic (n,%)	12 (19.4)	16 (21.9)
Other (n,%)	1 (1.6)	2 (2.7)
Symptomatic intra cranial stenosis	3 (4.8)	1 (1.3)
*First sitting parameters*		
Calculated time to first sitting (day)		
Available data	51	55
Mean ±SD	1.08 ±0.26	2.97.±0.26
Median [Q1-Q3]	1.08 [0.91–1.24]	2.98 [2.78–3.08]
First sitting duration (min)		
Available data	59	61
Mean ±SD	56.6±41.7	83.7 ± 94.76
Median [Q1-Q3]	55 [30–60]	0 [60–90]

First sitting lasts significantly longer in the progressive group compared to the early group: 83.7 ±94.7 minutes versus 56.6 ±41.7 minutes respectively (p<0.05). Tolerance of the sitting procedure was the same in the early and progressive sitting groups, with a prevalence of side effects of 14.5% and 13.7%, respectively (Table 2). Only one patient showed a worsening of the neurological state (early sitting group). Systolic and diastolic blood pressure, as well as heart rate, did not significantly vary between baseline, acute measurement right after being seated, and 5 minutes later (Table 2). Sitting was continued daily for both groups during hospitalization in 96% of cases.

**Table 2:** Tolerance in early and progressive sitting procedures.

Analyzed patients	Early sitting (n = 63)	Progressive sitting (n = 75)	
*Physiological parameters during first sitting procedure*			
Available data			
Before	n = 59	n = 69	
Right after	n = 58	n = 68	
5 minutes after	n = 59	n = 66	
Systolic blood pressure (mmHg, mean ±SD)			
Before	145.5 ±18.6	141 ±21	
Right after	146.8 ±22.3	142.8 ±23.2	
5 minutes after	145 ±21.7	140.4 ±24	
Diastolic blood pressure (mmHg, mean ±SD)			
Before	82.8 ±15.1	80.6 ±14.2	
Right after	84 ±17.4	83.6 ±14	
5 minutes after	84.2 ±15.3	80.1 ±16.3	
Heart rate (bpm)			
Before	75.6 ±13.9	71.9 ±14.5	
Right after	79.2 ±15.6	76.7 ±17.2	
5 minutes after	77.1 ±14.6	74.7 ±15.8	
*Adverse events*			
Available data	n = 62	n = 73	p
Adverse events, total (n,%)	9 (14.52)	10 (13.7)	0.89
Neurological worsening (n,%)	1 (1.61)	0	
Headache (n,%)	0	1 (1.37)	
Vagal reaction (n,%)	1 (3.22)	2 (2.74)	
Symptomatic hypotension (n,%)	1 (1.61)	1 (1.37)	
Blood pressure increase > 180/100mmHg or more than 40 mmHg from baseline (n,%)	2 (3.23)	2 (2.74)	
Fall (n,%)	1 (1.61)	1 (1.37)	
Other (n,%)	3 (4.84)	3 (4.11)	

While both groups improved over the first week, there was a significant difference in the NIHS scores at one week: 3.7±3.7 NIHSS in the early sitting group versus 2.6±3.7 in the progressive arm (p<0.05, Table 3). Nevertheless, outcome at 3 months was comparable between the two groups, with a prevalence of Rankin [0–2] score of 76.2% in the early sitting group and 77.3% in the progressive sitting group (p = 0.52, Table 3). About the same proportion of patients in both groups were living at home at the 3 month visit (86% in the early sitting group and 91% in the progressively sitting group, p = 0.41). Nine deaths were recorded: 3 in the early sitting group (4.84% of the sample) and 6 in the progressively sitting group (8.33%). For five patients (two in the early sitting group, 3 in the progressive sitting group) the Rankin score was assigned. For two patients, a complication that led to the abortion of the sitting procedure occurred (n = 1 in each group, imputed to the [3–6] class). For two others in the “progressive sitting” group, a deviation to the protocol was noted: one was seated at day one, and one was not monitored properly during the first sitting (both imputed to the [3–6] class). One patient had a Rankin score imputed at 5 at 3 months in the “early sitting” group for a recurrent stroke (two months after the qualifying event). Regarding independence in activities of daily living, there was a slight, but significant difference in the Barthel index at 3 months, as patients included in the early sitting procedure would show a higher (p = 0.05) Barthel index (96.7±8.1) than the progressive group (90.5±22.3). Absolute difference for that parameter is 6.1 with a 95% confidence interval of [0.09–12.11]. Fatigue prevalence at 3 months was not different in the two groups: 43.1% in the early sitting group versus 48.5% in the progressive sitting group, respectively, p = 0.81.

**Table 3:** Outcome of patients in early and progressive sitting procedures.

	Early sitting (n = 63)		Progressive sitting (n = 75)		
		Available data		Available data	P
Day 7 or discharge NIHSS mean (±SD)	3.68±3.71	62	2.64±3.71	72	0.03**
Median [Q1-Q3]	2.5 [1–5]		2 [1–3]		
3 month NIHS score (mean ±SD)	1.75±2.44	57	1.71±2.52	66	0.9**
Median [Q1-Q3]	1 [0–3]		1 [0–2]		
Day 7 or discharge Rankin score (mean ±SD)	2.1±1.5	62	1.75±1.32	72	0.07**
Median [Q1-Q3]	2 [1–4]		1.5 [1–3]		
Day 7 or discharge detailed Rankin score(n,%)		62		72	
0	11 (17.74)		12 (16.67)		
1	13 (20.97)		24(33.33)		
2	15 (24.19)		17 (23.61)		
3	7 (11.29)		10 (13.89)		
4	14 (22.58)		7 (9.72)		
5	2 (3.23)		2 (2.78)		
3 month Rankin [0–2] (n,%)	48 (76.19)	63	58 (77.33)	75	0.52**
3 month detailed Rankin score (n,%)		62		72	
0	19 (30.65)		18 (25)		
1	20 (32.26)		23 (31.94)		
2	9 (14.52)		17 (23.61)		
3	8 (12.9)		4 (5.56)		
4	2 (3.23)		3 (4.17)		
5	1 (1.61)		1 (1.39)		
6	3 (4.84)		6 (8.33)		
3 month Barthel Index (mean ±SD)	96.67±8.09	57	90.53±22.28	66	0.05**
Median [Q1-Q3]	100 [100–100]		100 [95–100]		
Discharge destination (n,%)		58		67	0.27
Transitional care hospital	2 (3.45)		5 (7.46)		
Another hospital	0		3 (4.48)		
Rehabilitation	21 (36.21)		18 (26.87)		
Home	35 (60.34)		41 (61.19)		
Patients living at home at 3 months (n,%)	49 (84.5)	58	60 (90.9)	66	0.41
3 month Fatigue (n,%)	25 (43.1)	58	32 (48.48)	66	0.81**
Days since stroke onset at Day 7 or discharge visit (days, mean ±SD)	6.5±1.51	62	6.78 ±1.13	72	0.27*
Median [Q1-Q3]	7 [6–7]		7 [6–7]		
Days since stroke onset at 3 month visit (days, mean ±SD)	99.95±17.58	58	95.61±11.95	66	0.13*
Median [Q1-Q3]	97.5 [91–107]		95 [90–104]		

* test adjusted on center

**test adjusted on center and baseline NIHSS

No significant difference in the prevalence of medical complications was observed between the early and progressive sitting groups (Table 4). Overall prevalence of medical complications was 28.4%, of which the most frequent were urinary retention (16.4%) followed by constipation (12.7%). Eight percent of our sample showed infectious complication (50% pulmonary and 50% urinary), one patient who had both. Deep vein thrombosis was observed in one case (0.75%). Early sitting didn’t significantly shorten the hospital length of stay, which averaged approximately 10 days in each group (Table 4), or the proportion of patients who were discharged and at home at the time of the visit at 3 months (about 60% in each group).

**Table 4:** Medical complication prevalence in early and progressive sitting procedures.

	Early sitting (n = 63)		Progressive sitting (n = 75)		
		Available data		Available data	P
Length of stay (days, mean ±SD)	9.78±4.85	58	10.53±6.11	66	0.27*
Patient with at least one medical complication during hospitalization (n,%)	15 (24.19)	62	23 (31.94)	72	0.33
Pulmonary infection (n,%)	2 (3.23)	62	4 (5.56)	72	0.69
Urinary tract infection (n,%)	2 (3.23)	62	4 (5.56)	72	0.69
Dysphagia (n,%)	3 (4.84)	62	5 (6.94)	72	0.72
Constipation (n,%)	10 (16.13)	62	7 (9.72)	72	0.27
Urinary retention (n,%)	7 (11.29)	62	15 (20.83)	72	0.14
Deep vein thrombosis (n,%)	1 (1.61)	62	0	72	0.46
Pressure ulcer (n,%)	0	62	0	72	

*test adjusted on center and baseline NIHSS

## Discussion

In this study, we did not observe a significant beneficial or detrimental effect of early sitting, starting as early as possible but no later than the calendar day after stroke onset, compared to a progressive sitting procedure over 3 days post-stroke onset. Our primary endpoint at 3 months was the proportion of each group matching a modified Rankin [0–2]. We reported a significant but slight difference (6.1) in the Barthel index favoring the early sitting group, which may only have border-line clinical relevance. No significant difference was noted regarding medical complications during hospitalization, and tolerance to first sitting was similar in the two procedures.

However, given that the original recruitment goal was set at 200 patients per group, the achieved power to detect a 15% difference between groups was reduced to 37% as opposed the targeted 80% power. As a result, effects of early sitting on recovery, and associated complications, may have been missed. The study is actually sufficiently sensitive to detect a difference of 25% between groups, with a power of 80% and unchanged modified Rankin score proportion of [0–2]. The odds ratio in favor of the intervention “early sitting” effect was calculated at 1.33, with a confidence interval of [0.55–3.19]. We therefore consider the effect of early sitting on stroke outcome, in comparison to the progressive sitting procedure, to be unlikely to be extreme. Also, 17% (n = 29) of the patients that have been randomized were excluded from the study. This does not comply fully with the intention-to-treat principle, but resembles a per protocol analysis. For 23, the primary endpoint of the study was missing (the 3 month visit was not performed, or was performed without this assessment), 5 subsequently matched exclusion criteria and 1 withdrew his consent. We considered these patients to generally match the study population (description of this population is provided as supplementary material).

Rehabilitation strategies at the acute stroke phase (within 24–48 hours) raise significant interest among clinicians. Previously restricted to pilot studies, a major effort by the international AVERT trial reported the results of 2104 patients assigned to an out-of-bed “very early mobilization” (VEM) arm compared to the “usual care” arm. However, VEM was characterized not only by early mobilization starting within 24 hours of stroke onset, but also significantly higher frequency and duration of mobilization. In contrast to the pilot studies, the analysis of the AVERT trial actually revealed a more favorable outcome for patients in the “usual care” arm, as defined by a modified Rankin score [0–2] at 3 months. Because the level of activity during first out-of-bed activities differed greatly between the treatment arms, and may impact outcome and complications independently, the optimal timing of first mobilization still remained to be individually addressed.

In our study, the initial level of activity was set at a minimum of 15 minutes of sitting, and the staff in charge (physicians, nurses or physiotherapists) decided about the total duration of the procedure, according to patient tolerance and comfort. A maximum of 60 minutes for the first sitting was recommended, but not respected in most cases, probably because of the overall good tolerance of the procedure. In the progressive sitting group, a longer first mobilization was performed, but adjusting by this factor did not change the significance of primary outcome at 3 months. We did not record the time spent out of bed in the following days after the day of the first sitting, hence we cannot compare this parameter between groups. However, we did collect information about whether the first-sitting procedure was continued subsequently. In almost all patients, and regardless their group affiliation, the sitting procedure was continued at least once a day afterwards.

For the early sitting group, recommendations were given to sit the patient out of bed at the earliest possible time. Our median time to first mobilization in the early sitting group is 25.9 hours, which is longer than the 22.4h of the usual care group of the AVERT study. Twenty out of the 51 patients (39%) for whom we calculated an exact time to mobilization in the early seating group, were mobilized within 24 hours after stroke onset, all of them in the 12–24 hour interval. These first 24 hours may be critical to stroke expansion. Other strategies will be explored at this stage by the on-going clinical study Headpost, which compares a lying flat position with a 30 degrees in-bed position, within the first 24 hours after stroke onset [13]. In both arms, the duration of first sitting (30–60 IQR) was longer than in the AVERT trial (16.5–50.5 IQR), which could indicate that the detrimental effects of the VEM protocol in the AVERT trial may not stem from the duration of out-of-bed activities. Instead, the frequency of out-of-bed activities may emerge as a possible predictor of less favorable outcomes in the AVERT study: VEM and usual-care groups differed significantly in daily frequency of mobilization (median 6.5 vs. 3 per day). In contrast, our study did not specifically modify frequency of mobilization between early and progressive mobilization groups but deferred to each center’s standard care practice, which was applied equally to both groups. Repeated challenge of the cerebral auto regulation through more frequent upright positioning during the acute stroke phase may explain this observation. Future studies together with further analyses of the AVERT dataset would be needed to characterize and analyze this parameter in isolation.

Medical complication rate was lower than in previously published work about acute stroke [3,11]. Other studies testing early mobilization during acute stroke phase also showed a comparable rate of medical complications between groups mobilized in different fashion [14]. While reflecting a typical hospital-based population, most of our patients showed relatively mild neurological deficits, and thus were less likely to develop medical complications based on previous reports [2,15]. This parameter [16,17] may also explain the comparable length of stay between the two groups in our study. However, it is also possible that stroke exploration tests (e.g. carotid ultrasounds, cardiac echography) have artificially increased the patient stay when the neurological deficit was mild.

Our study was limited by slow recruitment and the loss to follow-up rate (about 10% of the initial cohort), which reflects difficulties inherent to conducting intervention studies in the acute phase of stroke. Even though centers were selected based on the number of people with stroke admission per year, several parameters reduced the recruitment rate: 1.) work load of the physicians, which limited time available to clinical research, 2.) high proportion of emergency room admissions, where high staff turnover may have limited enrollments, and 3.) patients’ perceptions of clinical trials, which led several to refuse participation. Future trials for acute stroke procedures may require dedicated resources for greater pre-trial sensitization and training of the staff of the emergency room, and additional communication with patients to relate information about the clinical trial. Finally, we were not able to implement a blinded evaluation of the primary outcome at 3 months, which may allow for some bias from the physician assessing the Rankin score at follow-up.

Taken together our results indicate that there is no extreme effect of the early sitting procedure in comparison to a progressive sitting procedure in either direction after ischemic stroke. As early mobilization may enable more treatment possibilities in the rehabilitation process, with an earlier start for out-of-bed activities and a shortened hospitalization, future research efforts on this question are warranted. Our study provides more data about the timing of the first out-of bed activity after stroke, it may contribute to future meta analyses, and improve design of future studies in this area.

## Supporting Information

S1 CONSORT Checklist Consort S1. (DOCX)

S1 Protocol SEVEL protocol translated from French. (PDF)

S2 Protocol Original SEVEL protocol (French). (PDF)

S3 Protocol Protocol bibliography. (PDF)

S4 Protocol Information form for the patient (French). (PDF)

S5 Protocol Information form for relatives in case of emergency (French). (PDF)

S6 Protocol Patient consent form. (PDF)

S7 Protocol Consent form for relatives in case of emergency (French). (PDF)

S1 Dataset Description of the 29 excluded patients for missing primary outcome. (DOCX)
