# Supplementary material for: Early Sitting in Ischemic Stroke Patients (SEVEL): A Randomized Controlled Trial
Source: PLoS One. 2016 Mar 29;11(3):e0149466. doi: 10.1371/journal.pone.0149466 (PMC4811411; doi:10.1371/journal.pone.0149466)
Supplement: S1 Protocol — (PDF) [file pone.0149466.s002.pdf]

# SEVEL Study

*(**S**troke and **E**arly **VE**rtica**L** positioning)*

# « Upright positioning at the acute phase of ischemic stroke»

**Record # :** n° 2011-A00430-41
**Ref :** BRD 11/4-C
**Ref CPP (IRB):** 21/11

**Principal Investigator:**
Dr Fanny HERISSON, Assistante chef de clinique,
Clinique Neurologique, HGRL,
Bd Monod Saint Herblain, 44093 NANTES Cedex

**Methodologist/statistician:**
Mme Christelle VOLTEAU,
Biostatistician,
Direction de la Recherche
Département Promotion
5, allée gloriette
44093 Nantes Cedex 01
Christelle.volteau@chu-nantes.fr

**Sponsor:**

CENTRE HOSPITALIER UNIVERSITAIRE DE NANTES

**CHU de Nantes**
Contact : Anne OMNES
Direction de la Recherche
Département Promotion
5, allée de l'île Gloriette
44 093 Nantes cedex 01 (FRANCE)
Contact :

Tel : 02 53 48 28 35
Fax : 02 53 48 28 36

# ABSTRACT

| Study title | **Upright positioning at the acute phase of ischemic stroke** |
|---|---|
| **Key words** | Stroke, Positioning, Autonomy, prognosis |
| **Sponsor** | **CHU DE NANTES (Nantes University hospital)** |
| **PI** | Dr Fanny HERISSON |
| **# of centers involved** | National study on the West region of France:<br>11 centers: Nantes, Vannes, Quimper, Tours, Rennes, Angers, Saint Brieuc, Le Mans, La Roche sur Yon, Saint Nazaire, Chateaubriand. |
| **Study type** | Biomedical research |
| **Study characteristics** | ❖ Total duration: *12 months*<br>❖ Recruitment duration: *9 months*<br>❖ Follow up duration / patient: *3 months* |
| **Design of the study** | ❖ Multicenter (national)<br>❖ Controlled<br>❖ Randomized (non stratified)<br>❖ Open<br>❖ Prospective<br>❖ In parallel groups |
| **Objectives** | **Principal aim:**<br>To show that functional prognosis, at 3 months after ischemic stroke, is better for the patients mobilized out of bed early, compared to the ones progressively mobilized out of bed.<br><br>**Secondary aims:**<br>- To show that neurological deficits (NIHSS) observed at 7 days and 3 months after stroke are improved in the Early out of bed mobilization (EOOBM) arm.<br>- To show that the functional deficit at 7 days is better in the "early" mobilized group.<br>- To show that the level of autonomy is improved at 7 days and 3 months in the "early mobilization" group.<br>- To show that EOOBM increases the chance for the patient to be back home faster<br>- To show that the length of stay is shorter in the "early mobilized" group<br>– To assess the tolerance of the EOOBM in both groups<br>- To show that EOOBM decreases the rate of immobility related mobilization<br>- To assess the impact of the two upright positioning procedures on the post stroke fatigue at 3 months |
| **# of patients required** | 400 patients |
| **Visits and exams schedule** | - Inclusion visit<br>- 7 day visit<br>- 3 month visit |

| | |
|---|---|
| **Inclusion, non-incusion and exclusion criteria** | **Inclusion:**<br>- Age 18 or above<br>- Neurological deficit that occurred the same day or the day before, still present at the inclusion time and caused by a cerebral infarction (sudden neurological deficit without sign of hemorrhage on the CT scan)<br>- Patient hospitalized in the Neurology unit the same day or the day after stroke onset (provided that the patient was positioned not more than 30 degrees of upper body inclinaison)<br>- Health insurance (social security)<br><br>**Exclusion**:<br>- Upfront severe stroke (malignant infarction, not alert with a GCS<13, cerebral herniation on imaging, vital prognosis at stake, NIHSS≥22<br>- Fluctuating neurological deficit (increase/ decrease of the NIHSS of 4 points)<br>- Minor neurological deficit defined by isolated facial palsy (+/- dysarthria), isolated hemianopia, isolated sensitive deficit<br>- Known intra-cranial arterial stenosis >50%, symptomatic of the new symptomatology<br>- History of neurological worsening in association with upright positioning<br>- Situation that may alter the tolerance of early sitting: unstoppable vomiting or difficulty in breathing - Patient non autonomous before hospitalization (defined by a Rankin score>3, can't walk without assistance)<br>- Deep vein thrombosis or suspicion.<br>- Contra indication for early sitting, particularly in case of fracture<br>- Patient of legal aged, with a legal guardian<br>- Anticipated difficulty with the follow up<br>- Participation to another clinical trial<br>- Pregnant or breast feeding women<br>- Patients who can't speak French<br>- Not willing to sign the inform consent form |
| **Treatment, procedure or combinaison of procedures studied** | The patient is seated out of bed the calendar day following the cerebral infarction, or progressively over 72 hours (lying down in bed with the upper body inclined at 30°maximum at D0, 45° at D1, 60° at D2, then seated out of bed at D3) |
| **Reference/usual procedure (if applicable)** | The patient lies down in bed with the upper body inclined at 30° maximum during the acute phase of stroke (Haute Autorite de Sante, May 2009)<br>No existing recommendation (national or international levels) about the duration, and the procedure to follow for the first sitting out of bed for stroke patients |
| **Primary outcome** | Functional prognosis at 3 months evaluated using the modified Rankin score. Proportion of Rankin score [0-2], which correspond to an autonomous life, will be compared between the two groups. |
| **Secondary outcomes** | - Clinical score (neurological deficit) using the NIHSS at 7 days and 3<br>- Functional scale (modified Rankin score) at 7 days<br>- Autonomy scale (Barthel index) at 7 days and 3 months<br>- Percentage of patients back home at 7 days and 3 months<br>- Length of stay<br>- Prevalence of swallowing disturbances, pneumopathy, urinary tract infection, bladder catheterization, deep vein thrombosis during |

| | |
|---|---|
| | hospitalization<br>- Side effects of upright positioning in the two groups= tolerance of early sitting (prevalence of side effects)<br>- Post stroke fatigue prevalence in both groups |
| **Other evaluations** | NA |
| **Statistical analyses** | Primary outcome:<br>Percentage of the patients with a Rankin score [0-2] at 3 months will be compared between the two groups by a Chi square test.<br><br>If the test is not significant, a switch to a non inferiority will be performed. Bilateral confidence interval (CI) of 95% will then be calculated, and the inferior border of the CI will be compared to the non inferiority threshold of -5 %<br><br>A multivariate logistic regression model will be used in a second step to adjust on age and former (before the qualifying event) Rankin score.<br><br>Secondary outcomes:<br>- Clinical score (neurological deficit) based on the NIHSS at 7 days and 3 months: comparison of the mean scores between the two groups using a Student's test.<br>- Functional scale (Rankin score) at 7 days: comparison of the proportions of patients with a score [0-2] by a Chi square test.<br>- Autonomy scale (Barthel index) at 7 days and 3 months: comparison of the proportions of patients with a score < 60 (dependent) between the 2 groups using a Chi square or a Fisher test<br>- Length of stay: comparison of the means between the two groups using a Student's test<br>- Percentage of patients back at home before 7 days or 3 months: Chi square test or Fisher test.<br>- Side effects associated with sitting in the two groups (tolerance of upright positioning in the early versus progressive arms): description of the side effect in the 2 groups and comparison of the most frequent (>5%) by a Chi square test or a Fisher test.<br>- Prevalence of swallowing disturbance, pneumopathy, acute urinary retention, bladder catheterization, deep vein thrombosis during hospitalization: comparison between the two groups using a Chi square test or a Fisher test.<br>- Prevalence of post stroke fatigue: comparison between the two groups using a Chi square test or a Fisher test.<br><br>A multivariate logistic regression model will be used in a second step to adjust on age and former (before the qualifying event) Rankin score. |

| | |
|---|---|
| | |

# *SIGNATURE SHEET*

**SIGNATURE of the person responsible for the research**

| **The person responsible for the research commits to conduct this study under the “usual care” status according to the related legislation** | | |
|---|---|---|
| **Name and function of the individual :**<br><br>Ms OMNES Anne | **Date :** | **Signature :** |

**SIGNATURE of the principal investigator and the co investigators:**

I read this application entirely, and confirm that it encloses all the information required to conduct the study. I commit to undertake this study accordingly.
I am aware that this study is registered under a “usual care” study, as defined by the law (L 1121-1 and R 1121-3 from the “code de la santé publique” (legislation for public health)). Procedures and products that are investigated are used in common practice, but specific supervision and monitoring will be done for this study. I commit to undertake this study, in accordance to:

- The declaration of Helsinki
- International (ICH-E6) and French (“recommendation for good clinical practice for biomedical research for drugs used in humans” – November 24th 2006) legislation and recommendations
- National legislation related to clinical trials
- Legislation from the European union (“Directive Essais Cliniques” [2001/20/CE])

I also commit to make available copies of this application as well as all documents related to the conduction of the trial to all investigators and other members of the research team, so that they can work in accordance with it.

| | **Nom :** | **Date :** | **Signature :** |
|---|---|---|---|
| **Principal investigator** | Dr Fanny HERISSON | | |

# *ABREVIATION LIST*

| | |
|---|---|
| AHA | American Heart Association |
| CPP (IRB) | Comité de Protection des Personnes (Institutional Review Board) |
| CNIL | Commission Nationale de l'Informatique et des Libertés |
| CCTIRS | Comité Consultatif sur le Traitement de l'Information en Matière de Recherche dans le Domaine de la Santé |
| CRF | Case Report Form |
| EOOBM | Early out of bed mobilization |
| eCRF | electronic case report form |
| GCS | Glasgow Coma Score |
| HAS | Haute Autorité de Santé |
| ICH | International Conference on Harmonization |
| ID | Identifier |
| INSERM | Institut National de la Santé et de la Recherche Médicale |
| ITT | Intent to treat |
| PP | Per protocol |

# PLAN

1. JUSTIFICATION OF THE STUDY ..... 10
1.1. BACKGROUND ..... 10
1.2. BENEFITS AND RISKS FOR ENROLLED PATIENTS ..... 11
2. AIMS AND READ-OUTS ..... 14
2.1. PRIMARY AIM AND EVALUATION CRITERIA ..... 14
2.2. SECONDARY AIMS AND EVALUATION CRITERIA ..... 14
3. STUDY DESIGN ..... 15
3.1. GENERAL METHODOLOGY ..... 15
3.2. FLOW CHART ..... 15
4. POPULATION STUDIED ..... 16
4.1. DESCRIPTION OF THE POPULATION ..... 16
4.2. PRE-INCLUSION CRITERIA ..... 16
4.3. INCLUSION CRITERIA ..... 16
4.4. EXCLUSION CRITERIA ..... 16
5. STUDY PROCESS ..... 18
5.1. ANALYSES TOOLS AND PROCEDURES ..... 18
5.2. STUDY SCHEDULE ..... 19
5.3. IDENTIFICATION OF THE DATA COLLECTED OUTSIDE OF THE MEDICAL FILE ..... 21
5.4. CRITERIA FOR WITHOLDING THE PARTICIPATION OF A PATIENT ..... 21
6. DATA MANAGEMENT AND STATISTICS ..... 22
6.1. COLLECTION AND MANAGEMENT OF THE DATA ..... 22
6.2. STATISTICS ..... 23
7. VIGILANCE AND MANAGEMENT OF SIDE EFFECTS ..... 26
7.1. DEFINITIONS ..... 26
7.2. LIST OF THE EXPECTED ADVERSE REACTION ..... 27
7.3. MANAGEMENT OF SERIOUS SUSPECTED UNEXPECTED ADVERSE REACTION ..... 28
7.4. MODALITY AND FOLLOW UP OF THE PATIENTS IN CASE OF SUSAR ..... 29
8. ADMINISTRATIVE AND REGULATORY ASPECTS ..... 30
8.1. ACCESS TO THE DATA AND THE ORIGINAL DOCUMENTS ..... 30
8.2. MONITORING ..... 30
8.3. INSPECTION / AUDIT ..... 30
8.4. ETHICAL CONSIDERATIONS ..... 30
8.5. AMENDMENTS ..... 31
8.6. DECLARATION TO THE AUTHORITIES ..... 31
8.7. FUNDING AND INSURANCE ..... 31
8.8. PUBLICATION ..... 32

# *INTRODUCTION*

In common practice, stroke patients are usually kept in bed with the upper body inclined at 30° maximum during the acute stroke phase. Nevertheless, the duration of this positioning has never been clearly defined in clinical trials. As stroke mostly involves the elderly, immobilization can have adverse effects on the clinical outcome, on the rate of complications during hospitalization, and the return to a normal level of activity. On the other hand, clinicians may be fearful to trigger a potential neurological worsening while using early out of bed mobilization (EOOBM) procedures, particularly if the patient has been diagnosed with a common or internal carotid artery stenosis.

In this study, we hypothesize that early mobilization could improve the outcome of stroke patients, decrease the rate of immobilization related complications, and shorten the length of stay. Therefore we are testing here two out of bed mobilization procedures during the acute phase of stroke: « early » versus « progressive » upright positioning.

# 1. JUSTIFICATION OF THE STUDY

## 1.1. BACKGROUND

**Recommendations from groups of experts regarding upright positioning procedure during the acute phase of stroke**

There is no national or international recommendation based on randomized control trials so far. Therefore, horizontal or 30 degrees positioning is admitted as the common practice. According to the « Haute autorite de sante » report (2009), « a lying down positioning with the head elevated at 30 degrees could contributes in the prevention of cerebral edema ».[1] In the same fashion, strict 0 degree lying down could improve cerebral perfusion initially.[2] Experts from the AHA relate that out of bed activity usually starts as soon as the patient is stable, but don't give any specific recommendation. [3] At the European level, no specific recommendation is proposed either.[4] This lack of guidelines leads to a heterogeneous practice. Another strategy to get the patient out of bed is to wait for the results of the ultrasounds before seating the patient. The drawback of this strategy is that it relies on the ultrasound exam availability, so the patient may stay in bed longer than required.

**EOOBM may decrease the rate of complications linked to prolonged immobility**

Complications associated with immobility are: deep vein thrombosis, pneumopathy, urinary tract infections, constipation and pressure sores.[5-7] In the setting of stroke, a study led in Denmark showed that, over 11757 patients, 2969 (25.3%) presented at least one complication during hospitalization: Urinary tract infections (15.5%), pneumopathy (8%), and constipation (7%).[8]

In this study, early mobilization within the first 24 hours after stroke onset significantly decreased the complication rate. These results were confirmed by several pilot studies comparing the « usual » procedure to early out of bed activity. Indeed, mobilized patients showed less pneumopathy, urinary tract infection and fall.[9]

**A particular situation: acute stroke associated with carotid artery stenosis**

In some patients, neurological deficit could be aggravated during early positioning, particularly in the presence of a significant carotid artery stenosis. Indeed, cerebral auto regulation is impaired at baseline in those patients.[10] In such cases, it is possible that the patient displays neurological deficit in situations that challenge cerebral hemodynamics, like standing from a chair, or a decreased sight under bright light.[11] This can be related to what we may call «hemodynamic » cerebral infarction, even if the pathophysiology of these ischemic cerebral injuries is still a matter of debate: hemodynamic and microembolic hypotheses are discussed. [11, 12] The « post conditioning » concept, which consists of short additional moderate hypoperfusion applied to the ischemic area, could be related to patients with carotid stenosis. These transient periods of hypoperfusion have also been shown beneficial in some studies.[13]

**EOOBM may be beneficial to patient outcome**

EOOBM (first days after stroke), in addition to decreasing the risk of complications related to immobility, doesn't seem deleterious to stroke patients.[14] The results of two phase 2 studies have recently been published in the journal Stroke.[9] This publication combines and

analyses the results of two pilot studies: AVERT (Australia) and VERITAS (UK). VERITAS was a prospective study to explore the feasibility of early out of bed activity after stroke (ischemic and hemorrhagic) and has included 32 patients.[15] The study AVERT was a randomized controlled trial, that enrolled 71 patients (ischemic and hemorrhagic stroke), and focused on feasibility and immobility related complications.[16] In AVERT, early out of bed activity (<24 hours) was compared to an « usual » protocol (not described in detail).
In those two publications, the protocol for the first upright positioning is not precise, and the status of the internal carotid artery (presence/absence of stenosis) is not reported. The meta analysis of the two studies combined showed an improvement of the prognosis at 3 months (Rankin score) in the group that was mobilized early.[9]

**Aim of the study**

In this study, we want to test whether early out of bed mobilization (sitting) after stroke improves patients' prognosis at 3 months, and limits complications related to immobility. Secondary parameters such as length of stay and autonomy levels will also be investigated.

**Perspectives**

- **Improve stroke patients care during the acute phase**
- **Decrease complication rate in this population**
- **Socio economic impact (decrease of economic burden) through a decrease in hospital length of stay and a better recovery**

Bibliography is given at the end of the proposal.

## 1.2. *RISKS AND BENEFITS FOR THE ENROLLED PATIENTS*

### 1.2.1. Benefits

#### *1.2.1.1. <u>At the individual level</u>*

Specific follow up after stroke will be undertaken, especially through a mandatory follow up visit at 3 months post stroke.

#### *1.2.1.2. <u>At the collective level</u>*

This work will predominantly benefit stroke patient in general. Results of this study will have an impact on everyday clinical practice, which would also be more homogeneous. Stroke patient care will be optimized. The socio economic impact of this work could be important if the hypothesis tested is verified, through the diminution of the length of stay, and a better patient outcome. In France, cerebral ischemia involves about 150 000 persons a year.

### 1.2.2. Risks

#### *1.2.2.1. Individual level*

- Risks and physical constrains

This study doesn't imply any physical constrain to the patient. Out of bed positioning is performed routinely in stroke units.

- Risks associated with the disease studied

  - Neurological worsening
  - Seizure
  - Sequellae with limitation of autonomy (sensorial-motor deficit, aphasia, loss of sight, gait disturbance, cognitive impairment)
  - Decrease level of consciousness
  - Coma
  - Death

- Risks associated with the procedure studied

Major side effects could be observed:
  - Neurological worsening defined by aggravation of the existing deficits (motor, aphasia,..) or occurrence of new neurological deficits during or within the first 5 minutes after upright positioning.
  - Fall that can be complicated by fracture

A complete list of the side effects can be found in the vigilance section (see infra).

- Psychological risks and constraints

None identified

- Socio-economic risks

Caused by the disease: incapacity to resume work, to get a loan, and possible increase in the insurance fees.

#### *1.2.2.2. Collective risk*

None identified

### 1.2.3. Risk-benefit ratio

The main risk of the study relies on the possibility of neurological worsening, a side effect that can spontaneously occur during the disease natural history, even without any mobilization of the patient. The physician may be fearful of this outcome, which usually leads to a more prolonged immobility for the patient. It increases the length of stay and the risk of complications (urinary

tract infection, constipation, deep vein thrombosis…), and also delays the ability to return to the former level of autonomy. Data from literature, even though incomplete, don't seem to show that EOOBM is associated with a poor outcome. Risks that are taken in this study regarding neurological worsening are therefore low, for a potential benefit at the population scale, improving patient outcome, but also alleviate the socio economic burden of stroke.

# 2. AIMS AND ENDPOINTS

## *2.1. PRIMARY AIM AND EVALUATION CRITERIA*

### 2.1.1. Primary aim

To show that the functional prognosis of stroke patient at 3 months after stroke onset is better in the « early sitting » group compared to the « progressive sitting » group.

### 2.1.2. Primary outcome

Functional prognosis at 3 months evaluated using the modified Rankin score. Proportions of Rankin score [0-2], which correspond to an autonomous life, will be compared between the two groups.

## *2.2. SECONDARY AIMS AND EVALUATION CRITERIA*

### 2.2.1. Secondary aims

To show that neurological deficits observed at 7 days and 3 months after stroke onset are improved in the « early sitting » group.
To show that the functional prognosis at 7 days after stroke onset is improved in the « early sitting » group.
To show that autonomy is improved at 7 days and 3 months in the « early sitting » group
To show that early sitting facilitates the process of going back home
To show that the length of stay is shorter in the « early sitting » group
To show that early sitting decreases the rate of complications related to immobilization.
To explore the tolerance of upright positioning in the two groups
To explore the prevalence of fatigue at 3 months after stroke in both groups.

### 2.2.2. Secondary outcomes

Clinical score (neurological deficit) using the NIHSS at 7 days and 3
Functional scale (modified Rankin score) at 7 days
Autonomy scale (Barthel score) at 7 days and 3 months
Percentage of patients back home at 7 days and 3 months
Length of stay
Prevalence of swallowing disturbances, pneumopathy, urinary tract infection, bladder catheterization, deep vein thrombosis during hospitalization
Side effects of upright positioning in the two groups= tolerance of early sitting (prevalence of side effects)

# 3. POST STROKE FATIGUE PREVALENCESTUDY DESIGN

## 3.1. *GENERAL METHODOLOGY*

Characteristics of the study:

- **Multi-center** (national)
- Parallel groups
- Controlled
- Randomized (not stratified)
- Open
- Prospective

## 3.2. *FLOW CHART*

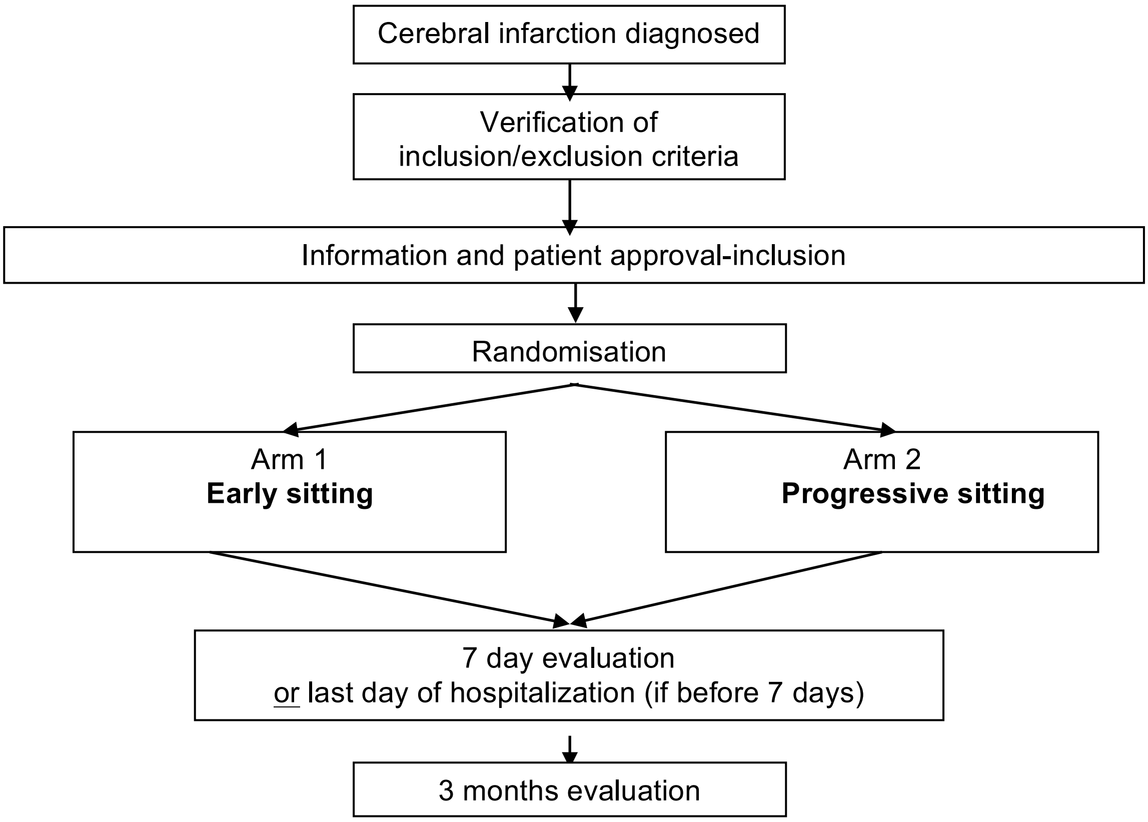

# 4. POPULATION STUDIED

## 4.1. DESCRIPTION OF THE POPULATION

Four hundred patients will be enrolled in this study, equally distributed in two groups (early versus progressive sitting). The targeted population will be of legal age, hospitalized for ischemic stroke in a Neurology unit the same day or the day after stroke onset (directly or through the emergency room or another unit). The study will be proposed and explained in the unit.

The protocol can be started in emergency, so that if the patient shows aphasia, the family can agree on the study after information and explanation (specific letters in annexes 7 and 9) As soon as the clinical state of the patient will be compatible with it, an informed consent will be obtained.

Patients included in the study won't be allowed to participate in another clinical study during the follow up time.

## 4.2. PRE-INCLUSION CRITERIA

NA

## 4.3. INCLUSION CRITERIA

- Aged 18 or above
- Persistent neurological deficit at the time of inclusion, caused by a diagnosed ischemic stroke (defined by a sudden neurological deficit without hemorrhage on the CT.
- Patients hospitalized in a Neurology unit the same day or the day after the neurological deficit (provided that the patient was left with the upper body inclined at 30°maximum up to that time)
- Health coverage

## 4.4. EXCLUSION CRITERIA

- - Upfront severe stroke (malignant infarction, not alert with a GCS<13, cerebral herniation on imaging, vital prognosis at stake, NIHSS≥22
- - Fluctuating neurological deficit (increase/ decrease of the NIHSS of 4 points)
- - Minor neurological deficit defined by isolated facial palsy (+/- dysarthria), isolated hemianopia, isolated sensitive deficit
- - Known intra-cranial arterial stenosis >50%, symptomatic of the new symptomatology

- - History of neurological worsening in association with upright positioning
- - Situation that may alter the tolerance of early sitting: unstoppable vomiting or difficulty in breathing - Patient non autonomous before hospitalization (defined by a Rankin score>3, can't walk without assistance)
- - Deep vein thrombosis or suspicion.
- - Contra indication for early sitting, particularly in case of fracture
- - Patient of legal aged, with a legal guardian
- - Anticipated difficulty with the follow up
- - Participation to another clinical trial
- - Pregnant or breast feeding women
- - Patients who can't speak French
- - Not willing to sign the inform consent form

# 5. STUDY PROCESS

## *5.1. ANALYSES TOOLS AND PROCEDURES*

### 5.1.1. Detailed description of the read outs

Functional scale: modified Rankin score (annex #1)

The modified Rankin score is fast to perform, and gives a general assessment of the functional handicap of the patient, for everyday life. This scale is of common use for clinical trials.[16]

This is a functional scale divided in 7 categories (0 to 6). A 0 score corresponds to an asymptomatic patient, and a score at 6 to a death. Between these extremes, the score varies according to the patient's ability in everyday activity.

Clinical scale : Score NIHSS (annex #2)

This is known and broadly used scale for neurological deficit evaluation, efficient and reproducible.[17]

The NIHSS varies from 0 (no neurological sign) to 43 (the patient is not able to perform any of the tasks - quadriplegic)

Autonomy scale: The Barthel index (annex #3)

The good reproducibility of this index makes it a good tool for clinical trials.[18]

This index assessed the autonomy of the patient, from 10 everyday-life tasks, dealing with self-care and mobility. The score varies from 100 (completely independent) to 0 (completely dependent) ,a core below 60 is associated with dependence in general.

Length of stay

Duration between hospital admission and exit.

### 5.1.2. Description of the techniques used

Early sitting: After an initial stage (the day of stroke onset) of lying with the upper body inclined at 30°maximum, the patient is seated out of bed at the earliest time, for a minimum duration of 15 minutes, under the supervision of the physiotherapist or the nurse. Sitting can be extended up to 60 minutes, if the tolerance is good. Blood pressure and heart rate will be recorded, before, immediately and 5 minutes after upright positioning. Sitting will be repeated everyday according to the same criteria, but the duration will be decided by the staff in charge.

Progressive sitting: After an initial stage (the day of stroke onset, D0) of lying with the upper body inclined at 30°maximum, the patient is seated in bed at 45 degrees (D1), then at 60 degrees (D2). The patient is not seated out of bed before the third day after stroke (D3), for a minimum duration of 15 minutes, under the supervision of the physiotherapist or the nurse (depending of the unit organization). Blood pressure and heart rate will be recorded, before, immediately and 5 minutes after upright positioning. Sitting will be repeated everyday according to the same criteria, but the duration will be decided by the staff in charge.

Supervision during the procedure : Any side effect that led to ceasing the procedure will be noted, for example neurological worsening (degradation of the current state or occurrence of new signs), persistent blood pressure surge (increase of 40mmHg so that the BP rises above 180/100), vagal reaction (bradycardia, nausea, sweat) or hypotension . Close neurological monitoring by the staff is required during the first 5 minutes of the sitting procedure.

Slighter side effects won't lead to the ceasing of the procedure: bearable headache, isolated nausea or vomiting.

## 5.2. STUDY SCHEDULE

Screening: If the patient is eligible, written consent is obtained (from the patient directly or a relative otherwise)

Inclusion visit:

Will be documented:

- Demographic data
  - Date of birth
  - Gender
  - Former autonomy level (Rankin score before stroke onset)
  - Living: location (at home/specialized institution), alone/significant other
- Clinical data
  - Cardio-vascular risk factors
  - History of coronaropathy, arteritis
  - Date and time of the qualifying event
  - Symptoms
  - NIHSS
  - Rankin score
  - Barthel score
- Arm in which the patient is enrolled
- Current treatments (anti-hypertensive drugs, psychoactive drugs, blood thinner)

Early sitting (D1 or D3 according to the group)

- Tolerance, potential side effects
- Blood pressure, heart beat, before, immediately and 5 minutes after upright positioning
- Duration of the upright positioning

According to the group affiliation:

- Early sitting:
  - Duration of the lying down at 30° (D0)
  - Time and duration of the first sitting
  - 
- Progressive sitting:
  - Duration of the lying down at 30° (D0)
  - Time with 45 degrees positioning (D1)
  - Time with 60 degrees positioning (D2)
  - Time and duration of the first sitting (D3)

7 day visit (or before exiting the hospital if before D7)

Will be documented:

- Clinical data

      - NIHSS
      - Rankin score
      - Barthel score

Exit of the hospital:

- Cause of the cerebral infarction, if available
- Internal carotid artery status (ultra sounds)
- Date
- Orientation of the patient (back home, rehabilitation, …)
- Immobility related complications during hospitalization

3 month visit

Will be documented:

- Clinical data
  - NIHSS
  - Rankin score
  - Barthel score
- Cause of the cerebral infarction
- Presence/Absence of post stroke fatigue
- Synthesis about complications that occurred during the hospital stay

## STUDY SCHEDULE

| Actions | D0 (Inclusion) | First sitting | D7 or the last day of hospitalization | M3 |
|---|---|---|---|---|
| **Information of the patient (or relative) / written informed consent patient (or relative)** | X | | | |
| **Medical history** | X | | | |
| **Clinical exam** (Rankin score, NIHSS, Barthel index) | X | | X | X |
| **Documentation**:<br>- Cause of the cerebral infarction<br>- Ultra sound results | | | X | |
| **Monitoring: blood pressure, heart rate, clinical exam** | | X | | |

## 5.3. IDENTIFICATION OF THE DATA COLLECTED OUTSIDE OF THE MEDICAL FILE

- Blood pressure and heart rate during the sitting procedure
- Tolerance of the first sitting, with collection of any related side effect
- Barthel score at inclusion time, D7 (or last day of hospital) and 3 months
- Rankin score at inclusion time, D7 (or last day of hospital) and 3 months

## 5.4. CRITERIA FOR WITHOLDING THE PARTICIPATION OF A PATIENT

### 5.4.1. Precocious stop of a patient participation

Precious stop of a patient participation is defined by:

- Investigator's decision
- Patient's withdrawal of his consent, at any stage of the study. No justification is required. In the particular case of a consent given by a relative (emergency situation), this person has authority to decide to stop the patient's participation to the study, without penalty or prejudice.
- Serious complication not compatible with the continuation of the study
- Patient for whom an unexpected event occurs and is not compatible with the study continuation, as defined in the protocol (death of another cause, occurrence of a clinical situation whose management is not compatible with the continuation of the study).

### 5.4.2. Procedure

For the modality and follow up duration of the patients in case of a precocious stop of the participation in the study, refer to the statistical section.

In case of a precocious stop of a patient's participation to the SEVEL study, the investigator is in charge of collecting as much information as possible regarding the causes and the situation.

### 5.4.3. Criteria to stop the study (outside of statistical considerations)

The study stops 3 months after the inclusion of the last patient. Nevertheless, the study could be stopped earlier is the early sitting is shown deleterious to the patient outcome.

# 6. DATA MANAGEMENT AND STATISTICS

## 6.1. COLLECTION, MANAGEMENT AND SAFETY OF THE DATA

### 6.1.1. Data collection

An electronic case report form (eCRF) will be created by the data management section of the Research department of Nantes university hospital. The software « CAPTURE SYSTEM » developed by CLINSIGHT will be used. All the information required by the protocol must be reported in the eCRF, which will contain the patient's follow up visits. It will enclose all the data in order to confirm that the protocol has been respected, and will be used to detect any issue with the protocol compliance. In the patient electronic file will also be reported all the data required to perform the analyses described in section #7.

A paper version (CRF) of the patient electronic file will be available to facilitate data collection during the visits. This version must then be reported on line.

**Access to the eCRF and to the corresponding database will be secured**. In each center, one person will be responsible for reporting the information in the eCRF. **A coding rule to keep the patient anonymous will be defined before starting using the electronic file.**

The filing of the CRF will be performed by the physician, physiotherapist and/or nurses in charge (in particular for the first sitting supervision).

### 6.1.2. Data coding

The patient coding rule will be as such: patients will be attributed an ID determined by the participation rank in the study for each center. This ID will be automatically calculated during the randomization process described in section # 7.2.8.

By signing up this protocol, the responsible person and all the co-investigators commit to maintain confidential the patient identity. The first letter of the last name, first name, the date of birth together with the patient rank in the study will be the only information noted in the eCRF, and will enable any going back to the CRF if needed afterwards. All the nominative data will be deleted.

### 6.1.3. Data management

Data collection will rely on a clinical database and the creation of masks as in the eCRF and in accordance with the protocol and current legislation. The structure of the database and the eCRF, will be approved by the principal investigator.

## *6.2. STATISTICS*

400 patients are anticipated in the study.

Statistical analyses will be performed by Ms Christelle VOLTEAU, biostatistician – Clinical research department-Nantes university hospital.

### 6.2.1. Description of statistical methods, including any intermediate analysis

All the collected parameters will be described in the 2 groups. Quantitative variables will be described using means, standard deviation, median, minimum and maximum. Qualitative data will be described using number and percentages for each modality.

Primary outcome

Percentage of the patients with a Rankin score [0-2] at 3 months will be compared between the two groups by a Chi square test.

If the test is not significant, a switch to a non inferiority will be performed. Bilateral confidence interval (CI) of 95% will then be calculated, and the inferior border of the CI will be compared to the non inferiority threshold of -5 %

A multivariate logistic regression model will be used in a second step to adjust on age and former (before the qualifying event) Rankin score.

Secondary outcome

- Clinical score (neurological deficit) based on the NIHSS at 7 days and 3 months: comparison of the mean scores between the two groups using a Student's test.

- Functional scale (Rankin score) at 7 days: comparison of the proportions of patients with a score [0-2] by a Chi square test.

- Autonomy scale (Barthel index) at 7 days and 3 months: comparison of the proportions of patients with a score < 60 (dependent) between the 2 groups using a Chi square or a Fisher test

- Length of stay: comparison of the means between the two groups using a Student's test

- Percentage of patient back home before 7 days or 3 months: Chi square test or Fisher test.

- Side effects associated with sitting in the two groups (tolerance of upright positioning in the early versus progressive arms) : description of the side effect in the 2 groups and comparison of the most frequent (>5%) by a Chi square test or a Fisher test.

- Prevalence of swallowing disturbance, pneumopathy, acute urinary retention, bladder catheterization, deep vein thrombosis during hospitalization: comparison between the two groups using a Chi square test or a Fisher test.

- Prevalence of post stroke fatigue: comparison between the two groups using a Chi square test or a Fisher test.

A multivariate logistic regression model will be used in a second step to adjust on age and former (before the qualifying event) Rankin score.

### 6.2.2. Statistical justification for the number of patients required

In a meta-analysis of two pilot studies comparing an « early » to a « usual » mobilization procedure, Craig and coll. (9) showed that the percentage of patients with a Rankin score [0-2] was 34.7 % at 3 months in the « usual » group (n=49) versus 57.4 % in the « early » group (n=54).

In our study, we hypothesized that the functional outcome at 3 months assessed by the Rankin score will be better in the « early » group than in the « progressive » group, with a percentage of Rankin [0-2] of 35% in the « progressive» group and 50% in the « early » group. With an alpha risk of 5% and a power of 80% (bilateral), 183 patients per group are required to show a difference. Total number will then be 366 patients.

Percentage of poor tolerance during the first sitting is estimated between 5 and 10% in the 2 groups. As this may affect the Rankin score at 3 months, the number of patient to include has been increase to 400.

If the superiority of the « early » group is not statistically demonstrated, a switch to non inferiority could be undertaken. For this analysis, the non inferiority threshold is set at -5 % (maximum acceptable clinical difference). With 400 patients, non inferiority will be demonstrated if the real difference between the two groups is between 9 and 15%.

Reference: Committee for Proprietary Medical Products (CPMP). Points to consider on switching between superiority and non inferiority. 2000. European Medical Agencies (AMEA). CPMP/EWP/492/99.

The study will be undertaken in 11 centers. Each one, has the potential to recruit patients, with at least 300 cerebral infarction cases a year.

### 6.2.3. Significance

Significance is set at 5%. If a witch to non inferiority is done, bilateral confidence interval of95% will be built and the inferior border will be compared to the non inferiority threshold set at -5%.

### 6.2.4. Statistical criteria to stop the study

NA.

### 6.2.5. Missing, unused and invalid data

In the situation of a side effect requiring the patient to lie down immediately, or in case of patient death, the Rankin score will be set in the [3-5] interval at 3 month (penalized).

No imputation will be performed on the secondary outcome parameters.

### 6.2.6. Management of the modifications made to the initial plan for analysis

An analysis plan will be written before the freezing of the database and will detail the potential modifications made to the statistical section of the protocol.

### 6.2.7. Choice of the patients to include in the analyses

To analyze for superiority, the population will be analyzed in «intent to treat» (ITT). This population corresponds to all the patients randomized in the study.

Analysis of sensibiility on the population « Per protocol » (PP) will be realized to verify the solidity of the results. This population includes patients who strictly respect inclusion and exclusion criteria, present no protocol deviation and for whom the primary outcome is available. This analysis will exclude deceased patients and the patients with a serious side effect during the first sitting process.

In the non inferiority analyses, analyses in ITT and PP have the same importance. In case of switch to non inferiority, the two analyses will be performed in parallel and the results will be considered accurate if they are the same in the two analyses, in accordance with the recommendations for non inferiority analyses "Points to consider on switching between superiority and non-inferiority"  - Guideline EMEA."

### 6.2.8. Randomization

The randomization list will be created by a statistician from the clinical research department of Nantes university hospital. Randomization will be performed using blocks and a ratio of 1:1.

A sealed envelop that contains the randomization # and the group affiliation will be created for each patient. These envelops will be numbered and stored in each participating center. Each time a patient is included, the investigator will take the next envelop, following the numerical order.

# 7. VIGILANCE AND MANAGEMENT OF SIDE EFFECTS

## 7.1. DEFINITIONS

### 7.1.1. Unexpected event

An unexpected event is defined by any harmful manifestation that occurred to a patient or an individual enrolled in a study, and that is not associated with the method tested.

Any unexpected event encountered during the study, observed by the physician or reported by the patient, will be documented in the patient file in a dedicated section.

The categorization of the unexpected event will be displayed according to this scale :
1 = benign
2 = moderate
3 = severe
4 = vital prognosis at stake

### 7.1.2. Unexpected side effects

A side effect is suspected when an unexpected event that may be caused by the procedure tested, whatever this link is doubtful, plausible, possible or certain.

### 7.1.3. Severe event or side effect

An unexpected event or side effect is qualified as severe if it:

* leads to the patient's death,
* impacts the vital prognosis (for example led to the transfer in intensive care unit)
* triggers an incapacity or an invalidity, either temporary or permanent
* increases the hospital length of stay,
* is responsible of a congenital or neonatal abnormality,
* requires a specific medical care to prevent the degradation of the patient's state to any of the above situation.

### 7.1.4. Expected events or side effects

An expected event or side effect is an event that has been anticipated (and mentioned) in association with the disease studied.

Reminder : Severe expected side effects or events will be declared by the study sponsor to the corresponding authorities.

### 7.1.5. Unexpected side effect

An unexpected side effect is an effect whose nature, severity, frequency or evolution doesn't match the information related to the procedures tested during the study.

Reminder: Unexpected serious side effect will be declared to the competent authorities within 7 or 14 days after the information of the sponsor.

## 7.2. LIST OF THE EXPECTED EVENTS OR SIDE EFFECTS

**Associated wit the procedure tested:**

<u>Common undesired events for the « early » and the « progressive » procedures:</u>

In this case the causation of the event by the procedure is considered if it occurs within the first minutes after it started. Outside of this temporal definition, the undesired event will be associated with the evolution of the disease.

- Nausea/Vomiting
- Headache
- Neurological worsening (aggravation of the existing deficits or occurrence of new ones)
- Blood pressure surge persistent on a second measurement, with a BP increase of at least 40mmHg and above 180/100
- Sensation of passing out (linked to a vagal reaction (bradycardia, nausea, sweating) / Hypotensive / other)
- Fall that can be associated with traumatic brain injury or other orthopedic complications (fracture, luxation, spring)

<u>« Progressive arm »: Complications linked to immobility</u>
- Deep vein thrombosis,
- Pneumopathy
- Urinary tract infection,
- Constipation,
- Sores,
- Swallowing disturbances

<u>*"Early arm"*</u>Neurological worsening (aggravation of the existing deficits or occurrence of new ones)

**Associated with the pathology :**

- Nausea/Vomiting
- Blood pressure surge persistent on a second measurement, with a BP increase of at least 40mmHg and above 180/100
- Sensation of passing out (linked to a vagal reaction (bradycardia, nausea, sweating) / Hypotensive / other)

- Fall that can be associated with traumatic brain injury or other orthopedic complications (fracture, luxation, spring)
- Neurological worsening (aggravation of the existing deficits or occurrence of new ones)
- Seizure
- Alteration of consciousness
- Coma
- Death in direct association with the disease (extension of the infarcted zone, cerebral hemorrhage, cerebral edema, herniation), or indirectly linked to the disease (cardiac failure, lung embolism, sepsis, pneumopathy with hypoxia,…)
-Headache
- Swallowing disturbances
- Pneumopathy
- Deep vein thrombosis

**In association with other comorbidities:**

Comorbidities can alter the clinical state of the patient, notably through ischemic cardiopathy or diabetes, that are frequently encountered in the stroke patients.

**In association with the on going therapies:**

Drugs taken by the patient can also have their own side effects. In this situation the referring document will be the summary of product characteristics available on the AFAASAPS website.

## *7.3. MANAGEMENT OF SEVERE SUSPECTED UNEXPECTED ADVERSE REACTION*

### 7.3.1. Notification of severe events or side events

Any serious undesired event, expected or not, (unless described above) will be notified through a specific form. The investigator will be responsible to give a detailed and precise report. A severe undesired event must be immediately reported (within 24 hours) to the sponsor by fax (Clinical research department, CHU de Nantes Fax 02 53 48 28 36).
After reception, the sponsor will report the event to the authorities. Once a year, a security report will be edited.

Precision :

- Hospitalization is not an unexpected event per se, it is a criteria for seriousness when motivated by a deleterious clinical event. Scheduled hospitalization is not considered as a severe event. Also only hospitalization> 24h will be declared as a severe event.

- Death is a severe event itself and also a criteria for severity, but it is important to precise the cause of the death to interpret the risk/benefit data

- The criteria « vital prognosis at stake » implies the patient transfer to an intensive care unit

### 7.3.2. Independent security committee (ISC)

The ISC is a consultative committee in charge of giving its opinion to the sponsor and the principal investigator regarding the security of the study. Its members are experts in clinical research (pathology and methodology) and are not involved in the study otherwise. They are in function for the study duration and are also committed to respect data confidentiality. The designation of the ISC members is collegial between the PI and the sponsor. The ICS receives the annual report on security and can be activated if a severe event or side effect requires further analysis, or if there any doubt regarding the risk/benefit ratio of the study.

List of the ISC members:
Dr Caroline Arquizan, Toulouse University hspital
Dr Marie Hélène Mahagne, Nice University hospital
Pr Patrick Mismetti, St Etienne University hospital

## *7.4. MODALITY AND FOLLOW UP OF THE PATIENTS IN CASE OF UNEXPECTED EVENT OR SIDE EFFECT*

In case of serious SUSAR that led to the patient exiting the study, the patient will be followed up until resolution of the adverse reaction.

# 8. ADMINISTRATIVE AND REGULATORY ASPECTS

## 8.1. ACCESS TO THE DATA AND THE ORIGINAL DOCUMENTS

The medical data of each patient will only be transmitted to the sponsor or any person approved by it, and eventually to the competent authorities if the confidentiality is warranted.
The sponsor can ask for a direct access to the patient file to verify the compliance to the protocol and data, respecting the confidentiality and within the limits set by the legislation.
Collected data could be treated electronically in compliance with the legislation and the CNIL recommendations (reference MR001).

## 8.2. MONITORING

The monitoring of the study will be performed by the clinical research department. A clinical research assistant will regularly go to each center to verify the quality of the data reported in the patient file.

This protocol has been classified according to the risk level for the patient:
Category B : expected risks close to the usual care ones

Monitoring visits will be organized by appointment with the investigator. Clinical research assistant will consult:
- the patient file,
- the nurse patient file,
- the patient study file (CRF).

## 8.3. INSPECTION / AUDIT

Could be organized if needed.

## 8.4. ETHICAL CONSIDERATIONS

### 8.4.1. Written Informed consent

Each Investigator commits to inform the patient in a clear and accurate way, and ask for an written informed consent (informed consent documents in appendix). He will give the patient an information note and a consent form. The patient can only be included in the study after reading and understanding the information and consent forms, signing and dating the consent form.
The investigator will also sign and date the consent. Those documents will be processed in duplicate so that both the investigator and the patient can have one. The one kept by the investigator will be stored in a specific binder for the study.

This protocol can be used in emergency situation. In particular, when the patient shows aphasia, the information and the consent can be provided by a relative or a person who has a close connection to the patient (in accordance with law L. 1122-2 of the “code de la sante publique”). A specific information and consent form has been made. (appendix # 7 et 9). In case of emergency and if the patient is not able to provide his/her consent, this procedure can be used. The patient will be informed at the earliest possible time, and his written consent will be collected in order to continue his participation. This consent from a relative could be collected through a phone call, and signed within 24 hours. Indeed, this study deals with an emergency situation, and the patient needs to be randomized quickly. In the case of a patient with aphasia, who won’t be able to give an informed consent the inclusion wouldn’t be possible, as the family is usually not present at the time the physician visits the patient. Therefore this may create a bias in the study, as aphasia is observed in about 30% of the stroke patients.
As this is a medical research about a procedure and not a drug, and considering that the study can be easily explained over the phone, the consent may be obtain from the family in that fashion.

If this procedure is using, the investigator must write it down in the patient’s file (consent obtained by calling the relative)

The sponsor commits to submit the study protocol to the ethics commitee. Information about the modality and the nature of the study, and the guaranties to protect the patient enrolled in the study will be transmitted.

## 8.5. AMENDMENTS

Amendments will be addressed by the sponsor for authorization and information to the relevant committee: AFSSAPS and /or au IRB in accordance with the legislation law 2004-806 of august 9th 2004.The modified protocol will be updated in a dated version. If needed, information letter and consent will also be modified.

## 8.6. DECLARATION TO THE AUTHORITIES

Authorization for this protocol will be required from the AFAASAPS.

## 8.7. FUNDING AND INSURANCE

The sponsor is responsible for the funding, and subscribes to insurance policy to cover the civil responsability or any financial consequence of the study.

## 8.8. *PUBLICATION*

A copy of the publication will be sent to Nantes University hospital that will be cited as the sponsor. Authors will be determined according to their involvement in the study. The PI (Dr Fanny Herisson) will decide of the author list.

# *APPENDIX LIST*

Appendix 1 : Rankin score

Appendix 2 : NIHSS

Appendix 3: Barthel index

Appendix 6 : Information sheet for the patient

Appendix 7 : Information sheet in case of emergency

Appendix 8 : Patient's consent form

Appendix 9 : Consent form in case of emergency

# *APPENDIX #1: RANKIN SCALE*

| Score | Symptoms |
|---|---|
| 0 | None |
| 1 | No significant disability despite symptoms ; able to carry out all usual duties and activities |
| 2 | Slight disability ; unable to carry out all previous activities, but able to look after own affairs without assistance |
| 3 | Moderate disability ; requiring some help, but able to walk without assistance |
| 4 | Moderately severe disability ; unable to walk without assistance and unable to attend to own bodily needs without assistance |
| 5 | Severe disability ; bedridden, incontinent and requiring constant nursing care and attention |
| 6 | Dead |

# *APPENDIX #2: NIH STROKE SCALE*

Time of evaluation :

**1a Level of consciousness→**
*0 = alert and responsive*
*1 = not alert, but arousable by minor stimulation to obey, answer, or respond*
*2 = not alert ; requires repeated stimulation to attend, or requires strong or painful stimulation*
*3 = comatose, reponds only with reflex motor or autonomic effects*

**1b – LOC questions→**
*0 = answers both questions correctly*
*1 = answers one question correctly*
*2 = answers neither question correctly*

**1c – LOC commands →**
*0 = performs both tasks correctly*
*1 = performs one task correctly*
*2 = performs neither task correctly*

**2 – Best gaze**
*0 = normal* →
*1 = partial gaze palsy ; gaze is abnormal in one or both eyes, but forced deviation or total gaze paresis is not present*
*2 = Forced deviation, or total gaze paresis not overcome by the oculocephalic maneuver*

**3 – Visual field→**
*0 = normal*
*1 = partial hemianopia*
*2 = complete hemianopia*
*3 = bilateral hemianopia (blind including cortical blindness)*

**4 – Facial palsy→**
*0 = normal movements*
*1 = Minor (flattened nasolabial fold, asymmetry on smiling)*
*2 = Partial paralysis (total or near total paralysis of the lower face)*
*3 = Complete paralysis of one or both sides (absence of facial movement in the upper and lower face)*

**5a – Right motor arm (extend arm at 45° for 10″)** →
*0 = No drift*
*1 = Drift (down before 10s doesn't it the bed)*
*2 = Effort against gravity (drifts down to bed before 10s, but some effort)*
*3 = No effort against gravity*
*4 = No movement*

**5b – Left motor arm (extend arm at 45° for 10″)** →
*0 = No drift*
*1 = Drift (down before 10s doesn't it the bed)*
*2 = Effort against gravity (drifts down to bed before 10s, but some effort)*
*3 = No effort against gravity*
*4 = No movement*

**6a – Right motor leg (extend leg at 30° for 5″)** →
*0 = No drift*
*1 = Drift (down before 5s doesn't it the bed)*
*2 = Effort against gravity (drifts down to bed before 5s, but some effort)*
*3 = No effort against gravity*
*4 = No movement*

**6b – Left motor leg (extend leg at 30° for 5″)** →
*0 = No drift*
*1 = Drift (down before 5s doesn't it the bed)*
*2 = Effort against gravity (drifts down to bed before 5s, but some effort)*
*3 = No effort against gravity*
*4 = No movement*

**7 –Limb ataxia**
*0 = none*
*1 = present in one limb*
*2 = present in two limbs*

**8 – Sensory** →
*0 = normal*
*1 = Mild to moderate sensory loss*
*2 = Anesthesia*

**9 – Best language** →
*0 = normal*
*1 = mild to moderate aphasia (loss of fluency or facility of comprehension)*
*2 = Severe aphasia (all communication is through fragmentary expression, cannot name an object)*
*3 = Global aphasia (mute, loss of comprehension)*

**10 – Dysarthria** →
*0 = normal*
*1 = Mild to moderate (can still be understood)*
*2 = Severe(unintelligible, in the absence of or out of proportion to any dysphasia, or is mute/anarthric)*

**11 – Extinction and inattention (neglect)**→
*0 = no abnormality*
*1 = extinction to bilateral simultaneous stimulation in one of the sensory modalities*
*2 = héminégligence sévère ou extinction dans plusieurs modalités sensorielles ; ne reconnaît pas sa main ou s'oriente profound extinction to more than one modality*

**TOTAL** *(addition of the 15 sub-scores)*

# *APPENDIX #3: BARTHEL INDEX*

| Activity | Score |
|---|---|
| **FEEDING**<br>0 = unable<br>5 = needs help cutting, spreading butter, etc., or requires modified diet<br>10 = independent | _____ |
| **BATHING**<br>0 = dependent<br>5 = independent (or in shower) | _____ |
| **GROOMING**<br>0 = needs to help with personal care<br>5 = independent face/hair/teeth/shaving (implements provided) | _____ |
| **DRESSING**<br>0 = dependent<br>5 = needs help but can do about half unaided<br>10 = independent (including buttons, zips, laces, etc.) | _____ |
| **BOWELS**<br>0 = incontinent (or needs to be given enemas)<br>5 = occasional accident<br>10 = continent | _____ |
| **BLADDER**<br>0 = incontinent, or catheterized and unable to manage alone<br>5 = occasional accident<br>10 = continent | _____ |
| **TOILET USE**<br>0 = dependent<br>5 = needs some help, but can do something alone<br>10 = independent (on and off, dressing, wiping) | _____ |
| **TRANSFERS (BED TO CHAIR AND BACK)**<br>0 = unable, no sitting balance<br>5 = major help (one or two people, physical), can sit<br>10 = minor help (verbal or physical)<br>15 = independent | _____ |
| **MOBILITY (ON LEVEL SURFACES)**<br>0 = immobile or < 50 yards<br>5 = wheelchair independent, including corners, > 50 yards<br>10 = walks with help of one person (verbal or physical) > 50 yards<br>15 = independent (but may use any aid; for example, stick) > 50 yards | _____ |
| **STAIRS**<br>0 = unable<br>5 = needs help (verbal, physical, carrying aid)<br>10 = independent | _____ |
| **TOTAL (0–100):** | _____ |

# *APPENDIX #6: INFORMATION SHEET FOR THE PATIENT*

## Information note related to the research

Title of the trial:

# «SEVEL : Upright positioning at the acute phase of ischemic stroke»

**Sponsor: CHU Nantes n°ID RCB: 2011-A00430-41**

Dear patient.

We are asking for your participation to our study, which scope is the management of upright positioning at the acute phase of a cerebral infarction, pathology for which you are currently hospitalized. The name of the study is:

« SEVEL : Upright positioning at the acute phase of ischemic stroke»

Nantes University Hospital is the sponsor of this study, which means that it is responsible for it and its management.

**What is the goal of that study?**

We are going to study two groups of patients: one will be seated out of bed the day following the cerebral infarction (« early sitting » group) and the other one will be seated the third day after stroke, after a progressive upright positioning. («progressive sitting »).

So far, there is no specific recommendation regarding the first sitting procedure at the acute phase of stroke, and practice varies a lot between the Neurology units.

A lot of physicians chose to keep the patient lying down in bed during the first days after stroke, fearing that early out of bed sitting may aggravate the patient's state. This worsening is possible, even if overall rare and transient in clinical practice (if this happens, you would be put back in bed immediately). In addition, there is no scientific proof that early mobilization is harmful to the patient outcome.

In contrast, prolonged immobilization after stroke can be detrimental to the outcome (the ability to be independent for everyday life actions) and increases the probability of complication.

This study is therefore required to explore what is the best procedure for the first out of bed sitting (early or progressive over 3 days). Several hospitals participate in this trial.

If you agree to enroll in this study, it will be voluntarily. You will be allowed to stop your participation at any time, without penalty or prejudice. In that case you will have to inform the physician in charge. In any case, the quality of care won't be altered.

**What will happen during the trial?**
If you agree to participate in this study, you will be randomly affected to one of the two groups: « early » or « progressive » sitting.

The care that will be provided is otherwise the same as what is usually done.

During that study, two follow up visits are planed:
- a at 7 days after the onset of your stroke (or the day of discharge if earlier than 7 days)
- a visit at 3 months

During those visits, a clinical exam will be performed to assess your recovery after stroke.

Your participation will therefore lasts 3 months. Enrolled patients will not be allowed to participate in another clinical trial until the end of the study.

**What are the potential advantages from this participation?**

Follow up and visit at 3 months.

The benefit of this work will be for the stroke patients (150 000 cases a year in France) in general, and it is very important to assess what is the best acre and have more homogeneous practice.

**What are the potential disadvantages from this participation?**

There is no specific disadvantage in relation to this study. Sitting out of bed is part of the usual patient care, as well as the 3 months visit.

**What will happen if the study is stopped, or at the end of the study (or if you decide to stop your participation)?**

The study can be stopped at any time:

- by the authorities,
- by the sponsor, Nantes university hospital : if a new parameter or information appears, the principal investigator will be informed and he will communicate to you whatever may modify your participation.
- By the principal investigator: for medical reasons related to your case (for example a side effect or a change in your clinical situation), the PI can decide to stop the trial and will inform you.
- By yourself: If you decide to enroll in this study, it is on your own will. You will be able to stop your participation, without penalty or prejudice. In that case you will have to inform the physician in charge.

In any case, the physician- investigator will inform you about what to do next.

In any case, your care will not be affected.

**Will I have specific expenses?**
Your participation won't lead to additional fees or expenses, but only the ones related to the usual care.

**What are the obligations and my rights during that research?**

- **HEALTH INSURANCE**

To participate in this study, you must have health insurance (social security in France).

- **PRIVACY AND PROTECTION**

The staff involved in this study is committed to, and observe professional secrecy, as well as your primary care physician.

- **ACCESS TO YOUR DATA- MANAGEMENT OF THE DATA**

In the setting of this study, your personal data will be treated electronically. This will allow us to analyze the results and achieve our goals.
To do so, your medical file (together with the data related to your way of living), will be transmitted to the sponsor (Nantes University hospital). These data will be identified by a number and your initials.
These data could also be, while respecting the confidentiality, transmitted to the French or foreign authorities. In accordance with the law regarding the treatment of files, personal data and freedom (modified law of January 06th 1978), you are allowed to access and modify your data. You also have the right to oppose the transmission of the data covered by the professional secrecy, and that may be used for this study.
The physician-investigator in charge of your case and who knows your identity, will be required to transmit any of these request so that you can use your rights.
You can also access directly or through any physician of your choice to your entire file, in accordance with the law L 1111-7 of the public health law (Code de la Santé Publique).

- **ACCESS TO THE GLOBAL RESULTS OF THE STUDY**

At the end of the trial, and if you request it, you can be informed by the physician-investigator about the global results of this study (when available).

**Legislation**

**This trial is in accordance with:**

- Laws L. 1121-1 à L. 1126-7 of the public health laws regarding biomedical research.

- Law « Informatique et Libertés »(« Informatics and freedom ») of January 06th 1978 modified.

(The texts are available on this website http://www.legifrance.gouv.fr)

**In accordance with the legislation:**

- Nantes university hospital organizes that trial as a sponsor. An insurance policy that guarantees the sponsor civil responsibility as well as the civil responsibility of the staff has been subscribed to the following company SHAM (contract #135964).

- This study received the agreement of the « Comité de Protection des Personnes » (eq. Internal Review Board) Ouest IV (Nantes) le //2011. This research also received the authorization from the AFSSAPS (“Agence Française de Sécurité Sanitaire des Produits de Santé”), le //2011.

With our regards,

**Dr Fanny HERISSON, principal investigator, together with all the staff of the SEVEL study**

**Important contacts:**

**Dr Fanny HERISSON**

✉ Hôpital Nord Laënnec,
Clinique Neurologique,
Boulevard Jacques Monod Saint Herblain,
44093 NANTES Cedex

☎ 02 40 16 51 99

**Sponsor:**

CHU de Nantes, direction de la recherche

✉ 5 allée de l’Ile Gloriette, 44093 NANTES Cedex 1

☎ 02 53 48 28 35

# *INFORMATION SHEET IN CASE OF EMERGENCY*

## Information note related to the research

Title of the trial:

# «SEVEL: Upright positioning at the acute phase of ischemic stroke»

**Sponsor: CHU Nantes n°ID RCB: 2011-A00430-41**

**Physician investigator :………….**

Because of your relative clinical condition, and facing the emergency of the situation and in accordance with the legislation, we are asking you to provide a consent for your relative (last name, first name) ……..to participate in this study.

We're asking your relative to participate to our study, which scope is the management of upright positioning at the acute phase of a cerebral infarction, pathology for which you are currently hospitalized. The name of the study is:

« SEVEL : Upright positioning at the acute phase of ischemic stroke»

Nantes University Hospital is the sponsor of this study, which means that it is responsible for it and its management.

**How to make a decision?**

- The physician investigator explained the study to you. The information are provided in the document « information sheet ». You are invited to read it carefully before you make a decision.
- If you agree for your relative to participate, you will be asked to sign a consent. This consent will also be signed by the physician. Your signature will confirm that you agree that your relative will be enrolled in the study. Your signature, as well as the one form the physician, is mandatory. Even after you signed the consent, you will still have the right to stop the participation. This will not require any justification, as your relative can't express himself or herself.
- As soon as his or her clinical state will allow it, your relative will be informed by the physician-investigator about the trial, and will ask whether he or she agrees to continue the study. Your relative can also stop his or her participation at that stage.

**What is the goal of that study?**

We are going to study two groups of patients: one will be seated out of bed the day following the cerebral infarction (« early sitting » group) and the other one will be seated the third day after stroke, after a progressive upright positioning. («progressive sitting »).

So far, there is no specific recommendation regarding the first sitting procedure at the acute phase of stroke, and practice varies a lot between the Neurology units.

A lot of physicians chose to keep the patient lying down in bed during the first days after stroke, fearing that early out of bed sitting may aggravate the patient's state. This worsening is possible, even if overall rare and transient in clinical practice (if this happens, you would be put back in bed immediately). In addition, there is no scientific proof that early mobilization is harmful to the patient outcome.

In contrast, prolonged immobilization after stroke can be detrimental to the outcome (the ability to be independent for everyday life actions) and increases the probability of complication.

This study is therefore required to explore what is the best procedure for the first out of bed sitting (early or progressive over 3 days). Several hospitals participate in this trial.

As soon as possible, your relative will be informed about that study. He or she will be allowed to stop the study at any time without penalty or prejudice. In that case he or she will have to inform the physician in charge. In any case the quality of care won't be altered.

**What will happen during the trial?**

If you agree for your relative to participate in this study, he or she will be randomly affected to one of the two groups: « early » or « progressive » sitting. The care that will be provided is otherwise the same as what is usually done.

During that study, two follow up visits are planed:

- a at 7 days after the onset of your stroke (or the day of discharge if earlier than 7 days)
- a visit at 3 months

During those visits, a clinical exam will be performed to assess your recovery after stroke.

The participation will therefore lasts 3 months. Enrolled patients will not be allowed to participate in another clinical trial until the end of the study.

**What are the potential advantages from this participation?**

Follow up and visit at 3 months.

The benefit of this work will be for the stroke patients (150 000 cases a year in France) in general, and it is very important to assess what is the best acre and have more homogeneous practice.

**What are the potential disadvantages from this participation?**

There is no specific disadvantage in relation to this study. Sitting out of bed is part of the usual patient care, as well as the 3 months visit.

**What will happen if the study is stopped, or at the end of the study (or if you decide to stop your participation)?**

The study can be stopped at any time:

- by the authorities,

- by the sponsor, Nantes university hospital : if a new parameter or information appears, the principal investigator will be informed and he will communicate to you whatever may modify your participation.

- By the principal investigator: for medical reasons related to your case (for example a side effect or a change in your clinical situation) , the PI can decide to stop the trial and will inform you.

- By yourself: If you decide to enroll in this study, it is on your own will. You will be able to stop your participation, without penalty or prejudice. In that case you will have to inform the physician in charge.

In any case, the physician- investigator will inform you about what to do next.

In any case, your care will not be affected.

**Will my relative have specific expenses?**

The participation won't lead to additional fees or expenses, but only the ones related to the usual care.

**What are the patient obligations and rights during that research?**

❖ **HEALTH INSURANCE**

To participate in this study, the patient must have health insurance (social security in France).

❖ **PRIVACY AND PROTECTION**

The staff involved in this study is committed to, and observe professional secrecy, as well as your primary care physician.

❖ **ACCESS TO YOUR DATA- MANAGEMENT OF THE DATA**

In the setting of this study, the patient personal data will be treated electronically. This will allow us to analyze the results and achieve our goals.
To do so, his/her medical file (together with the data related to his/her way of living), will be transmitted to the sponsor (Nantes University hospital). These data will be identified by a number and his/her initials.
These data could also be, while respecting the confidentiality, transmitted to the French or international authorities. In accordance with the law regarding the treatment of files, personal data and freedom (modified law of January 06th 1978), the patient is allowed to access and modify his/her data. The patient also has the right to oppose the transmission of the data covered by the professional secrecy, and that may be used for this study.
The physician-investigator in charge of your case and who knows the patient identity, will be required to transmit any of these request so that you can use your rights.
The patient can also access directly or through any physician of his/her choice his/her entire file, in accordance with the law L 1111-7 of the public health law (Code de la Santé Publique).

❖ **ACCESS TO THE GLOBAL RESULTS OF THE STUDY**

At the end of the trial, and if you request it, the patient can be informed by the physician-investigator about the global results of this study (when available).

**Legislation**

**This trial is in accordance with:**

- Laws L. 1121-1 à L. 1126-7 of the public health laws regarding biomedical research.

- Law « Informatique et Libertés »(« Informatics and freedom ») of January 06th 1978 modified.

(The texts are available on this website http://www.legifrance.gouv.fr)

**In accordance with the legislation:**

- Nantes university hospital organizes that trial as a sponsor. An insurance policy that guarantees the sponsor civil responsibility as well as the civil responsibility of the staff has been subscribed to the following company SHAM (contract #135964).
- This study received the agreement of the « Comité de Protection des Personnes » (eq. Internal Review Board) Ouest IV (Nantes) le //2011. This research also received the authorization from the AFSSAPS (Agence Française de Sécurité Sanitaire des Produits de Santé, eq. FDA), le //2011.

Additional information :

Please read this information sheet carefully. At any time you can ask for more information about this study, your and your relative's rights and notify any side effect to the physician-investigator :

Dr………..
Address…….
Telephone……….

With our regards,

**Dr Fanny HERISSON, principal investigator, together with all the staff of the SEVEL study**

**Important contacts:**

**Dr Fanny HERISSON**

🖂 Hôpital Nord Laënnec,
Clinique Neurologique,
Boulevard Jacques Monod Saint Herblain,
44093 NANTES Cedex

☎ 02 40 16 51 99

**Sponsor:**

CHU de Nantes, direction de la recherche

🖂 5 allée de l'Ile Gloriette, 44093 NANTES Cedex 1

☎ 02 53 48 28 35

# *APPENDIX# 8: PATIENT'S CONSENT FORM*

**« SEVEL : Upright positioning at the acute phase of ischemic stroke»**

**Sponsor: CHU Nantes n°ID RCB: n° 2011-A00430-41**

I, Miss, Mrs, Mr *(strikethrough unused ones)* (*first name*, *last name)*………………

Date of birth: …../……/……..

**freely and voluntarily accept to participate in the above mentioned study, organized by Nantes University hospital, sponsor, and coordinated by Dr Fanny Herisson, principal investigator.**

**I acknowledge that:**

- The physician who informed me, and answered clearly to my questions, specified that my participation relies on my own will and that I can withdraw my consent at any time.
- I received an information sheet that described the goal, the methods, the benefits and risks related to the research.
- I will be able to obtain information, during or after the termination of the study, about my health situation.
- I understand the information sheet that I received in order to participate in the study.
- I must have health coverage. I confirm that it's the case.
- I am perfectly aware that I can withdraw my consent at any time for any reason and without any responsibility, but I commit to inform my physician if so. The fact that I would not participate in the study any more will not affect my connection to my physician, or the quality of the care that I am given.
- I can ask my physician for additional information at any time.
- If I want to, I can be informed of the global results of this study when they're available.
- My consent doesn't remove any obligation from the physician and the sponsor of the study, in term of responsibility, and I will keep my rights, as guaranteed by the legislation.
- *I will not be allowed to participate to another biomedical research during my participation.*
- *I accept that the recorded data form this study will be treated electronically by the sponsor. I am aware of my access rights in accordance with the CNIL (modified law of January 06th 1978 regarding informatics, files and freedom- law #39) that I can use at any time though the physician in charge of my case and who knows my identity. I could use my right to rectify or oppose to my electronic data, and in that case would notify the physician in charge of my case, who will inform the sponsor.*
- *I agree that the staff in charge of the follow up of the study has access to my medical record.*

Date :
Signature of the patient:

Signature of the physician, who confirms that he or she explained entirely the goal, the methods and the potential risks related to this study.

Date :
Name and Signature :

**This form is to be made in two originals: one will be kept by the investigator, and the second will be given to the patient who gave his or her consent. In case of duplicate, the original is kept by the investigator and a copy is given to the person who gave the consent.**

# *APPENDIX# 9: CONSENT FORM FOR EMERGENCY SITUATION*

**« SEVEL : Upright positioning at the acute phase of ischemic stroke»**

**Sponsor: CHU Nantes n°ID RCB: n° 2011-A00430-41**

I, Miss, Mrs, Mr *(strikethrough unused ones)* (*first name*, *last name)*……………..

Am signing off as: ☐ a trusted person ☐ a relative ☐ a person close to the patient

**And freely and voluntarily accept to participate in the above mentioned study, organized by Nantes University hospital, sponsor, and coordinated by Dr Fanny Herisson, principal investigator.**

- **I acknowledge that**
- As far his/her clinical state allows it, my relative didn't oppose to his/her participation
- The physician who informed me, and answered clearly to my questions, specified that the participation on my relative relies on my own will and that I, or he/she can withdraw my, his/her consent at any time.
- I have been given an information sheet about that study, which specified its goals, methodology benefits and risks.
- My relative will be asked for his/her consent as soon as his/her clinical state allows it.
  I can be informed, during or after the completion of the study, about the health of my relative.
  I understood that, in order to participate in the study, my relative must have health coverage. I certify that it's the case.
- I am perfectly aware that I can withdraw my consent at any time for any reason and without any responsibility, but I commit to inform my physician if so. The fact that my relative would not participate in the study any more will not affect his/her connection to my physician, or the quality of the care that he/she is given.
- I can ask the physician for additional information at any time.
- If I want to, I can be informed of the global results of this study when they're available
- My consent doesn't remove any obligation from the physician and the sponsor of the study, in term of responsibility, and I and my relative will keep our rights, as guaranteed by the legislation.
- *My relative won't be allowed to participate to another biomedical research during my participation.*
- *I accept that the recorded data form this study will be treated electronically by the sponsor. I am aware of the access rights in accordance with the CNIL (modified law of January 06th 1978 regarding informatics, files and freedom- law #39) that my relative or I can use at any time though the physician in charge of the case and who knows the identity of my relative. I could use my right to rectify or oppose to my electronic data, and in that case would notify the physician in charge of my case, who will inform the sponsor.*
- *I agree that the staff in charge of the follow up of the study has access to the medical records of my relative*

Date :

Signature of the person who represents the patient:

Signature of the physician, who confirms that he or she explained entirely the goal, the methods and the potential risks related to this study.

Date :

Name and Signature :

*At the earliest possible time, the consent of the patient will be sought for, his/her clinical state permitted.*

**This form is to be made in two originals: one will be kept by the investigator, and the second will be given to the patient who gave his or her consent. In case of duplicate, the original is kept by the investigator and a copy is given to the person who gave the consent.**
