# Supplementary material for: Early Sitting in Ischemic Stroke Patients (SEVEL): A Randomized Controlled Trial
Source: PLoS One. 2016 Mar 29;11(3):e0149466. doi: 10.1371/journal.pone.0149466 (PMC4811411; doi:10.1371/journal.pone.0149466)
Supplement: S2 Protocol — (PDF) [file pone.0149466.s003.pdf]

Protocole SEVEL

***(S**troke and **E**arly **VE**rtica**L**isation)*

« Verticalisation des patients à la phase aiguë d'un infarctus cérébral »

N°d'enregistrement : n°2011-A00430-41
Ref : BRD 11/4-C
Ref CPP : 21/11

Investigateur Coordonnateur ou personne qui dirige et surveille la réalisation de la recherche :

Dr Fanny HERISSON, Assistante chef de clinique,
Clinique Neurologique, HGRL,
Bd Monod Saint Herblain, 44093 NANTES Cedex

Méthodologiste :

Mme Christelle VOLTEAU,
Biostatisticienne,
Direction de la Recherche
Département Promotion
5, allée gloriette
44093 Nantes Cedex 01
Christelle.volteau@chu-nantes.fr

Etablissement responsable de la recherche :

CENTRE HOSPITALIER UNIVERSITAIRE DE NANTES

CHU de Nantes
Contact : Anne OMNES
Direction de la Recherche
Département Promotion
5, allée de l'île Gloriette
44 093 Nantes cedex 01 (FRANCE)
Contact :

Tel : 02 53 48 28 35
Fax : 02 53 48 28 36

RESUME

**Titre de l'étude**	**Verticalisation des patients à la phase aiguë d'un infarctus cérébral**
**Mots clés**	Infarctus cérébral, Verticalisation, Autonomie, Pronostic
**Responsable de la recherche**	**CHU DE NANTES**
**Investigateur coordonnateur (si étude multicentrique)**	Dr Fanny HERISSON
**Nombre de centres prévus**	Etude nationale sur l'inter-région Ouest<br>11 centres : Nantes, Vannes, Quimper, Tours, Rennes, Angers, Saint Brieuc, Le Mans, La Roche sur Yon, Saint Nazaire, Chateaubriand.
**Type d'étude**	Recherche biomédicale
**Planning de l'étude**	❖ Durée totale : *12 mois*<br>❖ Période de recrutement : *9 mois*<br>❖ Durée de suivi par patient : *3 mois*
**Design de l'étude**	❖ Etude **multicentrique** (nationale)<br>❖ Etude **contrôlée**<br>❖ Etude **randomisée** (non stratifiée)<br>❖ Etude **ouverte**<br>❖ Etude **prospective**<br>❖ Etude **en groupes parallèles**
**Objectifs de l'étude**	**Objectif principal :**<br>Montrer que le pronostic fonctionnel des patients levés précocement est supérieur à 3 mois par rapport au lever progressif.<br><br>**Objectifs secondaires :**<br>- Montrer que les déficits neurologiques (score NIHSS) observés à J7 et 3 mois sont améliorés dans le protocole de lever précoce<br>- Montrer que le pronostic fonctionnel à 7 jours est amélioré dans le groupe lever précoce<br>- Montrer que le degré d'autonomie est amélioré à 7 jours et 3 mois dans le groupe « lever précoce »<br>- Montrer que le lever précoce favorise un retour à domicile plus rapide<br>- Montrer que la DMS est plus courte dans le groupe « lever précoce »<br>- Evaluer la tolérance de la mise au fauteuil dans les deux groupes<br>- Montrer que le lever précoce diminue la fréquence des complications de décubitus<br>- Evaluer l'impact des procédures de lever sur la fatigue post AVC à 3 mois
**Effectif**	400 patients
**Calendrier des différentes visites et des différents examens**	- Visite d'inclusion<br>- Visite à 7 jours (ou sortie si avant J7)<br>- Visite à 3 mois

**Critères principaux de sélection, d'inclusion, de non-inclusion et d'exclusion**	**<u>Inclusion :</u>**<br>- Age supérieur ou égal à 18 ans<br>- Déficit neurologique datant du jour ou de la veille persistant au moment de l'inclusion en lien avec un infarctus cérébral, défini par un déficit neurologique brutal avec absence de lésion hémorragique au scanner<br>- Patient pris en charge en Neurologie le jour ou le lendemain (à condition qu'il soit resté en décubitus 30° maximum la veille) de la survenue de l'infarctus<br>- Affiliation à la sécurité sociale ou CMU<br><br>**<u>Exclusion</u>** :<br>- Infarctus cérébral grave d'emblée (infarctus cérébral malin, troubles de la vigilance avec GCS < 13, engagement cérébral au scanner, pronostic vital engagé à court terme), NIH≥22<br>- Patient très fluctuant sur le plan clinique (épisodes d'aggravations significatives avec majoration du NIHSS de 4 points alternant avec des épisodes d'amélioration).<br>- Anamnèse témoignant de phénomènes d'aggravation clinique à l'orthostatisme nets<br>- Déficit neurologique mineur défini par la survenue isolée de paralysie faciale associée ou non à une dysarthrie, hémianopsie latérale homonyme, trouble sensitif<br>- Sténose artérielle connue > 50% intra crânienne symptomatique de l'infarctus actuel<br>- Conditions faisant craindre une mauvaise tolérance du lever : Vomissements incoercibles, détresse respiratoire<br>- Patient dépendant AVANT l'hospitalisation (défini par un Rankin score>3)<br>- Thrombose veineuse profonde, ou suspicion.<br>- Contre indication au lever du patient, notamment pour des raisons orthopédiques (fracture du col du fémur par exemple)<br>- Majeurs sous tutelle, sous curatelle<br>- Impossibilité d'assurer le suivi pendant la durée de l'essai<br>- Participation à un autre essai thérapeutique<br>- Femmes enceintes et allaitantes<br>- Patients ne parlant pas la langue française, refusant ou inapte au suivi proposé dans l'étude<br>- Refus du patient
**Traitement, acte, combinaison d'actes à l'étude**	Mise au fauteuil le lendemain de l'infarctus versus lever progressif sur 72h (décubitus 30° à J0, 45° à J1, 60° à J2, puis fa uteuil à J3)
**Procédure de référence (si applicable)**	Décubitus dorsal 30° à la phase initiale (HAS mai 2009)<br>Pas de recommandation existante (nationale ou internationale) sur la durée et le protocole de reverticalisation des patients victimes d'infarctus cérébraux.
**Critère de jugement principal**	Pronostic fonctionnel à 3 mois évalué par une échelle fonctionnelle (Proportion de patients avec Score de Rankin [0-2] dans les deux groupes).
**Critères de jugement secondaires**	- Score clinique (déficit neurologique) évalué par le score NIHSS à 7 jours et 3 mois<br>- Echelle fonctionnelle (Score de Rankin) à 7 jours

	- Echelle d'autonomie (Score de Barthel) à 7 jours et à 3 mois<br>- Pourcentage de patients rentrés à domicile avant 7 jours et 3 mois<br>- Durée moyenne d'hospitalisation<br>- Effets indésirables potentiels de la mise au fauteuil dans les deux groupes = Tolérance de l'orthostatisme précoce versus différé<br>- Prévalence des troubles de la déglutition, pneumopathie, rétention aigue d'urine, pose de sonde urinaire, thrombose veineuse profonde durant l'hospitalisation<br>- Prévalence d'un état de fatigue à 3 mois dans les 2 groupes
**Autres évaluations**	NA
**Analyse statistiques**	<u>Critère principal :</u><br>Le pourcentage de patients avec un score de Rankin compris entre 0 et 2 à 3 mois sera comparé entre les 2 groupes par un test du Chi-2.<br>Un switch vers la non-infériorité sera envisagé en cas de différence non significative.<br>Un ajustement sur l'âge et le score de Rankin antérieur sera effectué dans un second temps (modèle de régression logistique multivarié).<br><br><u>Critères secondaires :</u><br>Des tests de Student, du Chi-2, de Fisher permettront de comparer les différents critères secondaires entre les 2 groupes.<br>Des ajustements sur l'âge et le score de Rankin antérieur seront effectués dans un second temps (modèles de régression linéaire et logistique multivarié).

PAGE DE SIGNATURE

SIGNATURE DU RESPONSABLE DE LA RECHERCHE

Le responsable de la recherche s'engage à réaliser cette étude en soins courants selon toutes les dispositions législatives et réglementaires dont pourrait relever la recherche et selon le protocole.		
**Nom et fonction du représentant signataire :**<br><br>OMNES Anne	**Date :**	**Signature :**

SIGNATURE DES INVESTIGATEURS

J'ai lu l'ensemble des pages du protocole de l'essai clinique dont le CHU de Nantes est le responsable de la recherche. Je confirme qu'il contient toutes les informations nécessaires à la conduite de l'essai. Je m'engage à réaliser l'essai en respectant le protocole et les termes et conditions qui y sont définis.

J'ai connaissance que la présente recherche s'inscrit dans le cadre des recherches en soins courants tels que définis par l'alinéa 2° de l'arti cle L 1121-1 et l'article R 1121-3 du code de la santé publique. Les actes sont pratiqués et les produits sont utilisés de manière habituelle, mais des modalités particulières de surveillance sont prévues au travers de ce protocole.

Je m'engage à réaliser l'essai en respectant :

- les principes de la "Déclaration d'Helsinki",
- les règles et recommandations de bonnes pratiques cliniques internationales (ICH-E6) et française (règles de bonnes pratiques cliniques pour les recherches biomédicales portant sur des médicaments à usage humain - décisions du 24 novembre 2006)
- la législation nationale et la réglementation relative aux essais cliniques
- la conformité avec la Directive Essais Cliniques de l'UE [2001/20/CE]

Je m'engage également à ce que les investigateurs et les autres membres qualifiés de mon équipe aient accès aux copies de ce protocole et des documents relatifs à la conduite de l'essai pour leur permettre de travailler dans le respect des dispositions figurant dans ces documents.

	**Nom :**	**Date :**	**Signature :**
**Investigateur coordonnateur**	Dr Fanny HERISSON		

LISTE DES ABREVIATIONS

ARC	Attaché de Recherche Clinique
AHA	American Heart Association
AVC	Accident Vasculaire Cérébral
BPC	Bonnes Pratiques Cliniques
CPP	Comité de Protection des Personnes
CNIL	Commission Nationale de l'Informatique et des Libertés
CCTIRS	Comité Consultatif sur le Traitement de l'Information en Matière de Recherche dans le Domaine de la Santé
CRF	Case Report Form (cahier d'observation)
DMS	Durée Moyenne de Séjour
EDTSA	Echo-Doppler des Troncs Supra Aortiques
GCS	Score de Glasgow
HAS	Haute Autorité de Santé
ICH	International Conference on Harmonization (Conférence internationale pour l'harmonisation)
IDE	Infirmière Diplômée d'Etat
INSERM	Institut National de la Santé et de la Recherche Médicale
TEC	Technicien d'Etude Clinique
TSA	Troncs supra aortiques

TABLE DES MATIERES

RESUME 2
PAGE DE SIGNATURE 5
LISTE DES ABREVIATIONS 6
TABLE DES MATIERES 7
INTRODUCTION 9
1. JUSTIFICATION DE L'ETUDE 10
1.1. POSITIONNEMENT DE LA RECHERCHE 10
1.2. BENEFICES ET RISQUES POUR LES PERSONNES SE PRETANT A LA RECHERCHE 12
2. OBJECTIFS ET CRITERES DE JUGEMENT 14
2.1. OBJECTIF ET CRITERE D'EVALUATION PRINCIPAL 14
2.2. OBJECTIFS ET CRITERES D'EVALUATION SECONDAIRES 14
3. DESIGN DE LA RECHERCHE 16
3.1. METHODOLOGIE GENERALE DE LA RECHERCHE 16
3.2. SCHEMA DE L'ETUDE 16
4. POPULATION ETUDIEE 17
4.1. DESCRIPTION DE LA POPULATION 17
4.2. CRITERES DE PRE-INCLUSION 17
4.3. CRITERES D'INCLUSION 17
4.4. CRITERES D'EXCLUSION 18
5. DEROULEMENT DE L'ETUDE 19
5.1. TECHNIQUES D'ETUDES ET D'ANALYSES 19
5.2. CALENDRIER DE L'ETUDE 20
5.3. IDENTIFICATION DE TOUTES LES DONNEES SOURCES NE FIGURANT PAS DANS LE DOSSIER MEDICAL 22
5.4. REGLES D'ARRET DE LA PARTICIPATION D'UNE PERSONNE 22
6. DATA MANAGEMENT ET STATISTIQUES 24
6.1. RECUEIL ET TRAITEMENT DES DONNEES DE L'ETUDE 24
6.2. STATISTIQUES 25
7. VIGILANCE ET GESTION DES EVENEMENTS INDESIRABLES 29
7.1. DEFINITIONS 29
7.2. LISTE DES EI ATTENDUS 30
7.3. GESTION DES EVENEMENTS INDESIRABLES 31
7.4. MODALITES ET DUREE DU SUIVI DES PERSONNES SUITE A LA SURVENUE D'EVENEMENTS INDESIRABLES 32
8. ASPECTS ADMINISTRATIFS ET REGLEMENTAIRES 33
8.1. DROIT D'ACCES AUX DONNEES ET DOCUMENTS SOURCE 33
8.2. MONITORING DE L'ESSAI 33
8.3. INSPECTION / AUDIT 33
8.4. CONSIDERATIONS ETHIQUES 33
8.5. AMENDEMENTS AU PROTOCOLE 3534
8.6. DECLARATION AUX AUTORITES COMPETENTES 3534
8.7. FINANCEMENT ET ASSURANCE 35
8.8. REGLES RELATIVES A LA PUBLICATION 35

LISTE DES ANNEXES 36
ANNEXE 1 : SCORE DE RANKIN 1
ANNEXE 2 : SCORE NIHSS 1
ANNEXE 3 : INDEX DE BARTHEL 1

INTRODUCTION

Même s'il est communément admis qu'un patient présentant un infarctus cérébral soit initialement installé en décubitus dorsal à 30°, la durée de ce décubitus n'a jamais été définie par une étude clinique. Touchant préférentiellement une population âgée, l'alitement peut avoir un effet néfaste sur la reprise de l'autonomie et la survenue de complications spécifiques. A contrario, l'aggravation neurologique potentielle lors de la mise au fauteuil est la crainte actuelle du clinicien, notamment en cas de sténose artérielle symptomatique. Notre hypothèse de travail est de montrer que le lever précoce non seulement n'est pas néfaste, mais peut aussi améliorer le pronostic fonctionnel du patient, conduit à moins de complications, et raccourcit la durée d'hospitalisation. Nous testons donc dans ce travail deux protocoles de lever différents : précoce versus progressif.

1. JUSTIFICATION DE L'ETUDE

1.1. POSITIONNEMENT DE LA RECHERCHE

Procédures de lever et recommandations des sociétés savantes

A l'heure actuelle, il n'existe aucune recommandation spécifique nationale ou internationale fondée sur une étude clinique concernant la durée du décubitus initial des patients admis pour infarctus cérébral. Selon le rapport de l'HAS (mai 2009) « un positionnement en décubitus dorsal avec la tête surélevée de 30° peut aussi contribuer à prévenir et/ou à limiter l'oedème cérébral ».[1] De la même façon, le décubitus strict (0°) pourrait améliorer la perfusion cérébrale initialement.[2] Les experts de l'AHA constatent que le patient est communément mis au fauteuil dés que son état est stabilisé, mais n'élaborent pas de recommandation particulière.[3] Enfin, au niveau européen, aucune recommandation spécifique n'est proposée.[4] Cette absence de recommandation aboutit à une hétérogénéité des pratiques entre les centres, allant du lever précoce au repos au lit systématique pendant plusieurs jours. Une autre option est celle d'attendre les résultats du doppler des TSA avant de lever le malade, ce qui peut aboutir à un décubitus prolongé pour peu que l'EDTSA ne soit pas réalisable rapidement, ce qui est le cas de nombreux centres.

Le lever précoce est une procédure bien identifiée pour éviter les complications de décubitus.

Les complications de décubitus regroupent : la thrombose veineuse profonde, la pneumopathie d'inhalation, les infections urinaires, la constipation, les escarres.[5-7]

Dans le cadre de l'AVC, une étude menée au Danemark au sein d'unités neuro-vasculaires a montré que sur 11757 patients, 2969, soit 25,3% avait présenté au moins une complication pendant l'hospitalisation, avec au premier plan les infections urinaires (15,5%), les pneumopathies (8%) et la constipation (7%).[8] Dans ce travail, la mobilisation précoce dans les 24 heures suivant l'AVC diminuait significativement ces complications. Ces résultats sont confortés par d'autres études récemment publiées et comparant le lever précoce à un protocole « usuel », et montrant que les patients mobilisés tôt présentent moins de complication de décubitus, et particulièrement moins de pneumopathie, d'infections urinaire, de chutes à distance (51% versus 35%).[9]

Pourquoi craint-on de lever les patients plus tôt ? Cas particulier de la sténose des TSA extra crânienne.

Certains patients pourraient voir leur état neurologique se dégrader lors de la mise au fauteuil, notamment s'il s'agit d'infarctus cérébral secondaire à une sténose significative intra ou extra-crânienne. En particulier ces patients présentent habituellement des perturbations de l'autorégulation du débit sanguin cérébral.[10] Ceci pourrait conduire à une hypoperfusion symptomatique lors de la mise à l'orthostatisme et la survenue systématique de déficit neurologique dans certaines conditions générales telles que la période post prandiale, se lever d'une chaise ou encore l'apparition d'une cécité monoculaire transitoire à la lumière vive.[11] Ceci semble définir le concept d'infarctus hémodynamique.

Cependant, dans le cadre des sténoses extra crânienne, les données scientifiques actuelles tendent à invalider l'hypothèse d'un mécanisme hémodynamique isolé. En effet, de plus en plus de travaux suggèrent que les infarctus survenant dans le cadre d'une sténose extra crânienne sont au moins autant le fait d'un hypodébit que celui de phénomènes emboliques.[11, 12]

En tout état de cause l'aggravation des patients à l'orthostatisme dans ce cadre est purement spéculative et n'a jamais été clairement démontrée.

Il n'est même pas exclu dans ce contexte que des périodes transitoires d'hypoperfusion cérébrale ne puissent constituer un « post-conditionnement » favorable au pronostic. Le principe de post conditionnement consiste à appliquer au territoire infarci des périodes de non ou d'hypoperfusion, dont il est attendu qu'elles favorisent les mécanismes de réparation.[13]

La mobilisation et le lever précoce des patients n'est pas délétère, et semblent même bénéfiques.

Si la mobilisation précoce (dans les premiers jours) des patients permet de diminuer les complications de décubitus, il ne semble pas qu'elle soit pour autant délétère dans le cadre de l'AVC.[14]

Les résultats de deux essais de phase II concernant la mobilisation précoce ont été synthétisés dans une étude publiée récemment dans Stroke.[9] Cette publication intègre les résultats de l'étude AVERT, australienne et l'étude VERITAS britannique. L'étude VERITAS est une étude prospective de faisabilité sur l'apport de la mobilisation précoce des patients victimes d'AVC ischémique ou hémorragique et porte sur 32 patients.[15] L'étude AVERT est une étude contrôlée randomisée prospective, portant sur 71 patients victime d'un AVC ischémique ou hémorragique, et se concentrant sur les complications de décubitus.[16] Dans cette étude, le lever précoce <24heures était comparé à un protocole « usuel » qui n'est pas spécifiquement décrit.

Dans ces deux travaux les protocoles de verticalisation sont imprécis et la question de la sténose carotidienne n'est pas abordée, ce qui est un facteur limitant et n'apporte pas de réponse à la pratique clinique. Il est toutefois à noter que la métanalyse des deux études montre une amélioration du pronostic fonctionnel à 3 mois dans le groupe « lever précoce », qui se déroulait en pratique dans les 24 heures suivant l'infarctus.[9]

But de l'étude

L'objectif du travail est de démontrer que la mobilisation précoce améliore le pronostic fonctionnel des patients à 3 mois de l'infarctus, et permet de limiter les complications de décubitus. Au-delà de cette question la mobilisation précoce des malades devrait raccourcir la DMS, et améliorer la reprise d'une autonomie.

Perspectives attendues

- **Améliorer la prise en charge des patients au décours d'un infarctus cérébral**
- **Diminuer la prévalence des complications de décubitus dans cette population**
- **Impact socio-économique (diminution des coûts) avec reprise plus rapide de l'autonomie et diminution de la durée d'hospitalisation**

Les références bibliographiques figurent en annexe du document.

1.2. BENEFICES ET RISQUES POUR LES PERSONNES SE PRETANT A LA RECHERCHE

1.2.1. Bénéfices

1.2.1.1. *Bénéfice individuel*

Pour le patient participant à l'étude, une surveillance et un suivi particuliers sont mis en place, avec notamment une visite systématique à 3 mois.

1.2.1.2. *Bénéfice collectif*

Les bénéfices de ce travail sont essentiellement collectifs. Les résultats de cette étude auront une influence sur les pratiques cliniques en les rendant plus homogènes. La prise en charge des patients victimes d'infarctus cérébraux sera optimisée.
Enfin, l'impact socio économique de ce travail pourrait être important, notamment si l'hypothèse testée est confirmée, en diminuant la durée d'hospitalisation et en améliorant le pronostic fonctionnel. L'infarctus cérébral concerne en effet 150000 patients chaque année.

1.2.2. Risques

1.2.2.1. *Risque individuel*

➢ Risques et contraintes physiques

Cette étude ne présente pas de contrainte particulière pour le patient. Le fait d'être installé au fauteuil est un acte pratiqué usuellement, de même que la visite de suivi à 3 mois.

➢ Risques liés à la maladie

- Aggravation neurologique
- Crise comitiale
- Séquelles invalidantes avec limitation de l'autonomie (déficit sensitivomoteur, aphasie, troubles visuels, troubles de l'équilibre, troubles cognitifs)
- Troubles de la vigilance
- Coma
- Décès

➢ Risques liés à la stratégie/acte à l'étude et aux stratégie/actes associés (EI)

EI majeurs pouvant être observés :
- aggravation neurologique définie par la majoration des déficits constatés (majoration d'un déficit moteur, d'une aphasie…) ou l'apparition de nouveaux symptômes neurologiques au décours (pendant et dans les 5 minutes qui suivent) de la manœuvre de lever.
- Chute pouvant être compliquée de traumatisme crânien, fractures de membres, …

La liste exhaustive des EI figure dans la section vigilance (voir infra).

- Risques et contraintes psychologiques

Aucune contrainte psychologique n'est envisagée.

- Risques socio-économiques

Liés à la maladie : impossibilité de reprendre son travail, difficulté d'obtenir un crédit avec majoration de la prime d'assurance au crédit

Liés à la recherche : aucun

1.2.2.2. Risque collectif

Aucun risque collectif n'est identifié.

1.2.3. Balance bénéfices / risques

Le risque principal de l'étude concerne l'aggravation potentielle du déficit neurologique, effet indésirable pouvant survenir dans l'histoire naturelle de la pathologie en dehors de toute mobilisation du patient. La crainte actuelle du clinicien est de provoquer cette aggravation en levant le patient précocement. Cette crainte conduit souvent à un décubitus prolongé, parfois pendant plusieurs jours. Ceci rallonge la durée d'hospitalisation, retarde la reprise d'une autonomie et fait prendre au patient un risque de complication de décubitus (rétention d'urine, constipation, phlébite…)
Les données de la littérature concernant le lever, même si elles sont critiquables, ne semblent pas montrer une telle aggravation clinique des patients particulièrement lors du lever précoce.
Le risque pris dans ce travail en termes d'aggravation clinique parait donc faible, pour un bénéfice à l'échelle collective très important si l'hypothèse testée est validée, en termes de récupération et de pronostic, mais aussi en termes socio économiques.

2. OBJECTIFS ET CRITERES DE JUGEMENT

2.1. OBJECTIF ET CRITERE D'EVALUATION PRINCIPAL

2.1.1. Objectif principal

Montrer que le pronostic fonctionnel des patients est supérieur à 3 mois dans le groupe « lever précoce » par rapport au groupe « lever progressif ».

2.1.2. Critère d'évaluation principal

Pronostic fonctionnel à 3 mois évalué par une échelle fonctionnelle (Score de Rankin). Seront comparées les proportions de Rankin scores [0-2], correspondant à une autonomie dans la vie courante, dans les deux groupes.

2.2. OBJECTIFS ET CRITERES D'EVALUATION SECONDAIRES

2.2.1. Objectifs secondaires

Montrer que les déficits neurologiques observés à J7 et 3 mois sont améliorés dans le groupe lever précoce
Montrer que le pronostic fonctionnel à 7 jours est amélioré dans le groupe lever précoce
Montrer que le degré d'autonomie est amélioré à 7 jours et 3 mois dans le groupe « lever précoce »
Montrer que le lever précoce favorise un retour à domicile plus rapide
Montrer que la DMS est plus courte dans le groupe « lever précoce »
Evaluer la tolérance de la mise au fauteuil dans les deux groupes
Montrer que le lever précoce diminue la fréquence des complications de décubitus.
Evaluer l'impact des procédures de lever sur la prévalence de la fatigue post AVC à 3 mois.

2.2.2. Critères d'évaluation secondaires

Score clinique (déficit neurologique) évalué par le score NIHSS à 7 jours et 3 mois
Echelle fonctionnelle (Score de Rankin) à 7 jours
Echelle d'autonomie (Score de Barthel) à 7 jours et à 3 mois
Pourcentage de patients rentrés à domicile avant 7 jours et 3 mois
Durée moyenne d'hospitalisation
Prévalence des troubles de la déglutition, pneumopathie, rétention aigue d'urine, pose de sonde urinaire, thrombose veineuse profonde durant l'hospitalisation
Effets indésirables potentiels de la mise au fauteuil dans les deux groupes = Tolérance de l'orthostatisme précoce versus différé

Prévalence de la fatigue post AVC

3. DESIGN DE LA RECHERCHE

3.1. METHODOLOGIE GENERALE DE LA RECHERCHE

La recherche présente les caractéristiques suivantes :

- Etude **multicentrique** (nationale)
- Etude **en groupes parallèles**
- Etude **contrôlée**
- Etude **randomisée** (non stratifiée)
- Etude **ouverte**
- Etude **prospective**

3.2. SCHEMA DE L'ETUDE

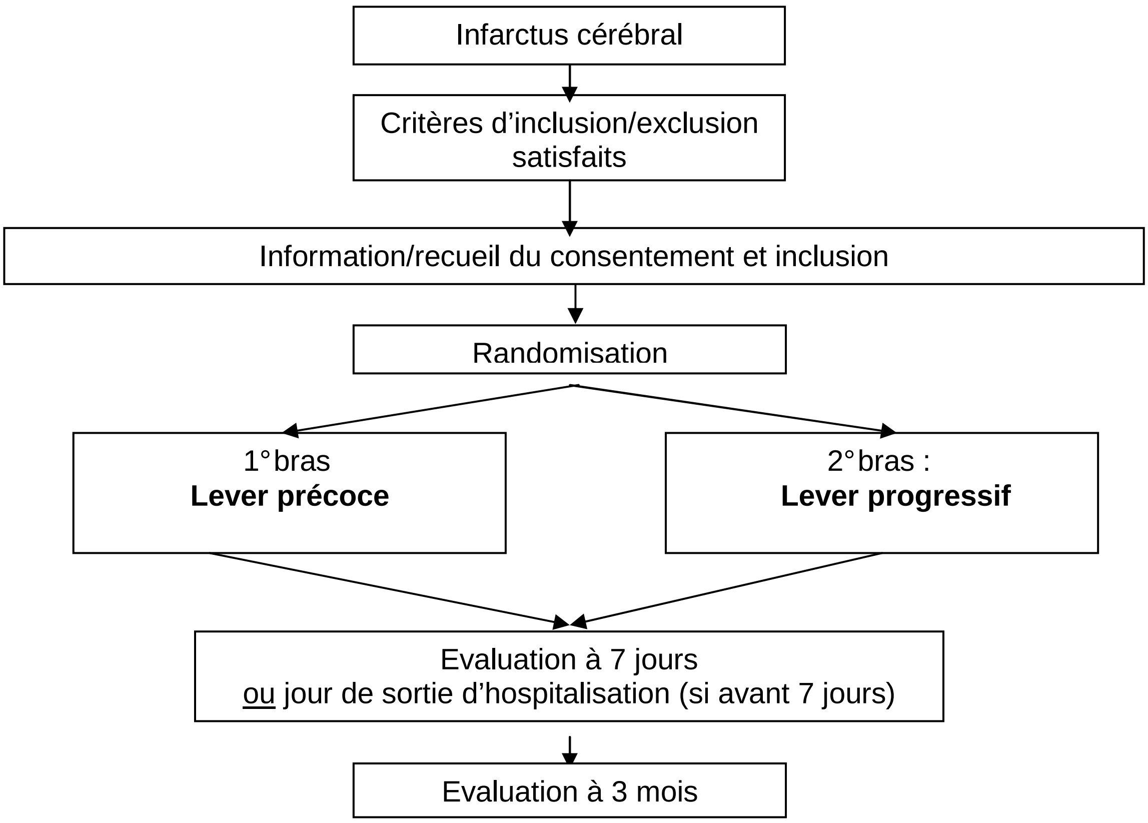

4. POPULATION ETUDIEE

4.1. DESCRIPTION DE LA POPULATION

Quatre cents patients seront inclus dans l'étude, repartis en deux groupes égaux (lever précoce versus progressif). La population cible sera celle de patients majeurs, pris en charge au sein d'un centre hospitalier pour infarctus cérébral, et transféré dans un service de Neurologie le jour ou le lendemain de l'infarctus.
L'étude sera proposée et expliquée à l'admission du patient dans le service de Neurologie (avec remise de la note d'information et signature du formulaire de recueil de consentement patient : annexes 6 et 8 du protocole)

Le protocole peut être mis en œuvre en situation d'urgence, ainsi en cas de trouble phasique du patient, l'information et le recueil du consentement pourra se faire auprès d'une personne de confiance, d'un membre de la famille, ou auprès d'une personne entretenant avec l'intéressé des liens étroits et stables. Une lettre d'information et un consentement sont prévus à cet effet (annexe 7 et 9)
Procédure d'urgence : Ce recueil de consentement réalisé auprès d'une personne de confiance, d'un membre de la famille, ou auprès d'une personne entretenant avec l'intéressé des liens étroits et stables (conformément à l'article L. 1122-2. II du code de la santé publique) pourra ainsi être réalisé dans un premier temps par l'investigateur ou par un médecin qui le représente de manière téléphonique puis dans un deuxième temps par écrit de façon différée et le plus rapidement possible.le recueil de consentement d'un proche pourra se faire de façon téléphonique et signé de façon différée dans les 24 heures (cf. 8.4.1 consentement éclairé écrit)

Dès que son état le permettra, le patient sera informé par le médecin-investigateur sur cette recherche et son consentement lui sera demandé pour la poursuite de la recherche.

Les patients inclus dans l'étude ne pourront pas participer à une autre recherche clinique pendant la durée de l'étude.

4.2. CRITERES DE PRE-INCLUSION

Non applicable

4.3. CRITERES D'INCLUSION

- Age supérieur ou égal à 18 ans
- Déficit neurologique persistant au moment de l'inclusion en lien avec un infarctus cérébral, défini par un déficit neurologique brutal avec absence de lésion hémorragique au scanner, datant du jour ou de la veille
- Patient pris en charge en Neurologie le jour ou le lendemain (à condition qu'il soit resté en décubitus 30° maximum la veille) de la sur venue de l'infarctus
- Affiliation à la sécurité sociale ou CMU

4.4. CRITERES D'EXCLUSION

- Infarctus cérébral grave d'emblée (infarctus cérébral malin, troubles de la vigilance avec GCS < 13, engagement cérébral au scanner, pronostic vital engagé à court terme), NIH≥22
- Patient très fluctuant sur le plan clinique (épisodes d'aggravations significatives avec majoration du NIH de 4 points alternant avec des épisodes d'amélioration).
- Déficit neurologique mineur défini par la survenue isolée de paralysie faciale associée ou non à une dysarthrie, hémianopsie latérale homonyme, trouble sensitif
- Sténose artérielle connue > 50% intra crânienne symptomatique de l'infarctus actuel
- Anamnèse témoignant de phénomènes d'aggravation clinique à l'orthostatisme nets
- Conditions faisant craindre une mauvaise tolérance du lever : Vomissements incoercibles, détresse respiratoire
- Patient dépendant AVANT l'hospitalisation (défini par un Rankin score>3)
- Thrombose veineuse profonde ou suspicion.
- Contre indication au lever du patient, notamment pour des raisons orthopédiques (fracture du col du fémur par exemple)
- Majeurs sous tutelle, sous curatelle
- Impossibilité d'assurer le suivi pendant la durée de l'essai
- Participation à un autre essai thérapeutique
- Femmes enceintes et allaitantes
- Patients ne parlant pas la langue française, refusant ou inapte au suivi proposé dans l'étude
- Refus du patient

5. DEROULEMENT DE L'ETUDE

5.1. TECHNIQUES D'ETUDES ET D'ANALYSES

5.1.1. Description détaillée des paramètres d'évaluation

Echelle fonctionnelle : Score de Rankin (annexe 1)

La réalisation du score de Rankin est rapide et permet d'évaluer de façon globale le handicap fonctionnel d'un patient dans son contexte de vie quotidienne. Cette échelle est couramment utilisée au cours des essais cliniques.[16]

Il s'agit d'une échelle fonctionnelle divisée en 7 catégories, de 0 à 6. Un score de 0 correspond à un patient asymptomatique, le score 6 correspond à un décès. Entre ces extrêmes, le score varie en fonction de la possibilité ou non de pouvoir exécuter des activités de la vie courante.

Echelle clinique : Score NIHSS (annexe 2)

Il s'agit d'une échelle reconnue d'appréciation de la sévérité du déficit neurologique. Elle présente une fiabilité, une reproductibilité et une validité suffisante pour être utilisée dans un essai clinique.[17]

Elle varie de 0 (pas de déficit) à 43 (tous les signes testés sont déficitaires).

Echelle d'autonomie : Index de Barthel (annexe 3)

La bonne reproductibilité de cet index en fait un outil adapté et couramment utilisé dans les essais cliniques.[18]

Cet index permet d'évaluer l'autonomie à partir de 10 aspects fondamentaux de la vie quotidienne concernant les capacités à se prendre en charge et à se mouvoir. L'index varie de 100 (sujet normal) à 0 (dépendance totale, état grabataire), un score inférieur à 60 signe un état de dépendance.

Durée moyenne d'hospitalisation

Entre la date d'admission dans le service la date de sortie d'hospitalisation.

5.1.2. Description des techniques et analyses

Protocole de lever précoce : Après une période initiale (le jour de l'AVC=J0) de décubitus à 30°, le patient est installé le lendema in de l'AVC et le plus précocement possible au fauteuil pendant une durée minimum de 15 minutes, sous surveillance de l'équipe paramédicale (kinésithérapeute ou IDE en fonction de l'organisation des services). En fonction de la tolérance cette mise au fauteuil pourra être prolongée (maximum 60 minutes). La tension artérielle et le pouls devront être renseignés avant le lever, au moment du lever et re-contrôlés à 5 minutes. Le lever sera répété quotidiennement selon la même procédure, les durées pourront être allongées en fonction de la tolérance clinique.

Protocole de lever progressif: Après une période initiale (le jour de l'AVC=J0) de décubitus à 30°, le patient est installé en décubitus à 45 ° le lendemain (J1) de l'AVC, puis 60° le sur lendemain (J2). Il n'est installé au fauteuil qu'à J3, pour une durée minimale de 15 minutes, sous surveillance de l'équipe paramédicale (kinésithérapeute ou IDE en fonction de

l'organisation des services). La tension artérielle et le pouls devront être renseignés avant le lever, au moment du lever, et re-contrôlés à 5 minutes. Le lever sera répété quotidiennement selon la même procédure.

Surveillance clinique lors de l'installation au fauteuil : Seront colligés les événements indésirables potentiels, tels que : aggravation neurologique (majoration des déficits initiaux ou apparition de nouveaux déficits), poussée tensionnelle persistante sur une deuxième mesure (avec majoration de la tension artérielle de 40 mm de Hg et dépassant 180/100) ou malaise lié à une manifestation vagale (bradycardie, nausées, sueurs) ou hypotensif nécessitant de recoucher le patient. Une surveillance clinique neurologique au moins pendant les cinq minutes qui suivent la fin de la procédure est requise.

Les effets indésirables suivants ne nécessitent pas de recoucher le malade : céphalées tolérables, nausées, vomissement isolé

5.2. CALENDRIER DE L'ETUDE

Screening : si éligibilité pour l'étude, recueil du formulaire de consentement (patient ou proche si incapacité)

Visite d'inclusion :

Seront colligés :

- o Données démographiques
  - Date de naissance
  - Sexe
  - Degré d'autonomie antérieur (Rankin score avant l'AVC)
  - Mode de vie : lieu de vie (domicile/institution), seul/accompagnant
- o Données cliniques
  - Facteurs de risque cardiovasculaire
  - Antécédent de coronaropathie, de l'artérite oblitérante des membres inférieurs.
  - Date et horaire de l'infarctus (si renseigné)
  - Symptomatologie initiale
  - Score de NIHSS
  - Score de Rankin
  - Index de Barthel
- o Bras dans le lequel est inclus le patient
- o Classes de Traitements en cours, notamment antihypertenseurs, psychotropes, antiaggrégants plaquettaires, anticoagulants

Mise au fauteuil (J1 ou J3 en fonction du groupe)

- Tolérance, recueil événements indésirables potentiels
- Tension Artérielle, Pouls avant, au moment et à 5 minutes du lever
- Durée de mise au fauteuil

En fonction du groupe de randomisation :

- Respect du protocole de lever précoce :
  - durée en décubitus 30°
  - heure à laquelle le patient est placé en fauteuil à J1/durée

- Respect du protocole de lever progressif :
  - durée en décubitus 30°(J0)
  - heure à laquelle le patient est placé à 45°(J1)/d urée

    - heure à laquelle le patient est placé à 60° (J2)/durée
    - heure à laquelle le patient est placé en fauteuil (J3)/durée

Visite à J7 (ou jour de sortie d'hospitalisation si avant J7)

Seront colligés :

- Données cliniques
    - Score de NIHSS
    - Score de Rankin
    - Index de Barthel

Sortie :

- Bilan étiologique de l'infarctus cérébral, si disponible
- Résultat de l'EDTSA (notamment sténose carotide ou non)
- Date de sortie d'hospitalisation
- Orientation du patient
- Complications de décubitus pendant l'hospitalisation

Visite à 3 mois

Seront colligés :

- Données cliniques
    - Score de NIHSS
    - Score de Rankin
    - Index de Barthel
- Bilan étiologique final de l'infarctus cérébral
- Présence/Absence d'une fatigue secondaire à l'infarctus
- Mise au point sur les complications de décubitus pendant l'hospitalisation, si sortie après J7
- Présence/absence d'un syndrome de fatigue post AVC

CALENDRIER DE L'ETUDE

**Actions**	J0 (Visite d'inclusion)	Lever au fauteuil	J7 ou jour de sortie d'hospitalisation	M3
**Information du patient (proche) / recueil du formulaire de consentement patient (ou proche)**	X			
**Antécédents**	X			
**Examen clinique** (score de Rankin, score de NIHSS, index de Barthel)	X		X	X
**Recueil données** :<br>- Orientation étiologique de l'infarctus<br>- Données de l'EDTSA			X	
**Surveillance clinique paramédicale: tension artérielle, pouls, examen clinique**		X		

5.3. IDENTIFICATION DE TOUTES LES DONNEES SOURCES NE FIGURANT PAS DANS LE DOSSIER MEDICAL

- Tension artérielle et pouls au décours du lever
- Tolérance du lever avec recueil des événements indésirables potentiels
- Index de Barthel à l'inclusion, J7 (ou sortie) et 3 mois
- Score de Rankin à l'inclusion, J7 (ou sortie) et 3 mois.

5.4. REGLES D'ARRET DE LA PARTICIPATION D'UNE PERSONNE

5.4.1. Critères d'arrêt prématuré de la participation d'une personne à la recherche

L'arrêt de participation à l'étude est défini par :

- ❖ Décision de l'investigateur.
- ❖ Patient qui exprime la volonté de retirer son consentement à l'étude à quel que stade que ce soit et ce sans avoir à justifier sa décision

Dans le cas ou le consentement a été récupéré auprès d'un proche (situation d'urgence), celui-ci pourra, tant que le patient sera incapable d'exprimer son consentement ou son opposition, décider d'arrêter la participation de son proche sans pénalités, ni préjudice.

- ❖ Patient pour lequel une complication grave survient, ne permettant plus la poursuite de l'étude
- ❖ Patient pour lequel un événement intercurrent interdit la poursuite de sa participation à l'étude dans les conditions définies par le protocole (décès d'une autre cause, survenue d'une affection intercurrente grave dont la prise en charge entre en concurrence avec les modalités du protocole).

5.4.2. Procédures d'arrêt prématuré de la participation d'une personne à la recherche

Pour les modalités et la durée du suivi des personnes ayant arrêté prématurément l'étude, se reporter à la section statistique.
En cas d arrêt prématuré de la participation d'un patient au protocole SEVEL, l'investigateur s'assurera de colliger le maximum d informations sur le motif de l arrêt et ses circonstances.

5.4.3. Critères d'arrêt de la recherche (hors considérations biostatistiques)

La recherche se termine 3 mois après l'inclusion du dernier patient dans l'étude. Néanmoins, la recherche pourra être arrêtée prématurément si le protocole testé (lever précoce) se montre délétère.

6. DATA MANAGEMENT ET STATISTIQUES

6.1. RECUEIL ET TRAITEMENT DES DONNEES DE L'ETUDE

6.1.1. Recueil des données

Un cahier d'observation électronique (eCRF) sera créé par le Data-management de la Direction de la Recherche du CHU de Nantes. Le logiciel CAPTURE SYSTEM développé par CLINSIGHT est la solution informatique retenue. Toutes les informations requises par le protocole doivent être fournies dans le eCRF. Il reprendra les différentes étapes de la prise en charge du patient dans le protocole. Il doit comprendre les données nécessaires pour confirmer le respect du protocole, déceler les écarts majeurs au protocole et toutes les données nécessaires aux analyses définies en 7.

Une version papier du CRF sera disponible pour les équipes afin que les informations médicales soient remplies lors des visites. Cette version papier sera ensuite remplie en ligne lors des différentes visites.

L'accès à l'eCRF ainsi qu'à la base de données correspondante sera sécurisé. L'établissement de rattachement désignera des personnes responsables de la saisie des données dans le eCRF. Une règle de codage des patients sera définie préalablement à l'utilisation du eCRF.

Le remplissage des CRF se fera à la fois par les médecins et le personnel paramédical (kinésithérapeute, IDE) pour la surveillance du lever.

6.1.2. Codage des données

La règle de codage des patients sera la suivante : les patients recevront un identifiant déterminé par leur ordre d'inclusion dans l'étude. Cet identifiant sera automatiquement calculé lors de la randomisation décrite à la section 7.2.8.

En signant ce protocole la personne responsable et l'ensemble des co-investigateurs s'engagent à maintenir confidentielles les identités des patients qui participent à l'étude. La première lettre du nom, la première lettre du prénom, la date de naissance ainsi que le numéro du patient dans l'étude seront les seules informations qui figureront sur le cahier d'observation (CRF) et qui permettront de rattacher à posteriori le CRF au patient. Toutes les données nominatives seront effacées.

6.1.3. Traitement des données

La collecte des données cliniques reposera sur la mise en place d'une base de données clinique et la création de masques de saisie à l'image du cahier d'observation en conformité avec le protocole et les réglementations actuellement en vigueur.

La structure de la base de données et des écrans de saisie sera approuvée par le responsable de la recherche de l'essai.

6.2. STATISTIQUES

400 patients sont prévus dans l'étude.

Les analyses statistiques seront réalisées par Christelle VOLTEAU, biostatisticienne – Département Promotion de la Recherche – CHU de Nantes.

6.2.1. Description des méthodes statistiques prévues, y compris du calendrier des analyses intermédiaires prévues

L'ensemble des variables recueillies sera décrite dans les 2 groupes. Les variables quantitatives seront décrites avec des moyennes, écarts-type, médiane, minimum et maximum. Les variables qualitatives seront décrites avec les effectifs et pourcentages de chaque modalité.

Critère principal

Le pourcentage de patients avec un score de Rankin compris entre 0 et 2 à 3 mois sera comparé entre les 2 groupes par un test du Chi-2.

Si le test est non significatif, un switch vers un test de non-infériorité sera envisagé. L'intervalle de confiance bilatéral à 95 % de la différence de pourcentage sera alors calculé et la borne inférieure de l'intervalle de confiance sera comparée au seuil de non infériorité définie à -5 %

Dans un second temps, un modèle de régression logistique multivarié permettra d'ajuster la comparaison sur l'âge et le score de Rankin antérieur.

Critères secondaires

- Score clinique (déficit neurologique) évalué par le score NIHSS à 7 jours et 3 mois : comparaison des scores moyens entre les 2 groupes par des tests de Student.

- Echelle fonctionnelle (Score de Rankin) à 7 jours : comparaison par un test du Chi-2 du pourcentage de patients ayant un score compris entre 0 et 2.

- Echelle d'autonomie (Score de Barthel) à 7 jours et à 3 mois : comparaison des pourcentages de patients avec un score < 60 (dépendant) entre les 2 groupes par des tests du Chi-2 ou de fisher.

- Durée moyenne d'hospitalisation : comparaison de la durée moyenne d'hospitalisation entre les 2 groupes par un test de Student.

- Pourcentage de patients rentrés à domicile avant 7 jours et 3 mois : comparaison entre les 2 groupes par des tests du Chi-2 ou de Fisher.

- Effets indésirables potentiels de la mise au fauteuil dans les deux groupes (Tolérance de l'orthostatisme précoce versus différé) : description des effets indésirables survenus dans les 2 groupes et comparaison des EI les plus fréquents par des tests du Chi-2 ou de Fisher (fréquence > 5 %).

- Prévalence des troubles de la déglutition, pneumopathie, rétention aigue d'urine, pose de sonde urinaire, thrombose veineuse profonde durant l'hospitalisation : comparaison entre les 2 groupes par des tests de Chi-2 ou de Fisher.

- Prévalence de la fatigue post AVC : comparaison entre les 2 groupes par des tests de Chi-2 ou de Fisher.

Dans un second temps, des modèles de régression linéaire et logistique multivarié permettront d'ajuster ces comparaisons sur l'âge et le score de Rankin antérieur.

6.2.2. Justification statistique du nombre d'inclusions

Dans une méta analyse de deux études comparant un lever précoce à un protocole « standard » , Craig et collaborateurs (9) ont montré que le pourcentage de patients avec un score de Rankin compris entre 0 et 2 était de 34.7 % à 3 mois dans le groupe lever classique (n=49) versus 57.4 % dans le groupe lever précoce (n=54).

Cette étude vise à démontrer que le score de Rankin est meilleur dans le groupe lever précoce par rapport au groupe lever progressif. L'hypothèse est que le pourcentage de patients avec un score compris entre 0 et 2 est égal à 35 % dans le groupe lever progressif versus 50 % dans le groupe lever précoce. Avec un risque de première espèce fixé à 5 % en situation bilatérale et une puissance à 80 %, 183 patients par groupe sont nécessaires pour mettre en évidence cette différence, soit un total de 366 patients.

Le pourcentage de malaise au lever est estimé entre 5 à 10 % dans les 2 groupes. Cet événement complique l'évaluation du score de Rankin à 3 mois. Un total de 400 patients sera donc inclus.

Si la supériorité du groupe lever précoce n'est pas démontrée (différence statistiquement non significative), un switch vers la non-infériorité sera envisagé. Pour cette analyse, le seuil de non-infériorité serait fixé à -5 % (différence maximale cliniquement acceptable). Avec un effectif de 400 patients, la non-infériorité pourra être démontrée si la différence réelle entre le 2 groupes est comprise entre 9 et 15 %.

Référence : Committee for Proprietary Medical Products (CPMP). Points to consider on switching between superiority and non inferiority. 2000. European Medical Agencies (AMEA). CPMP/EWP/492/99.

L'étude inclura 11 centres. La plupart a un potentiel de recrutement d'au moins 300 infarctus cérébraux par an, ce qui rend parfaitement réaliste l'inclusion de 400 patients.

6.2.3. Degré de signification statistique prévu

Le degré de signification des tests statistiques sera fixé à 5 %. En cas de switch vers la non infériorité, un intervalle de confiance bilatéral à 95 % sera construit et la borne inférieure de l'intervalle sera confrontée au seuil de non infériorité définie à -5 %.

6.2.4. Critères statistiques d'arrêt de la recherche

NA.

6.2.5. Méthode de prise en compte des données manquantes, inutilisées ou non valides

En cas d'effet indésirable nécessitant de recoucher le malade lors du lever du patient ou de décès du patient (peu probable), le score de Rankin sera pénalisé à 3 mois et considéré compris entre (3-5).

Aucune imputation ne sera effectuée sur les critères secondaires.

6.2.6. Gestion des modifications apportées au plan d'analyse de la stratégie initiale

Un plan d'analyse statistique sera rédigé avant le gel de la base de données et détaillera les éventuelles modifications apportées au paragraphe statistique du protocole.

6.2.7. Choix des personnes à inclure dans les analyses

Pour l'analyse de supériorité, la population principale d'analyse sera la population en « **Intention de Traiter** ». Cette population correspond à l'ensemble des patients randomisés dans l'étude. Une analyse de sensibilité sur la population « Per protocol » sera réalisée pour vérifier la robustesse des résultats. Cette population comprend les patients les plus respectueux du protocole : respect des critères d'inclusion et de non-inclusion, absence de déviations majeures au protocole et disponibilité du critère principal. Cette analyse exclura les éventuels patients décédés et les patients ayant eu un EI au lever.

Dans les analyses de non-infériorité, les analyses en ITT et en PP ont la même importance. En cas de switch vers la non infériorité, les 2 analyses seront faites en parallèle et les résultats des analyses seront considérés pertinents si les conclusions sont les mêmes dans les 2 analyses, conformément aux recommandations pour les analyses de non infériorité "Points to consider on switching between superiority and non-inferiority" - Guideline EMEA (les deux analyses ont une égale importance)."

6.2.8. Randomisation

La liste de randomisation sera crée par un statisticien du département de la promotion de la Recherche du CHU de Nantes. La randomisation sera effectuée par blocs selon un ratio 1 : 1.

Une enveloppe contenant le numéro de randomisation et le groupe de traitement sera créée pour chaque patient. Ces enveloppes seront numérotées et conservées dans les services des différents centres.
Pour chaque inclusion, l'investigateur ouvrira une enveloppe suivant l'ordre numérique.

7. VIGILANCE ET GESTION DES EVENEMENTS INDESIRABLES

7.1. DEFINITIONS

7.1.1. Evénements indésirables

Un évènement indésirable (EvI) est défini comme toute manifestation nocive chez un patient ou un participant à un essai clinique, et qui n'est pas nécessairement lié à la méthode étudiée.

Tous les événements indésirables rencontrés au cours de l'étude, qui sont constatés par le médecin ou rapportés par le patient, seront consignés dans le cahier d'observation dans la section prévue à cet effet.

L'intensité des événements indésirables sera cotée selon la cotation suivante :
1 = bénin
2 = modéré
3 = sévère
4 = mettant en jeu le pronostic vital

7.1.2. Effets Indésirables

On considère qu'il y a suspicion d'effet indésirable (EI) pour tout événement indésirable pour lequel un lien de causalité, quelque soit son importance (douteux, plausible, possible, certain) peut être envisagé avec l'essai.

7.1.3. Evènements ou effets indésirables graves

Un EvI ou un EI est considéré comme un EvIG/EIG dès lors qu'il:

* entraîne le décès,
* met en jeu le pronostic vital,
* entraîne une incapacité ou une invalidité temporaire ou définitive,
* nécessite ou prolonge une hospitalisation du patient,
* entraîne une anomalie congénitale ou néonatale,
* est médicalement important (ce qui signifie : nécessite une prise en charge pour éviter l'aggravation vers un des stades ci-dessus).

7.1.4. Effets ou évènements indésirables attendus

Un EvI ou un EI attendu est un événement (effet) déjà mentionné dans la version la plus récente des informations relatives aux méthodes utilisées dans l'essai.

Rappel : Les effets ou évènements indésirables graves attendus feront l'objet d'une déclaration différée par le promoteur auprès des autorités compétentes.

7.1.5. Effets indésirables inattendus

Un effet indésirable inattendu est un effet dont la nature, la sévérité, la fréquence ou l'évolution ne concordent pas avec les informations relatives aux actes pratiqués et méthodes utilisées au cours de l'essai.
Rappel : Les effets indésirables graves inattendus feront l'objet d'une déclaration dans les 7 ou 15 jours suivant leur prise de connaissance par le promoteur auprès des autorités compétentes.

7.2. LISTE DES EI ATTENDUS

Concernant les méthodes à l'étude :

Dans les cas suivants l'imputabilité de l'effet indésirable à la méthode étudiée est évoquée devant l'apparition des événements pendant et dans les minutes suivantes. En dehors de cette relation temporelle, l'effet indésirable pourra être en lien avec l'évolution de la pathologie.

<u>Evénements Indésirables communs au « Lever précoce » et au « Lever tardif » :</u>
- Nausées/Vomissements
- Céphalées
- Aggravation neurologique
- Poussée tensionnelle persistante sur une deuxième mesure avec augmentation de la tension artérielle de plus de 40mmHg et > 18/10
- Malaise (lié à une manifestation vagale (bradycardie, nausées, sueurs) / Hypotensif / Autre)
- Chute pouvant être accompagnée de traumatisme crânien ou de complications orthopédiques (fractures, luxations, entorses)
- Aggravation neurologique potentielle (majoration des déficits initiaux, apparition de nouveaux déficits sensitivomoteurs, troubles visuels, troubles phasiques)

<u>Lever progressif : Complications du décubitus durant l'hospitalisation</u>
- Thrombose veineuse profonde,
- Pneumopathie d'inhalation,
- Infections urinaires,
- Constipation,
- Escarres,
- Troubles de la déglutition

Concernant la pathologie :
- Nausées/Vomissements

- Poussée tensionnelle persistante sur une deuxième mesure avec augmentation de la tension artérielle de plus de 40mmHg et >18/10
- Malaise (lié à une manifestation vagale comme bradycardie, nausées, sueurs / Hypotensif / Autre)
- Chute pouvant être accompagnée de traumatisme crânien ou de complications orthopédiques (fractures, luxations, entorses)
- Aggravation ou apparition de nouveaux symptômes sur le plan neurologique
- Crise comitiale partielle ou généralisée
- Trouble de la vigilance
- Coma
- Décès en lien direct avec la pathologie par aggravation des lésions cérébrales (extension de la zone infarcie, hémorragie cérébrale, œdème, engagement, convulsions,..) ou indirectement liés (décompensation d'une autre pathologie, notamment cardiaque, embolie pulmonaire, sepsis sévère, pneumopathie hypoxémiante…)
- Céphalée
- Trouble de la déglutition
- Pneumopathie
- Thrombose veineuse profonde

Concernant les co-pathologies :
Les co-pathologies présentées par le patient peuvent compliquer son état clinique, notamment pour cette catégorie de patient vasculaire nous pourrons noter des événements en lien avec une cardiopathie ischémique, un diabète,…

Concernant les traitements en cours :
Les traitements pris par le patient dans le contexte de sa pathologie peuvent avoir leurs propres effets indésirables. Dans ce contexte le document de référence pour les EI est le Résumé des Caractéristiques du produit disponible sur le site de l 'Afssaps.

7.3. GESTION DES EVENEMENTS INDESIRABLES

7.3.1. Notification des EIG

Tout EIG (sauf exception spécifiée dans le paragraphe précédent) nécessite le remplissage d'un rapport de survenue d'EIG, qu'il soit attendu ou non attendu. L'investigateur doit vérifier que les informations renseignées sur ce feuillet sont précises et claires (ne pas mettre d'abréviation…).
L'EIG doit être rapporté immédiatement (dans les 24 heures qui suivent sa mise en évidence par l'investigateur) au promoteur par fax (Département promotion de la Direction de la recherche, CHU de Nantes Fax 02 53 48 28 36).
Après réception de la notification d'un EIGI, le promoteur le déclare aux autorités de tutelle. Une fois par an, il établit un rapport annuel de sécurité.

Après réception de la notification d'un EIGI (ou SUSAR : suspected unexpected adverse reaction), le promoteur le déclare aux autorités de tutelle et au CPP.
Une fois par an, il établit un rapport annuel de sécurité."

<u>Précisions</u> :

- L'hospitalisation n'est pas un événement indésirable en soi, c'est un critère de gravité lorsqu'elle est motivée par un élément clinique délétère pour le patient : ainsi les hospitalisations

programmées pour la prise en charge normale habituelle du patient dans le cadre de sa pathologie ou d'une pathologie préexistante, de même celles décidées pour des raisons logistiques (examen sur 2j pour raison technique, problème d'ambulance, etc…) ne sont pas des EvIG.
Seuls les cas d'hospitalisation › ou égal 24h seront à déclarer en tant qu'EIG.

- Le décès est à la fois un EvI et un critère de gravité, mais il est impératif de s'attacher à préciser les causes du décès pour l'interprétation des données bénéfice/risque.

- Le critère de gravité « mise en jeu du pronostic vital » implique dans notre cas la nécessité d'un transfert dans une structure de soins intensifs ou de réanimation.

7.3.2. Comité de surveillance indépendant

Le CIS est un comité consultatif chargé de donner un avis au promoteur et au coordonnateur/ investigateur principal de l'étude sur la sécurité d'un essai clinique. Ses membres compétents dans le domaine des essais cliniques (pathologie et méthodologie) ne sont pas impliqués dans l'étude. Ils sont nommés pour la durée de l'étude et s'engagent sur leur participation comme sur le respect de la confidentialité des données. Le choix des membres du CIS est fait de façon collégiale par le coordonnateur /investigateur principal et le promoteur. Le CIS est destinataire des RAS et peut être sollicité par le vigilant si un SUSAR ou un EIG présente une difficulté particulière d'analyse ou si un doute sur le bénéfice risque apparaît en cours d'étude.

La liste des membres du CIS :
Dr Caroline Arquizan, CHU Montpellier
Dr Marie Hélène Mahagne, CHU Nice
Pr Patrick Mismetti, CHU St Etienne

7.4. MODALITES ET DUREE DU SUIVI DES PERSONNES SUITE A LA SURVENUE D'EVENEMENTS INDESIRABLES

En cas d'événement indésirable grave ayant entraîné la sortie de l'étude ou d'événement persistant à la fin de l'étude, le patient sera revu jusqu'à la résolution de l'évènement

8. ASPECTS ADMINISTRATIFS ET REGLEMENTAIRES

8.1. DROIT D'ACCES AUX DONNEES ET DOCUMENTS SOURCE

Les données médicales de chaque patient ne seront transmises qu'au promoteur ou toute personne dûment habilitée par celui-ci, et, le cas échéant aux autorités sanitaires habilitées, dans les conditions garantissant leur confidentialité.
Le promoteur et les autorités de tutelle pourront demander un accès direct au dossier médical pour vérification des procédures et/ou des données de l'essai clinique, sans violer la confidentialité et dans les limites autorisées par les lois et réglementations.
Les données recueillies lors de l'essai pourront faire l'objet d'un traitement informatique, en conformité avec les exigences de la CNIL (conformité à la méthodologie de référence MR001).

8.2. MONITORING DE L'ESSAI

Le monitoring sera assuré par le Département promotion de la Direction de la recherche. Un Attaché de Recherche Clinique (ARC) se rendra régulièrement sur chaque site afin de procéder au contrôle qualité des données rapportées dans les cahiers d'observations.

Le protocole a été classé selon le niveau risque estimé pour le patient se prêtant à la recherche. Il sera suivi de la manière suivante :
Risque B : risque prévisible proche de celui des soins usuels

Les visites de monitoring sur site seront organisées après rendez-vous avec l'investigateur. Les ARC devront pouvoir consulter :
- les cahiers de recueil de données des patients inclus,
- les dossiers médicaux et infirmiers des patients,
- le classeur investigateur.

8.3. INSPECTION / AUDIT

Dans le cadre de la présente étude, une inspection ou un audit pourra avoir lieu.

8.4. CONSIDERATIONS ETHIQUES

8.4.1. Consentement éclairé écrit

L'investigateur s'engage à informer le patient de façon claire et juste du protocole et à lui demander un consentement éclairé et écrit (note d'information et formulaire de recueil de consentement en annexe). Il remettra au patient un exemplaire de la note d'information et un formulaire de recueil de consentement. Le patient ne pourra être inclus dans l'étude qu'après

avoir pris connaissance de la note d'information et avoir signé et daté le formulaire de recueil de consentement. L'investigateur doit également signer et dater le formulaire de recueil de consentement. Ces deux documents seront délivrés sur papier en 2 exemplaires minimum afin que le patient et l'investigateur puissent chacun en garder un exemplaire. L'original de l'investigateur sera classé dans le classeur investigateur.

Le protocole peut être mis en œuvre en situation d'urgence. Ainsi, en cas de trouble phasique du patient, l'information et le recueil du consentement pourra se faire auprès d'une personne de confiance, d'un membre de la famille, ou auprès d'une personne entretenant avec l'intéressé des liens étroits et stables (conformément à l'article L. 1122-2. II du code de la santé publique). Une lettre d'information et un consentement sont prévus à cet effet (annexe 7 et 9)
Le protocole peut être mis en œuvre en situation d'urgence, ainsi en cas de trouble phasique du patient, l'information et le recueil du consentement pourra se faire auprès d'un proche. Une lettre d'information et un consentement sont prévus à cet effet (annexe 7 et 9)

Une procédure d'urgence a également été prévue. Ce recueil de consentement réalisé auprès d'une personne de confiance, d'un membre de la famille, ou auprès d'une personne entretenant avec l'intéressé des liens étroits et stables (conformément à l'article L. 1122-2. II du code de la santé publique) pourra ainsi être réalisé dans un premier temps par l'investigateur ou par un médecin qui le représente de manière téléphonique puis dans un deuxième temps par écrit de façon différée et le plus rapidement possible.
Ce recueil de consentement d'un proche pourra se faire de façon téléphonique et signé de façon différée dans les 24 heures. En effet, il s'agit ici d'une étude de phase précoce et les patients doivent être randomisés rapidement. Le fonctionnement des services fait que les visites médicales ne correspondent pas en terme d'horaire avec les visites des familles, qui se déroulent généralement l'après midi. Il est probable qu'à ce moment les équipes paramédicales ne soient pas forcement disponibles pour effectuer les procédures de lever, qui se font en général le matin ou en tout début d'après midi. Dans le cas d'un patient aphasique ne pouvant donner son consentement pendant la visite, l'inclusion dans l'étude ne sera donc probablement pas réalisable en pratique. Ceci pourrait créer un biais dans le travail (non inclusion d'un sous groupe de patient), puisque environ 30% des patients victimes d'infarctus cérébral sont aphasiques. S'agissant d'une Recherche Biomédicale sur une modalité de soin et non une molécule, et considérant que l'étude peut être facilement expliquée et comprise par appel téléphonique, nous considérons que le recueil du consentement d'un proche par téléphone peut être proposé dans ce cadre (patient aphasique sans proche physiquement présent au moment de l'inclusion potentielle). La signature du consentement écrit par le proche devra se faire dans les 24 heures qui suivent l'accord téléphonique.

Le recours à cette procédure d'urgence devra faire l'objet d'une traçabilité. Ainsi, le contact téléphonique durant lequel le proche a donné son accord oral devra impérativement être notifié dans le dossier médical du patient.

Le patient sera informé dés que possible et son consentement sera recherché pour la poursuite de cette recherche.

Le promoteur s'engage à soumettre le projet d'étude à l'autorisation préalable d'un Comité de Protection des Personnes (CPP). Les informations communiquées portent d'une part sur les modalités et la nature de la recherche et d'autre part, sur les garanties prévues pour les patients participant à cet essai.

8.5. AMENDEMENTS AU PROTOCOLE

Les demandes de modifications substantielles seront adressées par le promoteur pour autorisation ou information auprès de l'Afssaps et /ou au comité de protection des personnes concerné conformément à la loi 2004-806 du 9 août 2004 et ses arrêtés d'application.

Le protocole modifié devra faire l'objet d'une version actualisée datée.
Les formulaires d'information et de recueil consentement du patient devront faire l'objet de modification si nécessaire.

8.6. DECLARATION AUX AUTORITES COMPETENTES

Le présent protocole fera l'objet d'une demande d'autorisation auprès de l'Afssaps.

8.7. FINANCEMENT ET ASSURANCE

Le promoteur assure le financement de l'étude et souscrit une police d'assurance garantissant les conséquences pécuniaires de sa responsabilité civile, conformément à la réglementation.

8.8. REGLES RELATIVES A LA PUBLICATION

Une copie de la publication sera remise au CHU de Nantes, responsable de la recherche de l'étude qui sera nécessairement cité. Les auteurs seront déterminés au prorata du nombre de patients inclus. La liste des auteurs sera établie par le Dr Fanny Hérisson.

LISTE DES ANNEXES

Annexe 1 : Score de Rankin

Annexe 2 : Score de NHSS

Annexe 3: Index de Barthel

Annexe 4 : Listing des investigateurs (détachée du protocole)

Annexe 5 : Références bibliographiques (détachée du protocole)

Annexe 6 : Lettre d'information patient (détachée du protocole)

Annexe 7 : Lettre d'information en situation d'urgence (proche) (détachée du protocole)

Annexe 8 : Formulaire de recueil de consentement patient (détachée du protocole)

Annexe 9 : Formulaire de recueil de consentement en situation d'urgence (détachée du protocole)

Annexe 10 : Formulaire d'Evénement Indésirable Grave (détachée du protocole)

ANNEXE 1 : SCORE DE RANKIN

Valeur	Symptômes
0	Aucun symptôme
1	Pas d'incapacité en dehors des symptômes : activités et autonomie conservées
2	Handicap faible : incapacité dans les activités habituelles mais autonomie
3	Handicap modéré : besoin d'aide dans les activités de la vie quotidienne mais marche possible sans assistance
4	Handicap modérément sévère : marche et gestes quotidiens impossibles sans aide
5	Handicap majeur : alitement permanent, incontinence et soins de nursing permanent
6	Décès

ANNEXE 2 : SCORE NIHSS

Horaire de l'évaluation :

1a Niveau de conscience →
0 = éveillé, bonne réactivité
1 = réduction vigilance mais réveillable par stimulation mineure (question,...)
2 = réduction vigilance ; réveillable par stimulations répétées ou douloureuses
3 = coma avec réponse motrice stéréotypée ou végétative à la stimulation douloureuse

1b - Réponses aux questions (quel mois ? quel âge ?) →
0 = répond correctement aux 2 questions
1 = répond correctement à une seule question
2 = pas de réponses correctes

1c - Réponses aux consignes (fermer les yeux ; serrer le poing) →
0 = exécute correctement les 2 consignes
1 = exécute une seule consigne
2 = n'exécute aucune consigne

2 – Oculo-motricité (mouvement d'horizontalité du regard, volontaire ou réflexe)
0 = normale →
1 = paralysie partielle (regard anormal sur 1 ou les 2 yeux, sans déviation forcée du regard ni paralysie complète)
2 = déviation forcée ou paralysie complète du regard persistant dans les réflexes oculo-céphaliques

3 – Champ visuel →
0 = normal
1 = hémianopsie partielle
2 = hémianopsie complète
3 = hémianopsie bilatérale (inclus cécité corticale)

4 - Motricité faciale →
0 = normale
1 = PF mineure (effacement sillon naso-génien, asymétrie du sourire)
2 = PF partielle (totale ou presque sur le facial inférieur)
3 = PF complète (inférieur et supérieur)

5a – Motricité membre supérieur Droit (bras tendus à 45° durant 10″) →
0 = pas de chute
1 = chute avant 10″, sans retomber sur le plan du lit
2 = effort contre la pesanteur possible sans atteindre ou tenir la position
3 = pas de motricité contre la pesanteur
4 = déficit complet

5b – Motricité membre supérieur Gauche (bras tendus à 45° durant 10″) →
0 = pas de chute
1 = chute avant 10″, sans retomber sur le plan du lit
2 = effort contre la pesanteur possible sans atteindre ou tenir la position
3 = pas de motricité contre la pesanteur
4 = déficit complet

6a – Motricité membre inférieur Droit (jambes tendus à 30° durant 5″) →
0 = pas de chute
1 = chute avant 5″, sans retomber sur le plan du lit
2 = effort contre la pesanteur, mais la jambe chute sur le lit
3 = pas d'effort contre la pesanteur
4 = déficit complet

6b – Motricité membre inférieur Gauche (jambes tendus à 30° durant 5″) →
0 = pas de chute
1 = chute avant 5″, sans retomber sur le plan du lit
2 = effort contre la pesanteur, mais la jambe chute sur le lit
3 = pas d'effort contre la pesanteur
4 = déficit complet

7 – Coordination des gestes (en l'absence de déficit significatif) →
0 = normale
1 = ataxie sur un membre
2 = ataxie sur les 2 membres

8 – Sensibilité →
0 = normale
1 = hypo-esthésie
2 = anesthésie

9 – Langage →
0 = normal
1 = aphasie modérée (perte de fluence verbale ; trouble de la compréhension sans limitation des idées exprimées ni de la forme d'expression)
2 = aphasie sévère (expression fragmentaire, dénomination des objets impossibles)
3 = aphasie globale (mutisme, perte de la compréhension)

10 – Articulation →
0 = normale
1 = dysarthrie modérée (discours reste compris)
2 = dysarthrie sévère (discours incompréhensible sans proportion avec une éventuelle aphasie ; anarthrie, mutisme)

11 – Extinction et négligence →
0 = pas d'anomalie
1 = négligence ou extinction visuelle, auditive, tactile aux stimulations bilatérales simultanées
2 = héminégligence sévère ou extinction dans plusieurs modalités sensorielles ; ne reconnaît pas sa main ou s'oriente vers un seul hémi-espace

SCORE TOTAL *(addition des 15 sous-scores)*

ANNEXE 3 : INDEX DE BARTHEL

**Items**	**Ne fait pas**	**Avec aide**	**Indépendant**
Alimentation (avec aide si nécessaire pour couper les aliments)	0	5	10
Transfert du fauteuil au lit et retour (peut s'asseoir dans son lit)	0	5 (aide majeure)<br>10 (aide mineure)	15
Toilette personnelle (se laver le visage, se coiffer, se raser, se laver les dents)		0	5
Transfert aux et des toilettes (y compris déshabillage, s'essuyer, tirer la chasse)	0	5	10
Se baigner seul		0	5
Marche en terrain plat (ou si marche impossible, utilisation du fauteuil roulant)	0	5 (fauteuil roulant)<br>10 (marche avec assistance)	15
Monter et descendre les escaliers	0	5	10
Habillage (y compris nouer les lacets, attacher les fermetures)	0	5	10
Contrôle intestinal	0	5 (incontinence occasionnelle)	10
Contrôle vésical	0 (incontinent)	5 (incontinence occasionnelle)	10
**Total =**			
