# Supplementary material for: Early Sitting in Ischemic Stroke Patients (SEVEL): A Randomized Controlled Trial
Source: PLoS One. 2016 Mar 29;11(3):e0149466. doi: 10.1371/journal.pone.0149466 (PMC4811411; doi:10.1371/journal.pone.0149466)
Supplement: S3 Protocol — (PDF) [file pone.0149466.s004.pdf]

ANNEXE 5 : REFERENCES BIBLIOGRAPHIQUES

1. Haute Autorité de Santé. Accidents vasculaires cérébraux : prise en charge précoce. ; 2009.
2. Wojner-Alexander AW, Garami Z, Chernyshev OY, Alexandrov AV. Heads down: flat positioning improves blood flow velocity in acute ischemic stroke. Neurology 2005 64(8):1354-7.
3. Adams HP Jr, del Zoppo G, Alberts MJ, Bhatt DL, Brass L, Furlan A, Grubb RL, Higashida RT, Jauch EC, Kidwell C, Lyden PD, Morgenstern LB, Qureshi AI, Rosenwasser RH, Scott PA, Wijdicks EF; American Heart Association; American Stroke Association Stroke Council; Clinical Cardiology Council; Cardiovascular Radiology and Intervention Council; Atherosclerotic Peripheral Vascular Disease and Quality of Care Outcomes in Research Interdisciplinary Working Groups. Guidelines for the early management of adults with ischemic stroke: a guideline from the American Heart Association/American Stroke Association Stroke Council, Clinical Cardiology Council, Cardiovascular Radiology and Intervention Council, and the Atherosclerotic Peripheral Vascular Disease and Quality of Care Outcomes in Research Interdisciplinary Working Groups: the American Academy of Neurology affirms the value of this guideline as an educational tool for neurologists. Stroke 2007 38(5):1655-711.
4. The European Stroke Organization (ESO) Executive Committee" and the "ESO" Writing Committee Guidelines for Management of Ischaemic Stroke and Transient Ischaemic Attack 2008.
5. van der Worp HB., Kappelle LJ. Complications of acute ischaemic stroke. Cerebrovasc Dis 1998;8:124-32.
6. Johnston KC, Li JY, Lyden PD, Hanson SK, Feasby TE, Adams RJ,Faught RE Jr, Haley EC Jr; RANTTAS Investigators. Medical and neurological complications of ischemic stroke: experience from the RANTTAS trial. Stroke 1998;29:447– 53.
7. Langhorne P, Stott DJ, Robertson L, MacDonald J, Jones L, McAlpine C, Dick F, Taylor GS, Murray G. . Medical complications after stroke: a multicenter study. Stroke 2000;31:1223–9.
8. Ingeman A, Andersen G, Hundborg HH, Svendsen ML, Johnsen SP. Processes of care and medical complications in patients with stroke. Stroke 2011 42(1):167-72.
9. Craig LE, Bernhardt J, Langhorne P, Wu O Early mobilization after stroke: an example of an individual patient data meta-analysis of a complex intervention. . Stroke 2010 41(11):2632-6.
10. Carrera E, Kim DJ, Castellani G, Zweifel C, Smielewski P, Pickard JD, Kirkpatrick PJ, Czosnyka M Cerebral arterial compliance in patients with internal carotid artery disease. Eur J Neurol 2010
11. Klijn Cj, Kappelle Lj. Haemodynamic Stroke: Clinical Features, Prognosis, And Management. . Lancet Neurol 9(10):1008-17.
12. Caplan LR, Wong KS, Gao S, Hennerici MG. . Is hypoperfusion an important cause of strokes? If so, how? Cerebrovasc Dis 2006;21(3):145-53.
13. Zhao H. Ischemic post conditioning as a novel avenue to protect brain injury after stroke. J Cereb Blood Flow Metab 2009;29(5):873-85.
14. Bernhardt J, Thuy MN, Collier JM, Legg LA. Very Early Versus Delayed Mobilization After Stroke. Stroke 2009
15. Langhorne P, Stott D, Knight A, Bernhardt J, Barer D, Watkins C. Very early rehabilitation or intensive telemetry after stroke: a pilot randomised trial. . Cerebrovasc Dis 2010;29(4):352-60.
16. Cumming TB, Thrift AG, Collier JM, Churilov L, Dewey HM, Donnan GA, Bernhardt J. Very Early Mobilization After Stroke Fast-Tracks Return to Walking: Further Results From the Phase II AVERT Randomized Controlled Trial. Stroke 2011 42(1):153-8.
17. Kasner SE. Clinical interpretation and use of stroke scales. Lancet Neurol 2006 5(7):603-12.
18. Brott T, Adams HP Jr, Olinger CP, Marler JR, Barsan WG, Biller J, Spilker J, Holleran R, Eberle R, Hertzberg V, et al. Measurements of acute cerebral infarction: a clinical examination scale. Stroke 1989 20(7):864-70.
19. Shinar D, Gross CR, Bronstein KS, Licata-Gehr EE, Eden DT, Cabrera AR, Fishman IG, Roth AA, Barwick JA, Kunitz SC. Reliability of the activities of daily living scale and its use in telephone interview. Arch Phys Med Rehabil 1987 68(10):723-8.
