# Supplementary material for: Early Sitting in Ischemic Stroke Patients (SEVEL): A Randomized Controlled Trial
Source: PLoS One. 2016 Mar 29;11(3):e0149466. doi: 10.1371/journal.pone.0149466 (PMC4811411; doi:10.1371/journal.pone.0149466)
Supplement: S4 Protocol — (PDF) [file pone.0149466.s005.pdf]

LETTRE D'INFORMATION PATIENT

Note d'information pour la participation à la recherche

TITRE DE L'ETUDE CLINIQUE :

« SEVEL : Verticalisation des patients à la phase aigue à la suite d'un infarctus cérébral »

Promoteur : CHU Nantes n°ID RCB: 2011-A00430-41

Médecin Investigateur : …………………………………………………..

Madame, Mademoiselle, Monsieur,

Nous sollicitons votre participation afin d'effectuer un travail concernant le lever des patients au début de la prise en charge des infarctus cérébraux (une artère se bouche dans le cerveau), pathologie pour laquelle vous êtes actuellement hospitalisé(e). L'étude s'intitule :

« SEVEL: Verticalisation des patients à la phase aigue à la suite d'un infarctus cérébral »

Vous concrétiserez votre accord en signant un formulaire de consentement éclairé, dont vous garderez un exemplaire. Votre signature ainsi que celle du médecin sont indispensables. Vous garderez le droit d'interrompre à tout moment votre participation sans avoir à vous justifier.

Le CHU de Nantes est le promoteur de cette recherche, c'est à dire qu'il en est responsable et qu'il l'organise.

Quel est l'objectif de cette étude ?

Nous allons observer deux groupes de patients : le premier pour lequel le lever (mise au fauteuil) aura lieu le lendemain de l'infarctus (groupe lever précoce) et le second pour lequel le lever sera progressif sur 3 jours (groupe lever progressif)

Il n'existe à l'heure actuelle aucune recommandation concernant le lever des patients au début de la prise en charge des infarctus cérébraux, et les pratiques sont très variables d'un service à l'autre.

De nombreux médecins laissent le patient couché de façon prolongée de peur que le lever au fauteuil aggrave les symptômes. Cette aggravation potentielle est possible, même si elle est très rare et souvent transitoire en pratique (dans ce cas vous seriez recouché aussitôt). De plus, il n'existe aucune preuve scientifique que le fait de se lever assez tôt après l'infarctus cérébral puisse être néfaste.

A contrario, le fait de rester couché de façon prolongée peut ralentir la récupération, la reprise de l'autonomie et favoriser la survenue de complications spécifiques (constipation, retention d'urine, phébite, infections,…) dans un nombre non négligeable de cas.

Cette étude est donc nécessaire afin d'établir la meilleure stratégie de lever (précoce ou différé) et se déroule dans plusieurs centres en France.

Si vous décidez de participer à cette recherche, il s'agira d'un acte volontaire. Vous pourrez à tout moment décider d'arrêter votre participation, sans pénalité ni préjudice. Dans ce cas, vous devez informer le médecin de votre décision. Dans tous les cas, la qualité de votre prise en charge ne sera pas diminuée.

Qu'arrivera t-il pendant la recherche ?
Si vous acceptez de participer à cette étude, un tirage au sort sera effectué pour déterminer dans quel groupe vous serez affecté : lever précoce ou lever progressif.
Tout le reste de la prise en charge restera identique.

Lors de cette étude, 2 visites sont prévues :
- une visite 7 jours après votre infarctus cérébral (ou jour de votre sortie d'hospitalisation)
- une visite à 3 mois (prise en charge habituelle)

Lors de ces visites, un examen clinique sera réalisé afin d'évaluer votre état de récupération et votre autonomie.

Votre participation à cette recherche durera ainsi 3 mois.
Les patients inclus dans l'étude ne pourront pas participer à une autre recherche clinique pendant la durée de l'étude.

Quels sont les éventuels bénéfices associés à cette participation ?
Pour le patient participant à l'étude, une surveillance et un suivi particuliers sont mis en place, avec notamment une visite systématique à 3 mois.

Le bénéfice de ce travail pour les patients victimes d'AVC (150 000 cas par an en France) est donc très important pour que la prise en charge soit la meilleure, et qu'elle soit homogène dans tous les centres.

Quels sont les éventuels désavantages et inconvénients associés à cette participation ?
Cette étude ne présente pas de contrainte particulière pour le patient. Le fait d'être installé au fauteuil est un acte pratiqué usuellement, de même que la visite de suivi à 3 mois.

Que se passera-t-il à la fin de la recherche, si la recherche s'arrête ou si vous décidez d'interrompre votre participation ?
La recherche peut être interrompue à tout moment:

- par les autorités de santé,

- du fait du promoteur, le CHU de Nantes : si un élément nouveau survient, le médecin-investigateur en sera informé et il vous transmettra alors les éléments susceptibles de modifier votre participation.

- du fait du médecin-investigateur, pour des raisons médicales vous concernant : il peut décider à tout moment d'arrêter le suivi de la recherche (par exemple à cause d'un effet secondaire ou d'une évolution de votre état de santé) et vous en informera.

- par vous-même : si vous décidez de participer à cette recherche, il s'agira d'un acte volontaire. Vous pourrez à tout moment décider d'arrêter votre participation, sans pénalité ni préjudice. Dans ce cas, vous devez informer le médecin-investigateur de votre décision.

Quelle que soit la raison de votre interruption, le médecin-investigateur vous informera alors des mesures à suivre.

Dans tous les cas, la qualité de votre prise en charge ne sera pas diminuée.

Aurais-je des frais supplémentaires ?
Votre participation à cette recherche n'engendrera pour vous aucun frais supplémentaire par rapport à ceux que vous auriez dans la prise en charge habituelle de cette maladie.

Quels sont mes obligations et mes droits pendant la recherche ?

- **Protection sociale**

Pour pouvoir participer à cette recherche vous devez être affilié(e) ou bénéficier d'un régime de sécurité sociale (CMU acceptée).

- **Secret professionnel**

Le personnel impliqué dans la recherche est soumis au secret professionnel, tout comme votre médecin traitant.

- **Accès aux données vous concernant - Traitement des données**

Dans le cadre de cette recherche, un traitement informatique de vos données personnelles va être mis en œuvre : cela permettra d'analyser les résultats de la recherche et de remplir l'objectif de la recherche.
Pour cela, les données médicales vous concernant (et les données relatives à vos habitudes de vie), seront transmises au Promoteur de la recherche (CHU de Nantes). Ces données seront identifiées par un numéro de code et vos initiales.
Ces données pourront également, dans des conditions assurant leur confidentialité, être transmises aux autorités de santé françaises ou étrangères. Conformément aux dispositions de la loi relative à l'informatique aux fichiers et aux libertés (loi modifiée du 6 janvier 1978), vous disposez d'un droit d'accès et de rectification. Vous disposez également d'un droit d'opposition à la transmission des données couvertes par le secret professionnel susceptibles d'être utilisées dans le cadre de cette recherche et d'être traitées.
Ces droits s'exercent auprès du médecin-investigateur qui vous suit dans le cadre de la recherche et qui connaît votre identité.
Vous pouvez également accéder directement ou par l'intermédiaire d'un médecin de votre choix à l'ensemble de vos données médicales en application des dispositions de l'article L 1111-7 du Code de la Santé Publique.

- **Accès aux résultats globaux de la recherche**

A la fin de la recherche biomédicale, et à votre demande, vous pourrez être informé(e) par le médecin-investigateur des résultats globaux de cette recherche (dès qu'ils seront disponibles)

Le Cadre réglementaire

Cette recherche biomédicale est conforme :

- Aux articles L. 1121-1 à L. 1126-7 du code de la santé publique relatifs aux recherches biomédicales,

- A la loi « Informatique et Libertés » du 6 janvier 1978 modifiée.

(vous pouvez retrouver tous ces textes sur le site http://www.legifrance.gouv.fr)

Conformément aux dispositions réglementaires :

- Le CHU de Nantes organise cette recherche en tant que « promoteur ». Il a souscrit un contrat d'assurance garantissant sa responsabilité civile et celle de tout intervenant auprès de la compagnie SHAM (contrat n°135 964).
- Cette recherche a reçu l'avis favorable du Comité de Protection des Personnes Ouest IV (Nantes) le //2011. La recherche a aussi reçu l'autorisation de l'AFSSAPS (Agence Française de Sécurité Sanitaire des Produits de Santé), le //2011.

Informations complémentaires

Lisez bien cette lettre d'information. Vous pouvez à tout moment demander des informations complémentaires sur cette étude, sur vos droits en tant que patient et notifier un effet indésirable à votre médecin (investigateur de l'étude) :

Dr---
Adresse--
Tel--

Nous vous prions d'agréer, Madame, Mademoiselle, Monsieur, l'expression de nos sentiments les plus respectueux.
