# Supplementary material for: Early Sitting in Ischemic Stroke Patients (SEVEL): A Randomized Controlled Trial
Source: PLoS One. 2016 Mar 29;11(3):e0149466. doi: 10.1371/journal.pone.0149466 (PMC4811411; doi:10.1371/journal.pone.0149466)
Supplement: S5 Protocol — (PDF) [file pone.0149466.s006.pdf]

LETTRE D'INFORMATION EN SITUATION D'URGENCE

Note d'information pour la participation à la recherche

TITRE DE L'ETUDE CLINIQUE :

« SEVEL : Verticalisation des patients à la phase aigue à la suite d'un infarctus cérébral »

Promoteur : CHU Nantes n°ID RCB: 2011-A00430-41

Médecin Investigateur : ………………………………………………..

Madame, Mademoiselle, Monsieur,

En raison de son état, devant l'urgence de la situation et conformément à la loi, c'est à vous que nous demandons l'autorisation de participation de votre proche, Mme, Melle, M. (rayer les mentions inutiles)(nom, prénom)……………………………………………….. …à cette recherche biomédicale.

Nous sollicitons la participation de votre proche afin d'effectuer un travail concernant le lever des patients au début de la prise en charge des infarctus cérébraux, pathologie pour laquelle votre proche est actuellement hospitalisé(e). L'étude s'intitule :

« **SEVEL : Verticalisation des patients à la phase aigue à la suite d'un infarctus cérébral** »

Le CHU de Nantes est le promoteur de cette recherche, c'est à dire qu'il en est responsable et qu'il l'organise.

Comment vous décider?

- Le médecin-investigateur de votre proche vous a donné des explications. Elles sont résumées dans ce document intitulé « lettre d'information ». Nous vous invitons à la lire attentivement avant de vous décider.

- Si vous acceptez la participation de votre proche à cette recherche, on vous demandera de signer une attestation de consentement. Cette attestation sera aussi signée par le médecin. Cette signature confirmera que vous êtes d'accord pour que votre proche participe à la recherche. Votre signature est indispensable, ainsi que celle du médecin. Même après avoir signé pour donner votre accord, vous garderez le droit d'interrompre à tout moment la participation votre proche à la recherche sans avoir à vous justifier, ceci tant que votre proche ne sera pas en état de s'exprimer.

- Dès que son état le permettra, votre proche sera informé par le médecin-investigateur sur cette recherche et son consentement lui sera demandé pour la poursuite éventuelle de la recherche. Votre proche pourra alors arrêter sa participation à la recherche s'il le souhaite.

Quel est l'objectif de cette étude ?

Nous allons observer deux groupes de patients : le premier pour lequel le lever (mise au fauteuil) aura lieu le lendemain de l'infarctus (groupe lever précoce) et le second pour lequel le lever sera progressif sur 3 jours (groupe lever progressif)

Il n'existe à l'heure actuelle aucune recommandation concernant le lever des patients au début de la prise en charge des infarctus cérébraux, et les pratiques sont très variables d'un service à l'autre.

De nombreux médecins laissent le patient couché de façon prolongée de peur que le lever au fauteuil aggrave les symptômes. Cette aggravation potentielle est possible, même si elle est très rare et

souvent transitoire en pratique (dans ce cas vous seriez recouché aussitôt). De plus, il n'existe aucune preuve scientifique que le fait de se lever assez tôt après l'infarctus cérébral puisse être néfaste.
A contrario, le fait de rester couché de façon prolongée peut ralentir la récupération, la reprise de l'autonomie et favoriser la survenue de complications spécifiques (constipation, retention d'urine, phébite, infections,…) dans un nombre non négligeable de cas.

Cette étude est donc nécessaire afin d'établir la meilleure stratégie de lever (précoce ou différé) et se déroule dans plusieurs centres en France.

Dés que possible, votre proche sera informé de cette recherche. Il pourra à tout moment décider d'arrêter sa participation, sans pénalité ni préjudice. Dans ce cas, il devra informer le médecin de sa décision. Dans tous les cas, la qualité de sa prise en charge ne sera pas diminuée.
Nous allons observer deux groupes de patients : le premier pour lequel le lever (mise au fauteuil) aura lieu le lendemain de l'infarctus (groupe lever précoce) et le second pour lequel le lever sera progressif sur 3 jours (groupe lever progressif)

Qu'arrivera t-il pendant la recherche ?
Si vous donnez votre accord pour la participation de votre proche à cette étude, un tirage au sort sera effectué pour déterminer dans quel groupe il/elle sera affecté(e) : lever précoce ou lever progressif.
Tout le reste de la prise en charge restera identique.

Lors de cette étude, 2 visites sont prévues :
- une visite 7 jours après votre infarctus cérébral (ou jour de sortie de d'hospitalisation)
- une visite à 3 mois (suivi habituel)

Lors de ces visites, un examen clinique sera réalisé afin d'évaluer l'état de récupération post-infarctus de votre proche.

La participation à cette recherche durera ainsi 3 mois.
Les patients inclus dans l'étude ne pourront pas participer à une autre recherche clinique pendant la durée de l'étude.

Quels sont les éventuels bénéfices associés à cette participation ?
Pour le patient participant à l'étude, une surveillance et un suivi particuliers sont mis en place, avec notamment une visite systématique à 3 mois.

Le bénéfice de ce travail pour les patients victimes d'AVC (150 000 cas par an en France) est donc très important pour que la prise en charge soit la meilleure, et qu'elle soit homogène dans tous les centres.

Quels sont les éventuels désavantages et inconvénients associés à cette participation ?
Cette étude ne présente pas de contrainte particulière pour le patient. Le fait d'être installé au fauteuil est un acte pratiqué usuellement, de même que la visite de suivi à 3 mois.

Que se passera-t-il à la fin de la recherche, si la recherche s'arrête ou si vous décidez d'interrompre votre participation ?
La recherche peut être interrompue à tout moment:

- par les autorités de santé,

- du fait du promoteur, le CHU de Nantes : si un élément nouveau survient, le médecin-investigateur en sera informé et il transmettra alors, soit à vous, soit à votre proche si son état le lui permet les éléments susceptibles de modifier l'accord de participation à la recherche.

- du fait du médecin-investigateur, pour des raisons médicales concernant votre proche : il peut décider à tout moment d'arrêter le suivi de la recherche (par exemple à cause d'un effet secondaire ou d'une évolution de votre état de santé) et vous en informera ou informera directement votre proche si son état alors le permet.

- par vous-même : si vous acceptez que votre proche participe à cette recherche, il s'agira d'un acte volontaire. Vous pourrez à tout moment, tant que votre proche sera incapable d'exprimer son consentement ou son opposition, décider d'arrêter sa participation sans pénalité ni préjudice. Dans ce cas, vous devez informer le médecin-investigateur de votre décision.

Quelle que soit la raison de l'interruption de la recherche pour votre proche, le médecin-investigateur vous expliquera ou expliquera à votre proche si son état le permet les mesures à suivre.

Dans tous les cas, la qualité de la prise en charge de votre proche ne sera pas diminuée.

Y aura-t-il des frais supplémentaires pour votre proche ?
La participation à cette recherche de votre proche n'engendrera pour lui/elle aucun frais supplémentaire par rapport à ceux qu'il/elle aurait eu dans la prise en charge habituelle de cette maladie.

Quels sont mes obligations et mes droits pendant la recherche ?

❖ **Protection sociale**

Pour pouvoir participer à cette recherche votre proche doit être affilié(e) ou bénéficier d'un régime de sécurité sociale (CMU acceptée).

❖ **Secret professionnel**

Le personnel impliqué dans la recherche est soumis au secret professionnel, tout comme le médecin traitant de votre proche.

❖ **Accès aux données vous concernant - Traitement des données**

Dans le cadre de cette recherche, un traitement informatique des données personnelles de votre proche va être mis en œuvre : cela permettra d'analyser les résultats de la recherche et de remplir l'objectif de la recherche.
Pour cela, les données médicales concernant votre proche (et les données relatives à ses habitudes de vie), seront transmises au Promoteur de la recherche (CHU de Nantes). Ces données seront identifiées par un numéro de code et les initiales de votre proche.
Ces données pourront également, dans des conditions assurant leur confidentialité, être transmises aux autorités sanitaires habilitées. Conformément aux dispositions de la loi relative à l'informatique aux fichiers et aux libertés (loi modifiée du 6 janvier 1978), un droit d'accès et de rectification peut être exercé. Vous et votre proche disposez également d'un droit d'opposition à la transmission des données couvertes par le secret professionnel susceptibles d'être utilisées dans le cadre de cette recherche et d'être traitées.
Ces droits s'exercent auprès du médecin-investigateur qui suit votre proche dans le cadre de la recherche et qui connaît son identité.
Il est également possible d'accéder directement ou par l'intermédiaire d'un médecin de votre choix à l'ensemble des données médicales de votre proche en application des dispositions de l'article L 1111-7 du Code de la Santé Publique.

❖ **Accès aux résultats globaux de la recherche**

A la fin de la recherche biomédicale, et vous et votre proche pourrez être informé(e) par le médecin-investigateur des résultats globaux de cette recherche (dès qu'ils seront disponibles).

Le Cadre réglementaire

Cette recherche est conforme :
- Aux articles L. 1121-1 à L. 1126-7 du code de la santé publique relatifs aux recherches biomédicales,
- A la loi « Informatique et Libertés » du 6 janvier 1978 modifiée.
(Vous pouvez retrouver tous ces textes sur le site http://www.legifrance.gouv.fr)

Conformément aux dispositions réglementaires :

- Le CHU de Nantes organise cette recherche en tant que « promoteur ». Il a souscrit un contrat d'assurance garantissant sa responsabilité civile et celle de tout intervenant auprès de la compagnie SHAM (contrat n°135 964).
- Cette recherche a reçu l'avis favorable du Comité de Protection des Personnes Ouest IV (Nantes) le //2011. La recherche a aussi reçu l'autorisation de l'AFSSAPS (Agence Française de Sécurité Sanitaire des Produits de Santé), le //2011.
Nous vous prions d'agréer, Madame, Mademoiselle, Monsieur, l'expression de nos sentiments les plus respectueux.

Informations complémentaires

Lisez bien cette lettre d'information. Vous pouvez à tout moment demander des informations complémentaires sur cette étude, sur les droits de votre proche en tant que patient et notifier un effet indésirable à son médecin (investigateur de l'étude) :

Dr--
Adresse---
Tel--

Nous vous prions d'agréer, Madame, Mademoiselle, Monsieur, l'expression de nos sentiments les plus respectueux.
