# Supplementary material for: Early Sitting in Ischemic Stroke Patients (SEVEL): A Randomized Controlled Trial
Source: PLoS One. 2016 Mar 29;11(3):e0149466. doi: 10.1371/journal.pone.0149466 (PMC4811411; doi:10.1371/journal.pone.0149466)
Supplement: S6 Protocol — (PDF) [file pone.0149466.s007.pdf]

ANNEXE 8: FORMULAIRE DE RECUEIL DE CONSENTEMENT PATIENT

« SEVEL : Verticalisation des patients à la phase aigue à la suite d'un infarctus cérébral »

Promoteur : CHU Nantes n°ID RCB: n°2011-A0043 0-41

Je soussigné(e)

Me, Mlle, M. *(rayer les mentions inutiles)* (*prénom*, *nom)*……………………………………………………………………..

Date de naissance : …../……/……..

accepte librement et volontairement de participer à la recherche référencée ci-dessus, coordonnée par le Docteur Hérisson et organisée par le CHU de Nantes, promoteur de la recherche.

Etant entendu que :

- Le médecin qui m'a informé(e) et a répondu clairement à toutes mes questions, m'a précisé que ma participation est libre et que je peux me retirer de la recherche à tout moment.
- Il m'a été préalablement remis une lettre d'information sur cette recherche précisant son but, sa méthodologie, ses bénéfices attendus et ses risques prévisibles.
- Je pourrai avoir communication par le médecin, au cours ou à l'issue de la recherche, des informations qu'il détient concernant ma santé.
- J'ai bien compris dans la lettre d'information qui m'a été remise que pour pouvoir participer à cette recherche
- Je dois être affilié(e) ou bénéficier d'un régime de sécurité sociale. Je confirme que c'est bien le cas.
- Je suis parfaitement conscient(e) que je peux retirer à tout moment mon consentement à ma participation à cette recherche et cela quelles que soient mes raisons et sans supporter aucune responsabilité, mais je m'engage dans ce cas à en informer le médecin. Le fait de ne plus participer à cette recherche ne portera pas atteinte à mes relations avec ce médecin, ni à la qualité des soins qui me seront donnés.
- Je pourrai à tout moment demander des informations complémentaires au médecin.
- Si je le souhaite, à son terme, je serai informé(e) par le médecin des résultats globaux de cette recherche.
- Mon consentement ne décharge en rien le médecin et le promoteur de l'ensemble de leurs responsabilités et je conserve tous mes droits garantis par la loi.
- *Je ne pourrai pas participer à une autre recherche biomédicale pendant toute ma participation à cette recherche.*
- *J'accepte que les données enregistrées à l'occasion de cette recherche puissent faire l'objet d'un traitement informatisé par le promoteur ou pour son compte. J'ai bien noté que le droit d'accès prévu par la CNIL (loi du 6 janvier 1978 modifiée relative à l'informatique, aux fichiers et aux libertés (art. 39)) s'exerce à tout moment auprès du médecin qui me suit dans le cadre de la recherche et qui connaît mon identité. Je pourrai exercer mon droit de rectification et d'opposition auprès de ce même médecin, qui en informera le promoteur de la recherche.*
- *J'accepte que les personnes en charge du suivi de la recherche aient accès aux données de mon dossier médical*

Date :
Signature du patient :

Signature du **médecin** qui atteste avoir pleinement expliqué à la personne signataire le but, les modalités ainsi que les risques potentiels de la recherche.

Date :
Nom et Signature :

Ce document est à réaliser en 2 exemplaires originaux : le premier doit être conservé par l'investigateur et le deuxième est remis à la personne donnant son consentement. En cas de duplicata, l'original est conservé par l'investigateur et une copie est remise à la personne ayant donné son consentement
