# Supplementary material for: Early Sitting in Ischemic Stroke Patients (SEVEL): A Randomized Controlled Trial
Source: PLoS One. 2016 Mar 29;11(3):e0149466. doi: 10.1371/journal.pone.0149466 (PMC4811411; doi:10.1371/journal.pone.0149466)
Supplement: S7 Protocol — (PDF) [file pone.0149466.s008.pdf]

ANNEXE 9: FORMULAIRE DE RECUEIL DE CONSENTEMENT EN SITUATION D'URGENCE

« SEVEL : Verticalisation des patients à la phase aigue à la suite d'un infarctus cérébral »

Promoteur : CHU Nantes n°ID RCB: n°2011-A0043 0-41

Je soussigné(e)

Me, Mlle, M. (rayer les mentions inutiles) (prénom, nom) ………………………………………., signataire en tant que :

☐ personne de confiance ☐ membre de la famille ☐ personne entretenant avec l'intéressé des liens étroits et stables

J'accepte librement et volontairement que mon proche participe à la recherche référencée ci-dessus, coordonnée par le Docteur Hérisson et organisée par le CHU de Nantes, promoteur.

Etant entendu que :

- Son état le permettant, mon proche a été consulté et n'a pas exprimé de refus
- Le médecin qui m'a informé(e) et a répondu clairement à toutes mes questions, m'a précisé que la participation de mon proche est libre et que je peux retirer mon accord sur sa participation à tout moment.
- Il m'a été remis une lettre d'information sur cette recherche précisant son but, sa méthodologie, ses bénéfices attendus et ses risques prévisibles.
- Mon proche sera informé et son consentement recherché dès que cela sera possible
  Je pourrai avoir communication par le médecin, au cours ou à l'issue de la recherche, des informations qu'il détient concernant la santé de mon proche.
- J'ai bien compris que pour pouvoir participer à cette recherche mon proche dois être affilié(e) ou bénéficier d'un régime de sécurité sociale. Je confirme que c'est bien le cas.
- Je suis parfaitement conscient(e) que je peux arrêter la participation de mon proche à cette recherche et cela quelles que soient mes raisons et sans supporter aucune responsabilité, mais je m'engage dans ce cas à en informer le médecin. Le fait de ne plus participer à cette recherche ne portera pas atteinte à mes relations avec ce médecin, ni à la qualité des soins qui seront donnés à mon proche.
- Je pourrai à tout moment demander des informations complémentaires au médecin.
- Si je le souhaite, à son terme, je serai informé(e) par le médecin des résultats globaux de cette recherche.
- Mon consentement ne décharge en rien le médecin et le promoteur de l'ensemble de leurs responsabilités et mon proche conserve tous ses droits garantis par la loi.

- *Mon proche ne pourra pas participer à une autre recherche biomédicale pendant toute sa participation à cette recherche*
- *J'accepte que les données enregistrées à l'occasion de cette recherche puissent faire l'objet d'un traitement informatisé par le promoteur ou pour son compte. J'ai bien noté que le droit d'accès prévu par la CNIL (loi du 6 janvier 1978 modifiée relative à l'informatique, aux fichiers et aux libertés (art. 39)) s'exerce à tout moment auprès du médecin qui suit mon proche dans le cadre de la recherche et qui connaît son identité. Un droit de rectification et d'opposition pourra être exercé auprès de ce même médecin, qui en informera le promoteur de la recherche*
- *J'accepte que les personnes en charge du suivi de la recherche aient accès aux données de mon dossier médical*

Date :
Signature du représentant du patient :

Signature du **médecin** qui atteste avoir pleinement expliqué au représentant du patient le but, les modalités ainsi que les risques potentiels de la recherche.

Date :
Nom et Signature :

Dès que possible, le patient sera informé et son consentement sera recherché.

Ce document est à réaliser en 2 exemplaires originaux : le premier doit être conservé par l'investigateur et le deuxième est remis à la personne donnant son consentement. En cas de duplicata, l'original est conservé par l'investigateur et une copie est remise à la personne ayant donné son consentement
