# Supplementary material for: Early Sitting in Ischemic Stroke Patients (SEVEL): A Randomized Controlled Trial
Source: PLoS One. 2016 Mar 29;11(3):e0149466. doi: 10.1371/journal.pone.0149466 (PMC4811411; doi:10.1371/journal.pone.0149466)
Supplement: S1 Dataset — (DOCX) [file pone.0149466.s009.docx]

**TABLE**

**Description of the 29 excluded patients**

|  | Total (n=29) |
| --- | --- |
| Age (mean ±SD)  Median (Q1-Q3) | 71.19 ±12.46  69.75 [64.12-81.43] |
| Male (n,%) | 16 (55.17) |
| Pre admission Rankin score 0 (n, %) | 22 (75.86) |
| At home before hospitalization (n,%) | 29 (100) |
| *Cardiovascular risk factors* |  |
| High blood pressure (n,%) | 20 (68.97) |
| Diabetes (n,%) | 4 (13.79) |
| Dyslipidemia (n,%) | 13 (44.83) |
| Current or past smoking (n,%) | 8 (27.59) |
| BMI>30 (n,%) | 4 (18.18) |
| *Cardiovascular comorbidity* |  |
| Arteritis (n,%) | 2 (6.9) |
| Coronaropathy (n,%) | 6 (20.69) |
| *Qualifying event* |  |
| Admission NIHSS (mean ±SD)  Median (Q1-Q3) | 8.24±5.22  6 [4-10] |
| Hemiplegia (n,%) | 7 (24.14) |
| Aphasia (n,%) | 10 (34.48) |
| Rankin score [0-2] (n,%) | 8 (27.59) |
